# Divergent Elementoboration: 1,3‐Haloboration versus 1,1‐Carboboration of Propargyl Esters

**DOI:** 10.1002/chem.201801493

**Published:** 2018-04-27

**Authors:** Lewis C. Wilkins, Yashar Soltani, James R. Lawson, Ben Slater, Rebecca L. Melen

**Affiliations:** ^1^ School of Chemistry Cardiff University, Main Building Park Place Cardiff CF10 3AT Cymru/Wales UK; ^2^ Department of Chemistry University College London 20 Gordon Street London WC1H 0AJ UK

**Keywords:** alkynes, boranes, boron, haloboration, Lewis acid

## Abstract

This work showcases the 1,3‐haloboration reaction of alkynes in which boron and chlorine add to propargyl systems in a proposed sequential oxazoliumborate formation with subsequent ring‐opening and chloride migration. In addition, the functionalization of these propargyl esters with dimethyl groups in the propargylic position leads to stark differences in reactivity whereby a formal 1,1‐carboboration prevails to give the 2,2‐dichloro‐3,4‐dihydrodioxaborinine products as an intramolecular chelate. Density functional theory calculations are used to rationalize the distinct carboboration and haloboration pathways. Significantly, this method represents a metal‐free route to highly functionalized compounds in a single step to give structurally complex products.

The activation of carbon–carbon double and triple bonds by main group compounds has been a staple motif in the synthesis of a plethora of new element–carbon bonds such as C−C,^1^ C−H,^2^ C−N,^3^ C−B,^4^ and C−O^5^ bonds amongst many others.^6^ Seminal work by Wrackmeyer et al. showcased a powerful methodology using trivalent boranes in conjunction with “activated” alkynes, that is, M‐C≡C‐R, where M=Si, Ge, Sn, Pb inter alia.^7^ In these early cases, 1,1‐carboboration reactions were observed whereby a 1,2‐alkyl/aryl shift occurs between the distal and proximal carbons of the alkyne with the concomitant 1,2‐shift of the R group from boron to carbon (Scheme 1, top). Further to this, Erker has demonstrated extensive use of the carboboration mechanism to affect a number of complex rearrangement processes such as benzannulations^8^ and cyclizations,^9^ amongst others.^10^

**Scheme 1:**
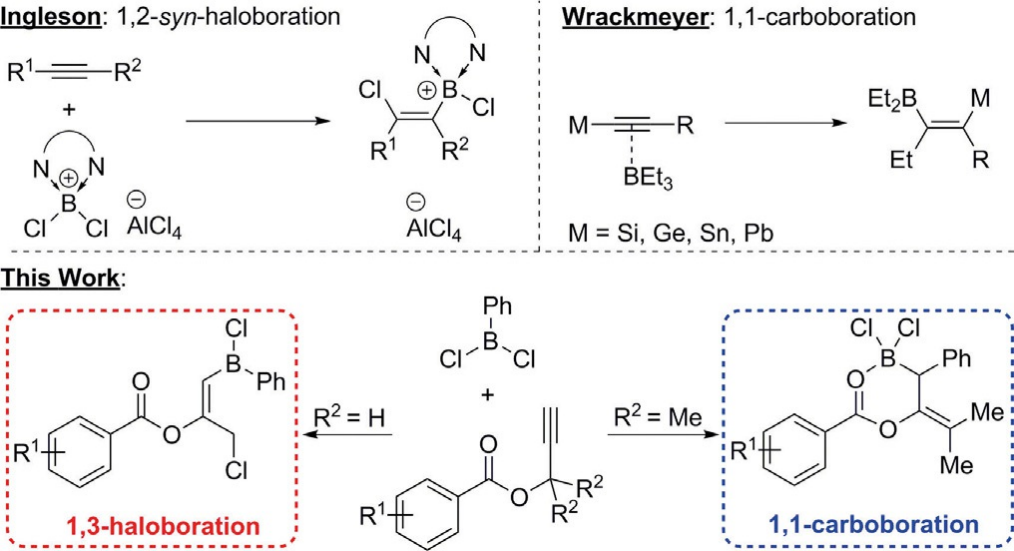
Background and overview to this work.

More recent work in such elementoboration reactions are seen through the synthetically useful haloboration reaction whereby a halogen, predominantly chlorine, is installed typically through a 1,1‐ or 1,2‐haloboration to yield the corresponding halovinylboronic ester (Scheme 1, top). The formation of these species has been generated through the use of simple haloboranes such as BX_3_ (X=Cl, Br), or borocations developed by Ingleson et al. In the case of borocations, stereoselective control is observed to give predominantly the *syn*‐addition product.^11^ Interestingly, a similar study showcased a sequential alkyne addition to affect a formal 1,4‐haloboration whereby phenylacetylene undergoes a 1,2‐addition when exposed to [LutBCl_2_][AlCl_4_] which, upon addition of various 1‐trimethylsilylalkynes, undergoes a subsequent 1,2‐carboboration to yield the diene product.11d All such haloboration reactions are convenient synthetic protocols to append functional groups to olefins, specifically in the formation of tri‐ and tetra‐substituted alkenes through subsequent cross‐coupling reactions of the boronic ester.^12^ Another aspect of the reaction outlined within is the installation of a pendant alkyl chloride (Scheme 1, bottom), which has countless uses within organic chemistry from reactions with acetylides, alkoxides, and cyanates, as well as Grignard chemistry.

Although a significant amount of research has focused on the sterically encumbered, strong Lewis acid, B(C_6_F_5_)_3_, as well as others of a similar nature,^13^ other commercially available boranes have seemingly been absent from recent studies. Hence, this work aims to reinvigorate the use of such boranes in a range of synthetically imperative transformations.

Herein, we show how subtle adaptations to the alkyne starting material can dramatically alter the reactivity with the borane reagent to give the stereoselective *trans*‐product of a formal 1,3‐haloboration, or alternatively a complex 1,1‐carboboration mechanism to yield a stable dichlorodihydrodioxaborinine heterocycle all in very good to excellent conversions. Importantly, these reagents are then well positioned to undergo further functionalization such as cross‐couplings^14^ or allylations.^15^

Initial investigations of the commercially available PhBCl_2_ used the model substrate **1 a** in a 1:1 stoichiometric ratio to yield a single product in near quantitative yields as observed using in situ multinuclear NMR spectroscopy. Detailed NMR spectroscopy (HSQC, HMBC) revealed the proposed structure of **3 a**, which interestingly is the product of a formal 1,3‐haloboration reaction. These encouraging initial results then led us to expand the substrate scope to a series of phenyl substituted propargyl esters (Figure 1, Scheme 2). It was observed that in most cases the target haloboration product could be clearly identified with conversions greater than 95 % at ambient temperature with reaction times of 8 h (**3 a**), 18 h (**3 b**,**c**), and 48 h (**3 d**).

**Figure 1:**
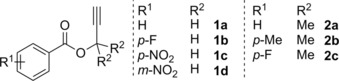
Propargyl ester substrates used in this work.

**Scheme 2:**
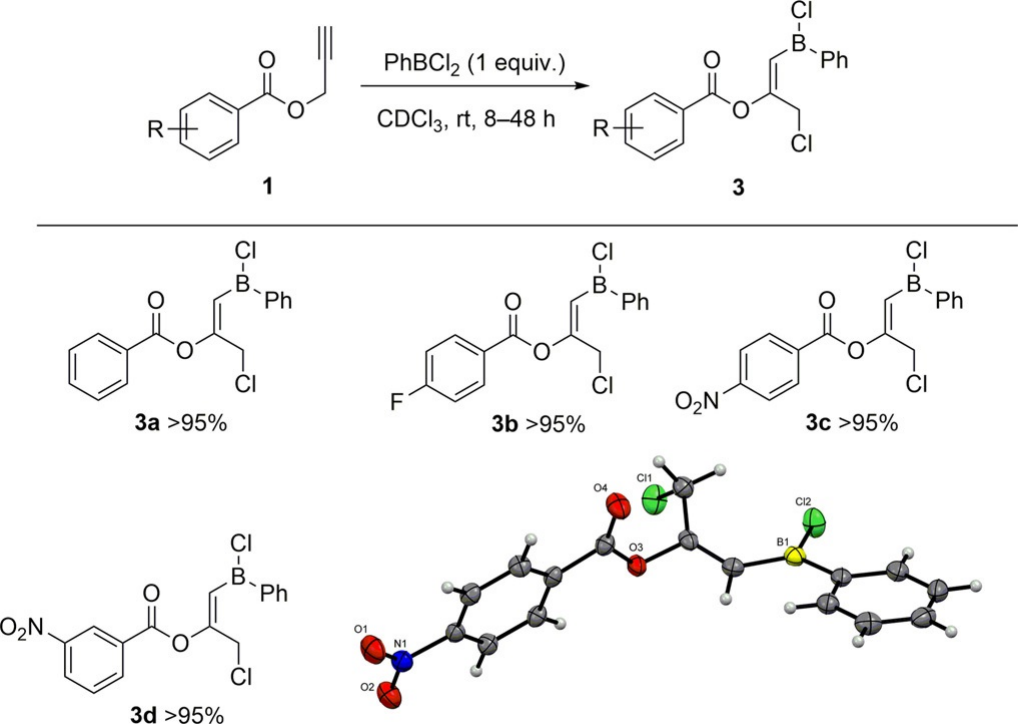
Reaction between PhBCl_2_ and **1** to give 1,3‐haloboration products **3**. Values are given as in situ NMR conversions. Solid‐state structure of compound **3 c**, C: grey, H: white, N: blue, O: red, B: yellow‐green, Cl: green. Thermal ellipsoids shown at 50 % probability (inset).

Fortunately, the storage of a saturated CH_2_Cl_2_/hexane solution of **3 c** at −40 °C produced a crop of crystals suitable for X‐ray diffraction. The structure was unambiguously determined to be indeed the product of a formal 1,3‐haloboration agreeing with spectroscopic analyses (Figure 2). Metrics of the solid‐state structure are as expected with the stereochemical conformation being determined as the *trans* product. Earlier work by Erker showcased the ability of vinylboranes to undergo photoinduced interconversion between the *E*/*Z* conformers upon exposure to UV light;^16^ thus, it was hoped similar reactivity could be observed here to yield the intramolecular chelate. However, no such species could be detected in the ^11^B NMR post‐irradiation, leaving the spectra identical to that of the non‐irradiated product **3**.

**Figure 2:**
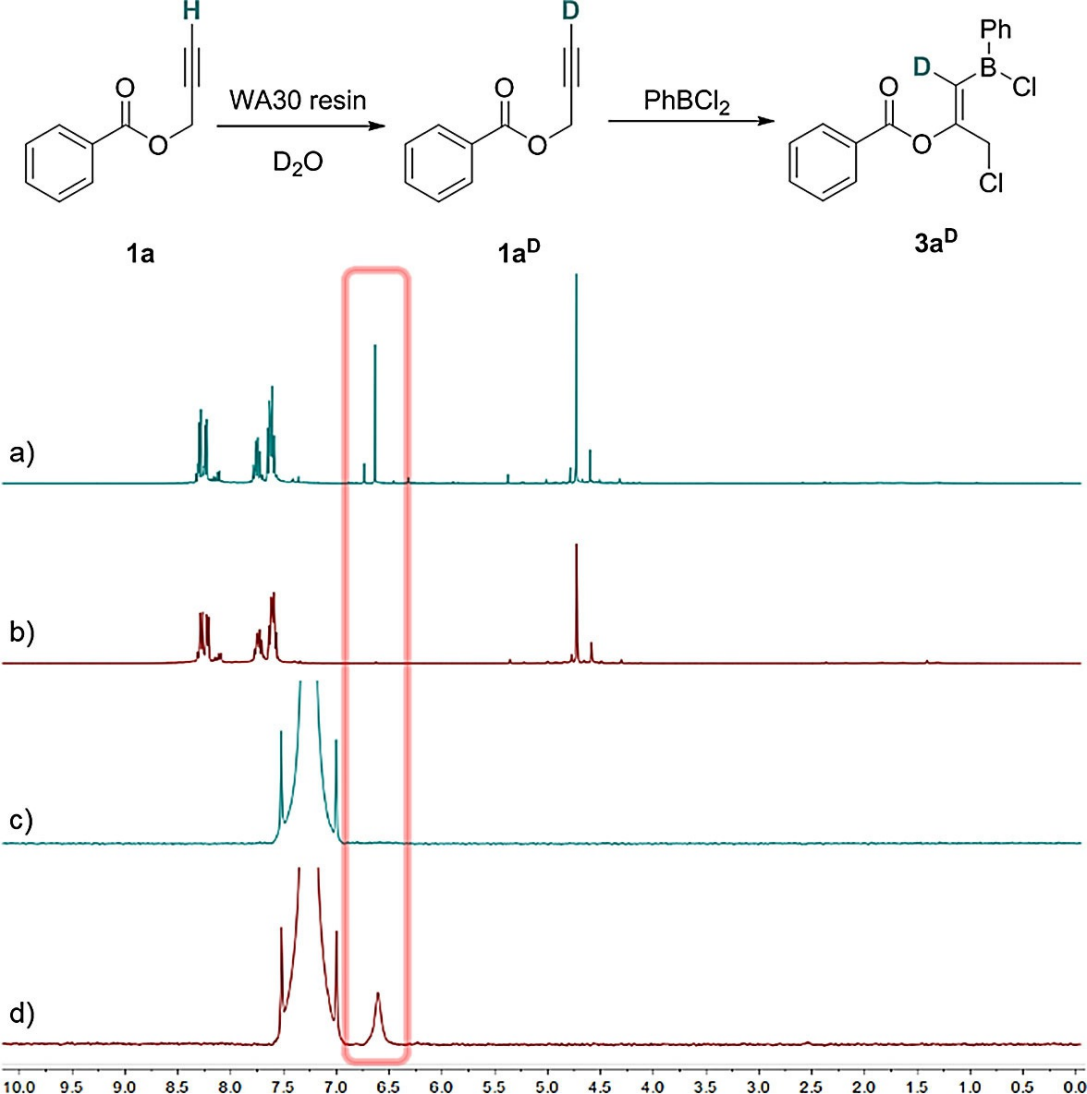
Stacked in situ spectra for the reaction between propargyl ester **1 a** or **1 a^D^** and PhBCl_2_ to yield a) **3 a** (^1^H); b) **3 a^D^** (^1^H); c) **3 a** (^2^H); d) **3 a^D^** (^2^H).

Conducting the reaction between PhBCl_2_ and **1 a** in a variation of solvents ([D_6_]benzene, [D_8_]toluene, CH_2_Cl_2_, C_6_H_5_Cl) appeared to make no difference in reactivity with all showing almost quantitative conversion within 9 hours. Additionally, trialling other boron reagents such as BCl_3_ as well as the borocation [PhClB(2‐DMAP)][AlCl_4_]11a were unsuccessful with a mixture of products prevailing as observed in the resultant multinuclear NMR spectra.

To gain some insight into the proposed mechanism, isotopic labeling studies were performed. The terminal position of the alkyne was deuterated selectively using an amine appended resin (WA50) in accordance with the literature.^17^ Tracking the reaction progress using both ^1^H and ^2^H NMR spectroscopy whilst comparing the in situ data of the protic versus deuterated compounds shed light on the fate of the terminal alkynyl hydrogen atom and hence the reaction mechanism (see below, Scheme 4). A ^1^H resonance at *δ*=6.6 ppm is observed in **3 a** for the proton on the carbon adjacent to boron, which is evidently absent in **3 a^D^** (Figure 2). Additionally, following the ^2^H NMR spectra of the reactions using **1 a** and **1 a^D^** clearly shows the alkyne resonance at *δ*=2.5 ppm diminishing in intensity with the commensurate appearance of the previously identified new resonance at *δ*=6.6 ppm.

Further derivatization of the starting materials to include methyl groups in the propargylic position was undertaken to yield compounds **2 a**–**2 c** (Figure 1). Upon exposure of **2** to a stoichiometric amount of PhBCl_2_, new resonances in the ^1^H and ^11^B NMR spectra were noted after 8 h at 45 °C, which, interestingly, were not consistent with the 1,3‐haloboration products **3** from reagents **1**. Indeed, a broad singlet resonance was observed in the ^1^H NMR spectrum at ca. *δ*=3.8 ppm alongside a sharp singlet resonance in the ^11^B NMR spectrum at ca. *δ*=8 ppm indicating the formation of a chelating dioxaborinine type structure as seen in Scheme 3. This was further expounded by means of the ^13^C NMR spectra with the presence of a new *sp* ^3^ carbon adjacent to boron presenting a resonance at ca. *δ*=40 ppm versus 120 ppm for the adjacent sp^2^ carbon in **3**. Storing **4 a**–**c** as a saturated CH_2_Cl_2_/hexane solution produced a number of colorless crystals suitable for X‐ray diffraction, which indeed determined the molecular structure to be the product of a formal 1,1‐carboboration reaction (Figure 3). Of particular note is the regioselectivity of this reaction with the product predominating as the selective transfer of the aryl group over the chloride fragment.^18^ This migration pattern could be confirmed once again through detailed 2D NMR spectroscopy to affirm the molecular connectivity revealed in the solid‐state structure (see the Supporting Information).

**Scheme 3:**
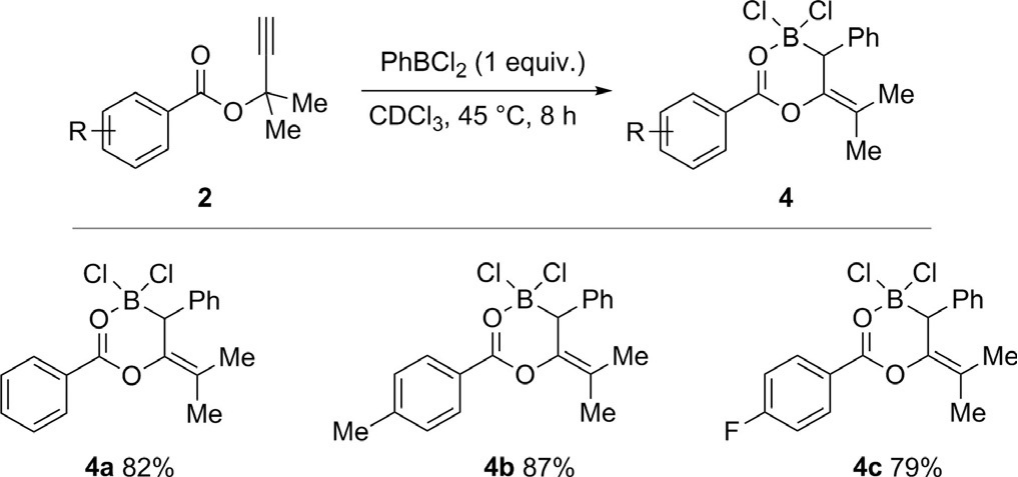
Reaction between PhBCl_2_ and **2** to give 1,1‐carboboration products **4**. Yields are given as isolated yields.

**Figure 3:**
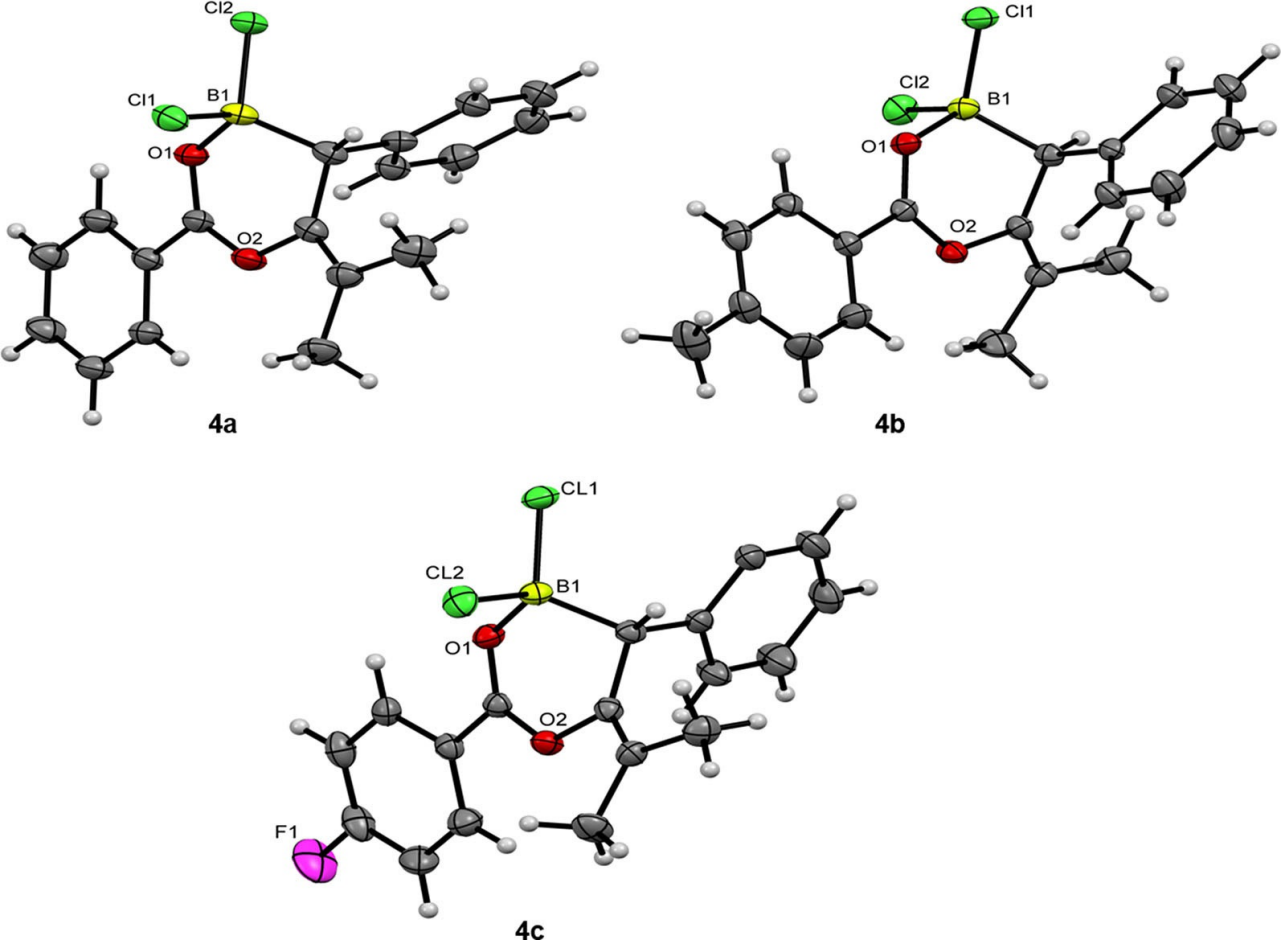
Solid‐state structure of compounds **4 a**–**c**, C: grey, H: white, O: red, B: yellow‐green, Cl: green, F: pink. Thermal ellipsoids shown at 50 % probability.

When comparing the divergent elementoboration observed here, it is proposed that the inclusion of non‐H groups in the propargylic position must play a critical role in which pathway is undertaken in this reaction. Mechanistically we propose that an initial 1,2‐*trans*‐oxyboration step occurs to yield the zwitterionic dioxolium borate.^19^ During the formation of this 5‐membered dioxolium intermediate (**I**, Scheme 4), when simple hydrogen atoms occupy the R^2^ position, the formation of **3** is slightly more favorable compared to when methyl groups are included in the R^2^ position. Conversely, if more bulky methyl groups are included, then the chloride migration pathway is less favored over 1,2‐aryl group migration resulting in the generation the intramolecular chelate **4**. These experimental findings are supported through in silico studies (see below and the Supporting Information). Additionally, when using compound **5**, which features a combination of H and Me in the propargyl position, a more complex transformation is observed when monitoring the reaction coordinate over time. Analyzing the in situ ^1^H and ^11^B NMR spectra suggests that, after initial combination of PhBCl_2_ with **5** in a 1:1.2 ratio, the haloboration product **6** prevails, as observed by the characteristic broad singlet at *δ*=6.35 ppm alongside the formation of a resonance at ca. *δ*=5.2 ppm for the proposed vinyl and methylene protons, respectively (see the Supporting Information). Over time, these resonances reduce in intensity giving way to a new broad singlet at *δ*=3.36 ppm, consistent with the generation of the proton in the adjacent to the borane in the chelating structure **7**. In addition, new resonances appear for the newly formed vinyl proton quartet at *δ*=5.45 ppm, and the methyl doublet at *δ*=1.90 ppm. This assertion is bolstered when observing the in situ ^11^B NMR spectra over time whereby the expected singlet at about *δ*=55.1 ppm forms after 1 h at ambient temperature, which reduces in intensity over time yielding another singlet resonance at ca. *δ*=9.1 ppm, again indicating the reversible formation of **6** en route to **7** (Scheme 5).

**Scheme 4:**
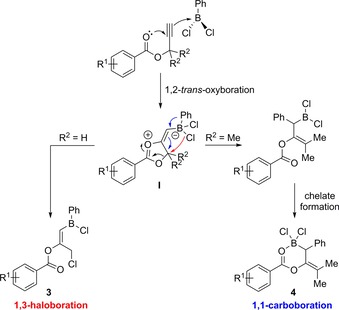
Proposed mechanism for the divergent elementoboration of **1** and **2** using PhBCl_2_.

**Scheme 5:**
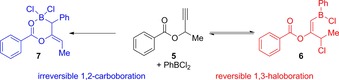
Conversion of **5** to **6** and **7** via a proposed reversible 1,3‐haloboration or 1,2‐carboboration mechanism.

To shed light on the divergent reactivity realized in this work, DFT calculations were performed. The reaction pathways for haloboration (upper) and carboboration (lower) are displayed in Figure 4. Pleasingly, the formation of products **3** and **4** proceeds in line with the proposed Scheme 4 via the key dioxolium intermediate **I**. Once the intermediate **I** is formed, chloride migration is the transition state for the haloboration reaction and phenyl migration is the rate‐determining transition state for the carboboration reaction. It is clear from Figure 4, upon comparison of the pathways, that carboboration (R^2^=Me) is strongly thermodynamically preferred over haloboration (R^2^=H). Intriguingly, however, we found that the hypothetical carboboration product (see the Supporting Information, Table S1) for R^2^=H is >30 kcal mol^−1^ more stable than the R^2^=H haloboration product obtained experimentally, raising the question: why does the carboboration reaction not occur for R^2^=H?

**Figure 4:**
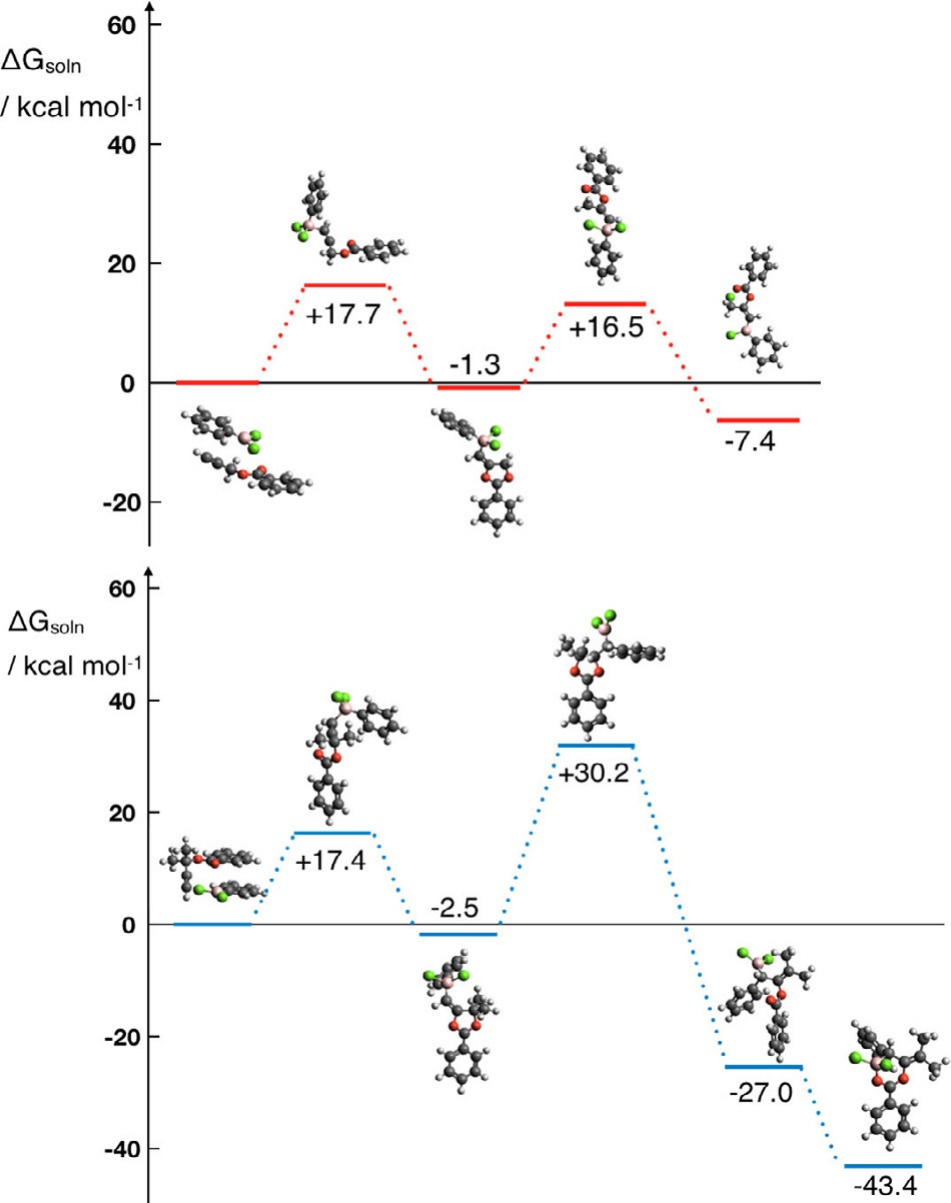
Free energy diagram comparing the mechanism of the haloboration (red) and carboboration (blue) reactions yielding products **3** and **4** respectively.

After 1,2‐*trans*‐oxyboration and formation of the intermediate dioxolium intermediate **I**, two pathways are available: 1) chloride migration that results in a metastable haloboration product or 2) phenyl migration that yields the thermodynamically favored carboboration product. The carboboration reaction of **1 a** is disfavored for two reasons: first, following the kinetically favored pathway, chloride migration occurs yielding the haloboration product and reversion back to the dioxolium **I** is strongly hindered by a high reverse barrier (Δ*G*_gas_= +28.9 kcal mol^−1^) from the product. Second, in order to yield the carboboration product from **I**, a prohibitively large barrier for phenyl migration (Δ*E*_gas_=+37.20 kcal mol^−1^) must be overcome (see the Supporting Information, sections 3.2.3 and 3.2.4). For comparison, the phenyl migration barrier for compounds **2 a** (R^2^=Me) is 9 kcal mol^−1^ lower than for **1 a** (R^2^=H). Hence, for **1 a** (R^2^=H), the kinetically favored haloboration product is formed. Conversely, the haloboration product of **2 a** (R^2^=Me) has a comparatively low reverse barrier from the product (Δ*G*_gas_=+23.3 kcal mol^−1^) and also a low barrier for phenyl migration (Δ*E*_gas_=+28.2 kcal mol^−1^) in comparison to that for **1 a** (R^2^=H). Hence, any haloboration product formed from starting materials **2 a** may revert to **I** fleetingly and phenyl migration will occur. Once phenyl migration occurs, there is a strong free energy incentive to form the thermodynamically preferred chelate **4**, which has a very prohibitive >+70 kcal mol^−1^ barrier (Δ*G*_sol_) to revert to **I**.

Intriguingly, in line with the observed reversible behavior of the mono‐methylate **5** to form haloboration product **6** and then the carboboration product **7** (see Scheme 5), calculations show a reverse barrier from the product of Δ*G*_gas_=+25.8 kcal mol^−1^, which is intermediate between those found for R^2^=H and R^2^=Me and an intermediate barrier for phenyl migration of Δ*E*_gas_=+29.2 kcal mol^−1^. Hence, according to mechanistic and energetic considerations, the mono‐methylate should form a more kinetically stable haloboration product than bi‐methylate and is therefore more likely to be isolable under comparable reaction conditions, exactly in line with our findings. The phenyl migration barrier is lower for R^2^=Me, H than R^2^=H, so the carboboration product is more likely to be isolable, again in agreement with our observations in Scheme 5. These findings show that **3** and **6** are more kinetically stable than the haloborated product of **2 a** (R^2^=Me), explaining why both **3** and **6** are observed. The relatively low barriers for phenyl migration to form **4** and **7** with the strong thermodynamic driving force explain why the carboboration products occur for R^2^=Me and R^2^=Me, H. Overall, these calculations reveal a remarkably subtle interplay of kinetic and thermodynamic factors that are acutely sensitive to the R^2^ groups and which cause profoundly different reaction products.

In summary, this work has shown both the formal 1,1‐carboboration as well as formal 1,3‐haloboration of alkynes can occur through simple tuning of the alkyne starting material being used. All of these multi‐step reactions proceed cleanly with high conversions and yields being noted in a one‐pot, atom efficient manner, garnering synthetically useful and functionally diverse compounds for further reactivity. In depth computational studies have helped elucidate the proposed mechanism that differentiates this divergent elementoboration.

## Conflict of interest

The authors declare no conflict of interest.

## Supporting information

As a service to our authors and readers, this journal provides supporting information supplied by the authors. Such materials are peer reviewed and may be re‐organized for online delivery, but are not copy‐edited or typeset. Technical support issues arising from supporting information (other than missing files) should be addressed to the authors.

Supplementary

## References

[1a] J. S. Reddy , B.-H. Xu , T. Mahdi , R. Fröhlich , G. Kehr , D. W. Stephan , G. Erker , Organometallics 2012, 31, 5638–5649;

[1b] M. M. Hansmann , R. L. Melen , M. Rudolph , F. Rominger , H. Wadepohl , D. W. Stephan , A. S. K. Hashmi , J. Am. Chem. Soc. 2015, 137, 15469–15477; 26580316 10.1021/jacs.5b09311

[1c] Y. Soltani , L. C. Wilkins , R. L. Melen , Angew. Chem. Int. Ed. 2017, 56, 11995–11999; 10.1002/anie.201704789 28703388

[2a] K. Chernichenko , Á. Madarász , I. Pápai , M. Nieger , M. Leskelä , T. Repo , Nat. Chem. 2013, 5, 718–723; 23881505 10.1038/nchem.1693

[2b] L. J. Hounjet , D. W. Stephan , Org. Process Res. Dev. 2014, 18, 385–391;

[2c] J. R. Lawson , L. C. Wilkins , R. L. Melen , Chem. Eur. J. 2017, 23, 10997–11000; 28686789 10.1002/chem.201703109 PMC5577513

[2d] Q. Yin , Y. Soltani , R. L. Melen , M. Oestreich , Organometallics 2017, 36, 2381–2384.

[3] J. S. Wixey , B. D. Ward , Chem. Commun. 2011, 47, 5449–5451. 10.1039/c1cc11229e 21465058

[4a] W. E. Piers , in Advances in Organometallic Chemistry, Vol. 52, Academic Press, Cambridge, 2004, pp. 1–76;

[4b] R. L. Melen , M. M. Hansmann , A. J. Lough , A. S. K. Hashmi , D. W. Stephan , Chem. Eur. J. 2013, 19, 11928–11938; 23922200 10.1002/chem.201301899

[4c] A. Feldmann , G. Kehr , C. G. Daniliuc , C. Muck-Lichtenfeld , G. Erker , Chem. Eur. J. 2015, 21, 12456–12464. 26284948 10.1002/chem.201502278

[5] D. J. Faizi , A. Issaian , A. J. Davis , S. A. Blum , J. Am. Chem. Soc. 2016, 138, 2126–2129. 26849770 10.1021/jacs.5b12989 PMC4768685

[6a] J. M. Bayne , D. W. Stephan , Chem. Soc. Rev. 2016, 45, 765–774; 26255595 10.1039/c5cs00516g

[6b] A. Issaian , K. N. Tu , S. A. Blum , Acc. Chem. Res. 2017, 50, 2598–2609. 28933550 10.1021/acs.accounts.7b00365

[7] B. Wrackmeyer , Coord. Chem. Rev. 1995, 145, 125–156.

[8] R. Liedtke , F. Tenberge , C. G. Daniliuc , G. Kehr , G. Erker , J. Org. Chem. 2015, 80, 2240–2248. 25581248 10.1021/jo502753s

[9] C. Chen , M. Harhausen , R. Liedtke , K. Bussmann , A. Fukazawa , S. Yamaguchi , J. L. Petersen , C. G. Daniliuc , R. Fröhlich , G. Kehr , G. Erker , Angew. Chem. Int. Ed. 2013, 52, 5992–5996; 10.1002/anie.201300871 23649543

[10] G. Kehr , G. Erker , Chem. Sci. 2016, 7, 56–65. 28757997 10.1039/c5sc03282b PMC5508682

[11a] M.-L. Yao , M. S. Reddy , W. Zeng , K. Hall , I. Walfish , G. W. Kabalka , J. Org. Chem. 2009, 74, 1385–1387; 19093827 10.1021/jo802207y

[11b] J. R. Lawson , E. R. Clark , I. A. Cade , S. A. Solomon , M. J. Ingleson , Angew. Chem. Int. Ed. 2013, 52, 7518–7522; 10.1002/anie.201302609 PMC3749439 23740843

[11c] M. J. Ingleson , in Fundamental and Applied Properties of Borocations, Synthesis and Application of Organoboron Compounds, Springer, Cham, 2015, 39–71;

[11d] A. J. Warner , J. R. Lawson , V. Fasano , M. J. Ingleson , Angew. Chem. Int. Ed. 2015, 54, 11245–11249; 10.1002/anie.201505810 PMC4832827 26237115

[11e] J. R. Lawson , V. Fasano , J. Cid , I. Vitorica-Yrezabal , M. J. Ingleson , Dalton Trans. 2016, 45, 6060–6070. 26373445 10.1039/c5dt03003j

[12a] S. Hara , H. Dojo , S. Takinami , A. Suzuki , Tetrahedron Lett. 1983, 24, 731–734;

[12b] Y. Satoh , T. Tayano , H. Koshino , S. Hara , A. Suzuki , Synthesis 1985, 1985, 406–408;

[12c] Y. Satoh , H. Serizawa , S. Hara , A. Suzuki , J. Am. Chem. Soc. 1985, 107, 5225–5228;

[12d] M. Sato , Y. Yamamoto , S. Hara , A. Suzuki , Tetrahedron Lett. 1993, 34, 7071–7074.

[13] J. R. Lawson , R. L. Melen , Inorg. Chem. 2017, 56, 8627–8643. 28157303 10.1021/acs.inorgchem.6b02911

[14] G.-Z. Wang , J. Jiang , X.-S. Bu , J.-J. Dai , J. Xu , Y. Fu , H.-J. Xu , Org. Lett. 2015, 17, 3682–3685. 26181828 10.1021/acs.orglett.5b01612

[15a] H. C. Brown , P. K. Jadhav , J. Am. Chem. Soc. 1983, 105, 2092–2093;

[15b] W. R. Roush , L. K. Hoong , M. A. J. Palmer , J. A. Straub , A. D. Palkowitz , J. Org. Chem. 1990, 55, 4117–4126.

[16] C. Chen , T. Voss , R. Fröhlich , G. Kehr , G. Erker , Org. Lett. 2011, 13, 62–65. 21128629 10.1021/ol102544x

[17] T. Yamada , K. Park , Y. Monguchi , Y. Sawama , H. Sajiki , RSC Adv. 2015, 5, 92954–92957.

[18] L. C. Wilkins , J. R. Lawson , P. Wieneke , F. Rominger , A. S. K. Hashmi , M. M. Hansmann , R. L. Melen , Chem. Eur. J. 2016, 22, 14618–14624. 27538742 10.1002/chem.201602719

[19a] M. M. Hansmann , R. L. Melen , F. Rominger , A. S. K. Hashmi , D. W. Stephan , J. Am. Chem. Soc. 2014, 136, 777–782; 24354408 10.1021/ja4110842

[19b] M. M. Hansmann , R. L. Melen , F. Rominger , A. S. K. Hashmi , D. W. Stephan , Chem. Commun. 2014, 50, 7243–7245. 10.1039/c4cc01370k 24817134

