# Supplementary material for: Divergent Elementoboration: 1,3‐Haloboration versus 1,1‐Carboboration of Propargyl Esters
Source: Chemistry. 2018 Apr 27;24(29):7364–8. doi: 10.1002/chem.201801493 (PMC6055811; doi:10.1002/chem.201801493)
Supplement: Supplementary file 1 — Supplementary [file CHEM-24-7364-s001.pdf]

CHEMISTRY
A European Journal

Supporting Information

Divergent Elementoboration: 1,3-Haloboration versus 1,1-Carboboration of Propargyl Esters

Lewis C. Wilkins+,[a] Yashar Soltani+,[a] James R. Lawson,[a] Ben Slater,[b] and Rebecca L. Melen*[a]

chem_201801493_sm_miscellaneous_information.pdf

Contents

1 Experimental. 1
1.1 General experimental. 1
1.2 Synthesis of starting materials. 2
1.3 Synthesis of products. 4
2 NMR spectra. 7
2.1 Mechanistic studies. 7
2.2 NMR spectra of starting materials. 9
2.3 NMR spectra of products. 30
3 Computational 69
3.1 Computational settings 69
3.2 Reaction thermochemistry 69
3.3 Reactant and product geometries 73
4 Crystallographic studies. 95
References. 100

1 Experimental.

1.1 General experimental.

With the exception of the starting materials, all reactions and manipulations were carried out under an atmosphere of dry, O_2-free nitrogen using standard double-manifold techniques with a rotary oil pump. An argon- or nitrogen-filled glove box (MBraun) was used to manipulate solids including the storage of starting materials, room temperature reactions, product recovery and sample preparation for analysis. All solvents (toluene, CH_2Cl_2, hexane) were dried by employing a Grubbs-type column system (Innovative Technology) or a solvent purification system MB SPS-800 and stored under a nitrogen atmosphere. Deuterated solvents were distilled and/or dried over molecular sieves before use. Chemicals were purchased from commercial suppliers and used as received. ^{1}H, ^{13}C, ^{11}B and ^{19}F NMR spectra were recorded on a Bruker Avance II 400 or Bruker Avance 500 spectrometers. Chemical shifts are expressed as parts per million (ppm, δ) downfield of tetramethylsilane (TMS) and are referenced to $CDCl_3$ (7.26/77.16 ppm) as internal standards. NMR spectra were referenced to $CFCl_3$ (^{19}F) and $BF_3{\cdot}Et_2O/CDCl_3$ (^{11}B). The description of signals include: s = singlet, d = doublet, t = triplet, m = multiplet and br. = broad. All coupling constants are absolute values and are expressed in Hertz (Hz). ^{13}C NMR was measured as ^{1}H decoupled. Yields are given as isolated yields. IR-Spectra were measured on a Shimadzu IRAffinity-1 photospectrometer.

1.2 Synthesis of starting materials.

<u>*General Procedure 1:*</u> Synthesized using a procedure similar to that reported by Stephan *et al.*[1] The acyl chloride (15 mmol, 1.2 equiv.) and triethylamine (TEA) (3.5 ml, 25 mmol, 2 equiv.) were dissolved in 50 ml CH_2Cl_2 and cooled to 0 °C. Propargyl alcohol (0.73 ml, 12.5 mmol, 1 equiv.), 2-methylbut-3-yn-2-ol (1.21 ml, 12.5 mmol, 1 equiv.) or but-3-yn-2-ol (0.92 ml, 12.5 mmol, 1 equiv.) was added dropwise at this temperature and the mixture stirred at room temperature for 10 h. The resulting solution was washed with H_2O (1 × 100 ml) with the aqueous phase being extracted with CH_2Cl_2 (3 × 50 ml). The combined organic fractions were washed with saturated NaCl solution (1 × 100 ml) and dried over $MgSO_4$ with the volatiles being removed *in vacuo*. The crude product was purified by either crystallization or column chromatography.

Synthesis of prop-2-yn-1-yl benzoate (**1a**).

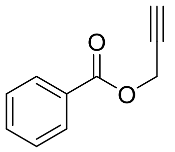

Synthesized according to *General Procedure 1* using benzoyl chloride (1.74 ml, 15 mmol, 1.2 equiv.). Yield 1.68 g, 10.5 mmol, 84%. Spectroscopic data agrees with literature values.[2] **^{1}H NMR** (400 MHz, $CDCl_3$, 298 K) δ/ppm: 8.08 (ddd, $^3J_{HH}$ = 6.8, $^4J_{HH}$ = 2.8, $^4J_{HH}$ = 1.5 Hz, 2H, Ar-H), 7.61 – 7.55 (m, 1H, Ar-H), 7.49 – 7.42 (m, 2H, Ar-H), 4.93 (d, $^4J_{HH}$ = 2.5 Hz, 2H, CH_2), 2.52 (t, $^4J_{HH}$ = 2.5 Hz, 1H, ≡CH). **^{13}C NMR** (101 MHz, $CDCl_3$, 298 K) δ/ppm: 165.9 (s), 133.5 (s), 129.9 (s), 129.5 (s), 128.6 (s), 77.9 (s), 75.1 (s), 52.6 (s).

Synthesis of prop-2-yn-1-yl 4-fluorobenzoate (**1b**).

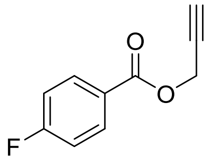

Synthesized according to *General Procedure 1* using 4-fluorobenzoyl chloride (1.77 ml, 15 mmol, 1.2 equiv.). Yield 1.87 g, 10.5 mmol, 84%. Spectroscopic data agrees with literature values.[3] **^{1}H NMR** (400 MHz, $CDCl_3$, 298 K) δ/ppm: 8.12 – 8.06 (m, 2H, Ar-H), 7.15 – 7.10 (m, 2H, Ar-H), 4.92 (d, $^4J_{HH}$ = 2.5 Hz, 2H, CH_2), 2.52 (t, $^4J_{HH}$ = 2.5 Hz, 1H, ≡CH). **^{13}C NMR** (101 MHz, $CDCl_3$, 298 K) δ/ppm: 166.1 (d, $^1J_{CF}$ = 255.2 Hz), 165.0 (s), 132.6 (d, $^3J_{CF}$ = 9.4 Hz), 125.8 (d, $^4J_{CF}$ = 3.0 Hz), 115.8 (d, $^2J_{CF}$ = 22.1 Hz), 77.7 (s), 75.3 (s), 52.7 (s). **^{19}F NMR** (376 MHz, $CDCl_3$, 298 K) δ/ppm: -104.9 (s).

Synthesis of prop-2-yn-1-yl 4-nitrobenzoate (**1c**).

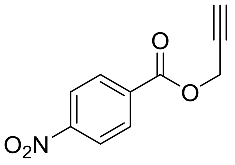

Synthesized according to *General Procedure 1* using *p*-nitrobenzoylchloride (2.77 g, 15 mmol, 1.2 equiv.). Crystallization *via* slow evaporation of CH_2Cl_2 gave the pure product as a yellow crystalline solid. Yield: 2.36 g, 11.5 mmol, 96%. Spectroscopic data agrees with literature values.[2] **^{1}H NMR** (500 MHz, $CDCl_3$, 298 K) δ/ppm: 8.30 (dt, $^3J_{HH}$ = 9.0 Hz, $^4J_{HH}$ = 2.2 Hz, 2H, Ar-H), 8.23 (dt, $^3J_{HH}$ = 9.0 Hz, $^4J_{HH}$ = 2.2 Hz, 2H, Ar-H), 4.97 (d, $^4J_{HH}$ = 2.5 Hz, 2H, CH_2), 2.56 (t, $^4J_{HH}$ = 2.5 Hz, 1H, ≡CH). **^{13}C NMR** (126 MHz, $CDCl_3$, 298 K) δ/ppm: 164.0 (s), 151.0 (s), 134.9 (s), 131.1 (s), 123.8 (s), 77.1 (s), 75.8 (s), 53.4 (s).

Synthesis of prop-2-yn-1-yl 3-nitrobenzoate (**1d**)

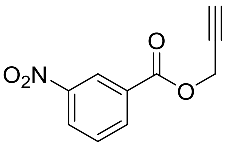

Synthesized according to *General Procedure 1* using *m*-nitrobenzoyl chloride (2.77 g, 15 mmol, 1.2 equiv.). Yield: 1.67 g, 8.13 mmol, 65%. Spectroscopic analyses agree with literature values.[4] **^{1}H NMR** (400 MHz, $CDCl_3$, 298 K) δ/ppm: 8.90 (dd, $^4J_{HH}$ = 2.9, $^4J_{HH}$ = 1.1 Hz, 1H, Ar-H), 8.45 (ddd, $^3J_{HH}$ = 8.2, $^4J_{HH}$ = 2.3, $^4J_{HH}$ = 1.1 Hz, 1H, Ar-H), 8.43 – 8.38 (m, 1H, Ar-H), 7.72 – 7.65 (m, 1H, Ar-H), 4.99 (d, $^4J_{HH}$ = 2.5 Hz, 2H, CH_2), 2.57 (t, $^4J_{HH}$ = 2.5 Hz, 1H, ≡CH). **^{13}C NMR** (101 MHz, $CDCl_3$, 298 K) δ/ppm: 163.9 (s), 148.4 (s), 135.6 (s), 131.3 (s), 129.9 (s), 127.9 (s), 125.0 (s), 77.1 (s), 75.9 (s), 53.4 (s).

Synthesis of 2-prop-3-yn-3-d-1-yl benzoate (**1a^D**).

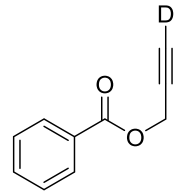

Synthesized using similar methods to that reported in the literature[5] whereby the proto-derivative **1a** (0.336 g, 2.1 mmol) was dissolved in D_2O at which point the WA30 resin (38 mg) was added and stirred under ambient conditions for 18 h. At this point the resin was filtered off, and the filtrate was extracted with Et_2O (10 ml). This was washed with water (10 ml) with the aqueous phase being extracted with Et_2O (3 x 10 ml). The combined organic phases were dried over $MgSO_4$ with the volatiles then being removed *in vacuo* to give the pure deuterated compound. Yield: 0.267 g, 1.87 mmol, 88%. Spectroscopic data agrees with literature values.[5] **^{1}H NMR** (400 MHz, $CDCl_3$, 298 K) δ/ppm: 8.02 – 7.97 (m, 2H, Ar-H), 7.53 – 7.48 (m, 1H, Ar-H), 7.40 – 7.34 (m, 2H, Ar-H), 4.85 (s, 2H, CH_2). **^{2}H NMR** (61 MHz, $CDCl_3$, 298 K) δ/ppm: 2.53 (s, ≡CD). **^{13}C NMR** (101 MHz, $CDCl_3$, 298 K) δ/ppm: 165.9 (s), 133.5 (s), 130.0 (s), 129.5 (s), 128.6 (s), 77.5 (s), 74.9 (t, $^1J_{CD}$ = 39 Hz), 52.6 (s).

Synthesis of 2-methylbut-3-yn-2-yl benzoate (**2a**).

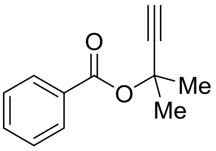

Synthesized according to *General Procedure 1* using benzoyl chloride (1.74 ml, 15 mmol, 1.2 equiv.). Yield: 0.352 g, 1.89 mmol, 15%. Yield: 0.245 g, 1.30 mmol, 15%. Spectroscopic data agrees with literature known values.[6] **^{1}H NMR** (400 MHz, $CDCl_3$, 298 K) δ/ppm: 8.05 – 8.01 (m, 2H, Ar-H), 7.57 – 7.52 (m, 1H, Ar-H), 7.46 – 7.40 (m, 2H, Ar-H), 2.59 (s, 1H, ≡CH), 1.82 (s, 3H, CH_3). **^{13}C NMR** (101 MHz, $CDCl_3$, 298 K) δ/ppm: 165.0 (s), 133.1 (s), 130.9 (s), 129.8 (s), 128.4 (s), 84.8 (s), 72.7 (s), 72.4 (s), 29.2 (s).

Synthesis of 2-methylbut-3-yn-2-yl benzoate (**2b**).

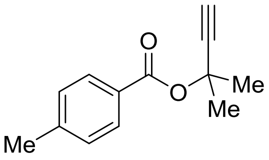

Synthesized according to *General Procedure 1* using 4-toluoyl chloride (1.98 ml, 15 mmol, 1.2 equiv.). Yield: 1.31 g, 6.5 mmol, 52%. Spectroscopic data agrees with literature known values.[1] **^{1}H NMR** (400 MHz, $CDCl_3$, 298 K) δ/ppm: 7.90 (d, $^3J_{HH}$ = 8.2 Hz, 2H, Ar-H), 7.21 (d, $^3J_{HH}$ = 8.1 Hz, 2H, Ar-H), 2.57 (s, 1H, ≡CH), 2.38 (s, 3H, Ar-CH_3), 1.80 (s, 6H, CH_3). **^{13}C NMR** (101 MHz, $CDCl_3$, 298 K) δ/ppm: 164.0 (s), 142.6 (s), 128.7 (s), 128.0 (s), 127.1 (s), 83.9 (s), 71.5 (s), 71.0 (s), 28.1 (s), 20.7 (s).

Synthesis of 2-methylbut-3-yn-2-yl benzoate (**2c**).

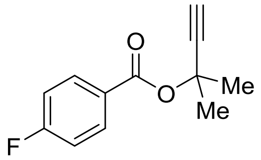

Synthesized according to *General Procedure 1* using 4-fluorobenzoyl chloride (1.77 ml, 15 mmol, 1.2 equiv.). Yield: 1.78 g, 8.62 mmol, 69%. **^{1}H NMR** (400 MHz, $CDCl_3$, 298 K) δ/ppm: 8.05 – 8.01 (m, 2H, Ar-H), 7.11 – 7.06 (m, 2H, Ar-H), 2.59 (s, 1H, ≡CH), 1.81 (s, 6H, CH_3). **^{13}C NMR** (101 MHz, $CDCl_3$, 298 K) δ/ppm: 165.8 (d, $^1J_{CF}$ = 253.7 Hz), 164.0 (s), 132.3 (d, $^3J_{CF}$ = 9.3 Hz), 127.1 (d, $^4J_{CF}$ = 3.0 Hz), 115.5 (d, $^2J_{CF}$ = 22.0 Hz), 84.7 (s), 72.8 (s), 72.5 (s), 29.1 (s). **^{19}F NMR** (376 MHz, $CDCl_3$, 298 K) δ/ppm: -105.8 (s).

Synthesis of but-3-yn-2-yl benzoate (**5**).

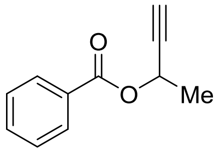

Synthesized according to *General Procedure 1* using benzoyl chloride (1.74 ml, 15 mmol, 1.2 equiv.). Yield: 1.39 g, 8.00 mmol, 64%. Spectroscopic data agrees with literature known values.[7] **^{1}H NMR** (400 MHz, $CDCl_3$, 298 K) δ/ppm: 8.09 – 8.05 (m, 2H, Ar-H), 7.60 – 7.55 (m, 1H, Ar-H), 7.48 – 7.41 (m, 2H, Ar-H), 5.69 (qd, $^3J_{HH}$ = 6.7 Hz, $^4J_{HH}$ = 2.1 Hz, 1H), 2.49 (d, $^4J_{HH}$ = 2.1 Hz, 1H), 1.65 (d, $^3J_{HH}$ = 6.7 Hz, 3H). **^{13}C NMR** (101 MHz, $CDCl_3$, 298 K) δ/ppm: 165.6 (s), 133.3 (s), 129.9 (s), 128.5 (s), 82.3 (s), 73.2 (s), 60.8 (s), 31.1 (s), 21.5 (s).

1.3 Synthesis of products.

General Procedure 2: Terminal alkyne **1** (0.2 mmol, 1 equiv.) was added to $PhBCl_2$ (32 mg, 0.2 mmol, 1 equiv.) in $CDCl_3$ (0.5 ml) in an NMR tube at ambient temperature, with the progress being monitored *via in situ* multinuclear NMR spectroscopy.

General Procedure 3: Terminal alkyne **2** (0.2 mmol, 1 equiv.) was added to $PhBCl_2$ (32 mg, 0.2 mmol, 1 equiv.) in $CDCl_3$ (0.5 ml) in an NMR tube, with the progress being monitored *via in situ* multinuclear NMR spectroscopy. After 8 h at 45 °C, the solvents were removed *in vacuo* with the residue being washed with cold hexane (3 × 1 ml). This was then dissolved in a CH_2Cl_2/hexane solution and stored at -40 °C overnight to yield a crop of white crystals suitable for X-ray diffraction. The solid was isolated and dried to yield the pure product as a white solid. Unfortunately, repeated attempts to obtain mass spectrometry or elemental analysis data was unsuccessful presumably due to instability of compounds **3** and **4**. However, full multinuclear NMR spectrocopic characterization was possible including HSQC and HMBC to fully assign the products.

Synthesis of (E)-3-chloro-1-(chloro(phenyl)boryl)prop-1-en-2-yl benzoate (**3a**).

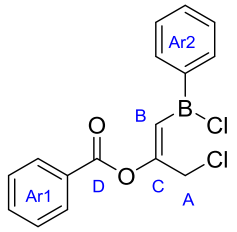

Compound **3a** was synthesized according to *General Procedure 2* after 8 h using **1a** (32 mg, 0.2 mmol). Conversion: >95%. **^{1}H NMR** (400 MHz, $CDCl_3$, 298 K) δ/ppm: 8.09 – 8.00 (m, 3H, Ar1), 7.59 – 7.51 (m, 2H, Ar1), 7.46 – 7.36 (m, 5H, Ar2), 6.44 (s, 1H, H^B), 4.55 (s, 2H, H^A). **^{13}C NMR** (101 MHz, $CDCl_3$, 298 K) δ/ppm: 164.3 (s, C^D), 160.0 (s, C^c), 137.2 (s, Ar2), 136.6 (s, Ar1), 134.7 (s, Ar1), 134.1 (s, Ar1), 130.4 (s, Ar1), 128.8 (s, Ar2), 128.4 (s, Ar2), 128.0 (s, Ar2), 119.1 (br. s, C^B), 41.8 (s, C^A). **^{11}B NMR** (128 MHz, $CDCl_3$, 298 K) δ/ppm: 59.5 (br. s).

Synthesis of (E)-3-chloro-1-(chloro(phenyl)boryl)prop-1-en-2-yl 4-fluorobenzoate (**3b**).

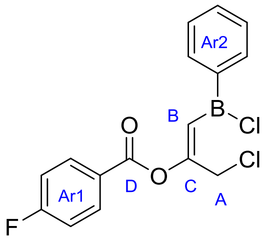

Compound **3b** was synthesized according to *General Procedure 2* after 18 h using **1b** (36 mg, 0.2 mmol). Conversion: >95%. **^{1}H NMR** (400 MHz, $CDCl_3$, 298 K) δ/ppm: 8.11 – 8.06 (m, 2H, Ar1), 8.02 – 7.99 (m, 2H, Ar2), 7.55 – 7.53 (m, 1H, Ar2), 7.41 – 7.36 (m, 2H, Ar2), 7.11 – 7.06 (m, 2H, Ar1), 6.42 (s, 1H, H^B), 4.53 (s, 2H, H^A). **^{13}C NMR** (101 MHz, $CDCl_3$, 298 K) δ/ppm: 166.5 (d, $^1J_{CF}$ = 255.9 Hz, Ar1), 163.3 (s, C^D), 159.7 (s, C^C), 136.9 (s, Ar2), 136.6 (s, Ar2), 134.7 (s, Ar2), 133.1 (d, $^3J_{CF}$ = 9.6 Hz, Ar1), 128.4 (s, Ar2), 125.3 (d, $^4J_{CF}$ = 3.0 Hz, Ar1), 119.1 (br. s, C^B), 116.1 (d, $^2J_{CF}$ = 22.1 Hz, Ar1), 41.8 (s, C^A). **^{19}F NMR** (376 MHz, $CDCl_3$, 298 K) δ/ppm: -103.5 (s). **^{11}B NMR** (128 MHz, $CDCl_3$, 298 K) δ/ppm: 59.3 (br. s).

Synthesis of (E)-3-chloro-1-(chloro(phenyl)boryl)prop-1-en-2-yl 4-nitrobenzoate (**3c**).

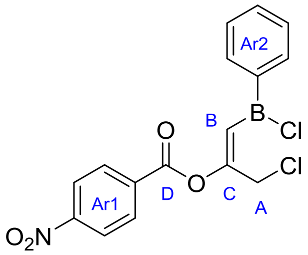

Compound **3c** was synthesized according to *General Procedure 2* after 18 h using **1c** (41 mg, 0.2 mmol). Conversion: >95%. **^{1}H NMR** (400 MHz, $CDCl_3$, 298 K) δ/ppm: 8.27 – 8.25 (m, 4H, Ar1), 8.12 – 8.10 (m, Hz, 2H, Ar2), 7.67 – 7.63 (m, 1H, Ar2), 7.53 – 7.48 (m, 2H, Ar2), 6.55 (s, 1H, H^B), 4.63 (s, 2H, H^A). **^{13}C NMR** (101 MHz, $CDCl_3$, 298 K) δ/ppm: 162.4 (s, C^D), 158.7 (s, C^C), 151.1 (s, Ar1), 136.6 (s, Ar2), 134.9 (s, Ar2), 134.4 (s, Ar1), 131.5 (s, Ar1), 128.4 (s, Ar2), 123.9 (s, Ar1), 119.6 (br. s, C^B), 41.6 (s, C^A). **^{11}B NMR** (128 MHz, $CDCl_3$, 298 K) δ/ppm: 59.9 (br. s).

Synthesis of (E)-3-chloro-1-(chloro(phenyl)boryl)prop-1-en-2-yl 3-nitrobenzoate (**3d**).

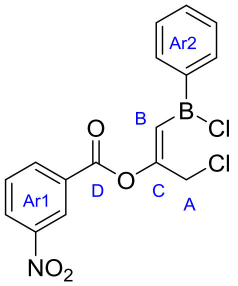

Compound **3d** was synthesized according to *General Procedure 2* after 48 h using **1d** (41 mg, 0.2 mmol). Conversion >95%. **^{1}H NMR** (400 MHz, $CDCl_3$, 298 K) δ/ppm: 9.03 – 8.94 (m, 1H, Ar1), 8.52 – 8.48 (m, 2H, Ar1), 8.14 – 8.10 (m, 2H, Ar2), 7.77 – 7.73 (m, 1H, Ar1), 7.68 – 7.63 (m, 1H, Ar2), 7.54 – 7.49 (m, 2H, Ar2), 6.55 (s, 1H, H^B), 4.65 (s, 2H, H^A). **^{13}C NMR** (101 MHz, $CDCl_3$, 298 K) δ/ppm: 162.2 (s, C^D), 158.8 (s, C^C), 148.5 (s, Ar1), 137.0 (s, Ar2), 136.7 (s, Ar2),

135.9 (s, Ar1), 134.9 (s, Ar2), 130.8 (s, Ar1), 130.2 (s, Ar1), 128.5 (s, Ar2), 128.3 (s, Ar1), 125.3 (s, Ar1), 119.7 (br. s, C^B), 41.6 (s, C^A). **^{11}B NMR** (128 MHz, $CDCl_3$, 298 K) δ/ppm: 60.0 (br. s).

Synthesis of 2,3-dichloro-2,6-diphenyl-4-(propan-2-ylidene)-3,4-dihydro-2H-1,5,2-dioxaborinin-1-ium-2-uide (**4a**).

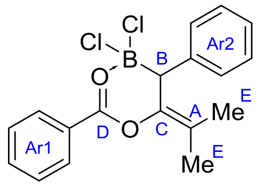

Compound **4a** was synthesized according to *General Procedure 3* using **2a** (37 mg, 0.2 mmol, 1 equiv.). Yield: 57 mg, 0.16 mmol, 82%. **^{1}H NMR** (400 MHz, $CDCl_3$, 298 K) δ/ppm: 8.22 (d, $^3J_{HH}$ = 7.5 Hz. 2H, Ar1), 7.78 (t, $^3J_{HH}$ = 7.5 Hz, 1H, Ar1), 7.55 (t, $^3J_{HH}$ = 7.9 Hz, 2H, Ar1), 7.12 (t, $^3J_{HH}$ = 7.5, 2H, Ar2), 7.05 (t, $^3J_{HH}$ = 7.3 Hz, 1H, Ar2), 6.97 (d, $^3J_{HH}$ = 7.4 Hz, 2H, Ar2), 3.82 (s, 1H, H^B), 1.95 (s, 3H, H^E), 1.77 (s, 3H, H^E). **^{13}C NMR** (101 MHz, $CDCl_3$, 298 K) δ/ppm: 171.4 (s, C^D), 147.8 (s, C^C), 141.1 (s, Ar2), 138.1 (s, Ar1), 131.6 (s, Ar1), 129.8 (s, Ar1), 128.4 (s, Ar2), 127.5 (s, Ar2), 125.9 (s, Ar2), 124.7 (s, Ar1), 122.5 (s, C^A), 39.51 (br. s, C^B), 18.7 (s, C^E), 17.7 (s, C^E). **^{11}B NMR** (128 MHz, $CDCl_3$, 298 K) δ/ppm: 8.7 (s). **IR** ν_{max} (cm^{-1}): 3201 (w), 2993 (w), 2916 (w). 1689 (m), 1597 (m), 1535 (s), 1496 (m), 1450 (m), 1404 (s), 1311 (m), 1242 (m), 1180 (s), 1056 (m), 1026 (w), 987 (w), 933 (w), 879 (w), 833 (w), 802 (m), 740 (s), 694 (s).

Synthesis of 2,2-dichloro-3-phenyl-4-(propan-2-ylidene)-6-(4-(p-tolyl))-3,4-dihydro-2H-1,5,2-dioxaborinin-1-ium-2-uide (**4b**).

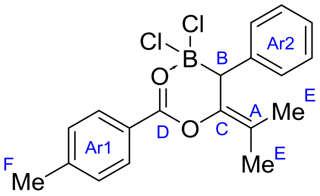

Compound **4b** was synthesized according to *General Procedure 3* using **2b** (40 mg, 0.2 mmol, 1 equiv.). Yield: 67 mg, 0.17 mmol, 87%. **^{1}H NMR** (400 MHz, $CDCl_3$, 298 K) δ/ppm: 8.20 (d, $^3J_{HH}$ = 8.3 Hz, 2H, Ar1), 7.44 (d, $^3J_{HH}$ = 8.1 Hz, 2H, Ar1), 7.21 (t, $^3J_{HH}$ = 7.5 Hz, 2H, Ar2), 7.14 (t, $^3J_{HH}$ = 7.3 Hz, 1H, Ar2), 7.06 (d, $^3J_{HH}$ = 7.4 Hz, 2H, Ar2), 3.90 (s, 1H, H^B), 2.53 (s, 3H, H^F), 2.03 (s, 3H, H^E), 1.86 (s, 3H, H^E). **^{13}C NMR** (101 MHz, $CDCl_3$, 298 K) δ/ppm: 171.3 (s, C^D), 150.4 (s, Ar1), 147.6 (s, C^C), 141.2 (s, Ar2), 131.7 (s, Ar1), 130.6 (s, Ar1), 128.4 (s, Ar2), 127.5 (s, Ar2), 125.9 (s, Ar2), 122.0 (s, C^A), 121.8 (s, Ar1), 39.6 (br. s, C^B), 22.5 (s, C^F), 18.6 (br. s, C^E), 17.7 (s, C^E). **^{11}B NMR** (128 MHz, $CDCl_3$, 298 K) δ/ppm: 8.6 (s). **IR** ν_{max} (cm^{-1}): 2160 (w), 1666 (w), 1605 (w), 1581 (w), 1527 (s), 1504 (m), 1412 (s), 1296 (m), 1249 (m), 1172 (m), 1134 (m), 1080 (m), 1049 (m), 972 (w), 933 (w), 910 (w), 879 (w), 833 (m), 763 (s), 740 (s), 648 (m).

Synthesis of 2,2-dichloro-6-(4-fluorophenyl)-3-phenyl-4-(propan-2-ylidene)-3,4-dihydro-2H-1,5,2-dioxaborinin-1-ium-2-uide (**4c**).

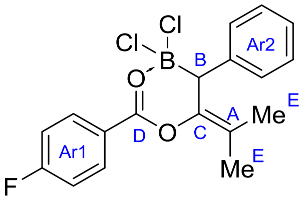

Compound **4c** was synthesized according to *General Procedure 3* using **2c** (41 mg, 0.2 mmol, 1 equiv.). Yield: 58 mg, 16 mmol,79%. **^{1}H NMR** (400 MHz, $CDCl_3$, 298 K) δ/ppm: 8.37 – 8.34 (m, 2H, Ar1), 7.33 (t, $^3J_{HF}$ = 8.5 Hz, 2H, Ar1), 7.22 (t, $^3J_{HH}$ = 7.4 Hz, 2H, Ar2), 7.15 (t, $^3J_{HH}$ = 7.3 Hz, 1H,

Ar2), 7.05 (d, $^3J_{HH}$ = 7.3 Hz, 2H, Ar2), 3.91 (s, 1H, H^B), 2.03 (s, 3H, H^E), 1.86 (s, 3H, H^E). **^{13}C NMR** (101 MHz, $CDCl_3$, 298 K) δ/ppm: 170.3, (s, C^D), 168.9 (d, $^1J_{CF}$ = 264.1 Hz, Ar1), 147.9 (s, C^C), 140.9 (s, Ar2), 134.8 (d, $^3J_{CF}$ = 10.6 Hz, Ar1), 128.5 (s, Ar2), 127.5 (s, Ar2), 126.0 (s, Ar2), 122.6 (s, C^A), 121.0 (d, $^4J_{CF}$ = 2.7 Hz, Ar1), 117.6 (d, $^2J_{CF}$ = 22.6 Hz, Ar1), 39.6 (br. s, C^B), 18.7 (s, C^E), 17.7 (br. s, C^E). **^{11}B NMR** (128 MHz, $CDCl_3$, 298 K) δ/ppm: 8.8 (s). **^{19}F NMR** (471 MHz, $CDCl_3$, 298 K) δ/ppm: -94.6 – -94.5 (m, 1F, *p*-F). **IR** ν_{max} (cm^{-1}): 3201 (w), 3086 (w), 2916 (w), 1697 (w), 1597 (m), 1535 (m), 1504 (m), 1404 (s), 1311 (m), 1242 (s), 1157 (s), 1049 (m), 972 (w), 933 (w), 910 (w), 879 (w), 833 (m), 763 (s), 725 (s), 695 (s), 648 (m).

2 NMR spectra.

2.1 Mechanistic studies.

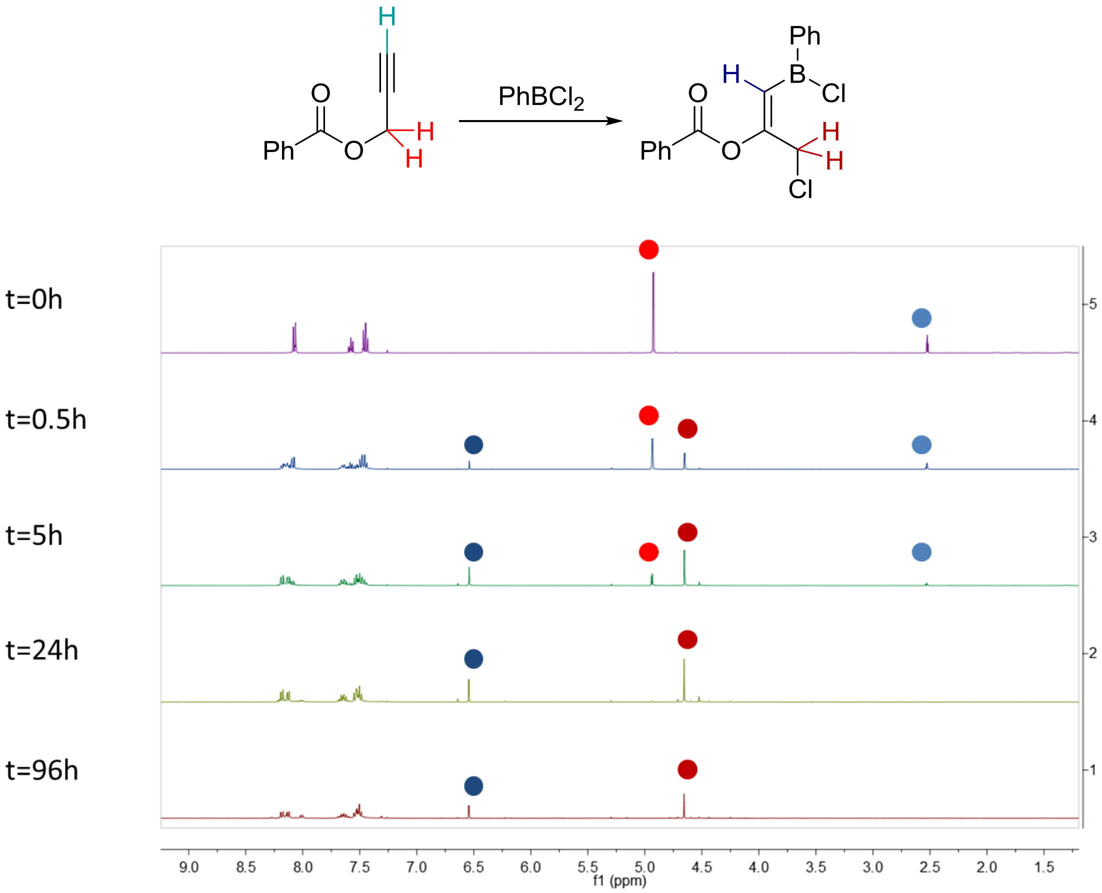

Figure S1: Stacked ^{1}H NMR spectra of the 1.3-haloboration of **1a** with $PhBCl_2$.

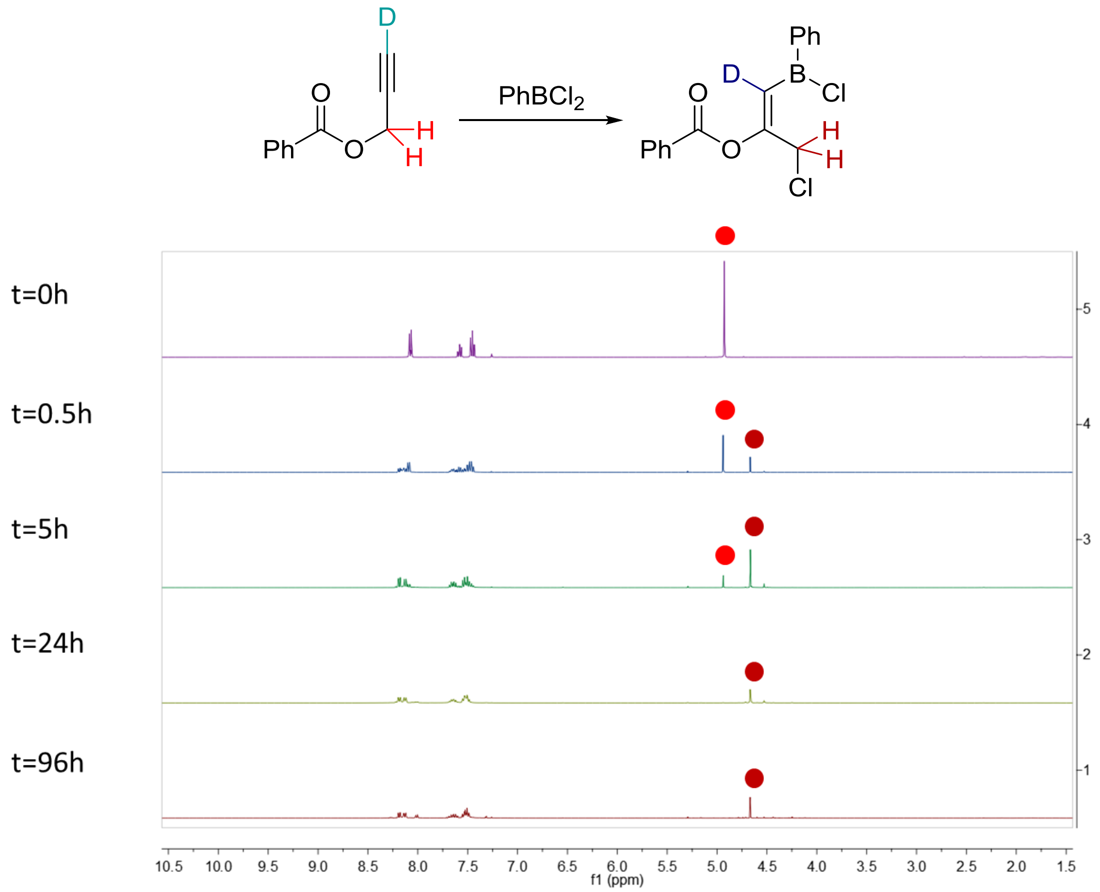

Figure S2: Stacked ^{1}H NMR spectra of the 1.3-haloboration of **1a^{D}** with $PhBCl_2$.

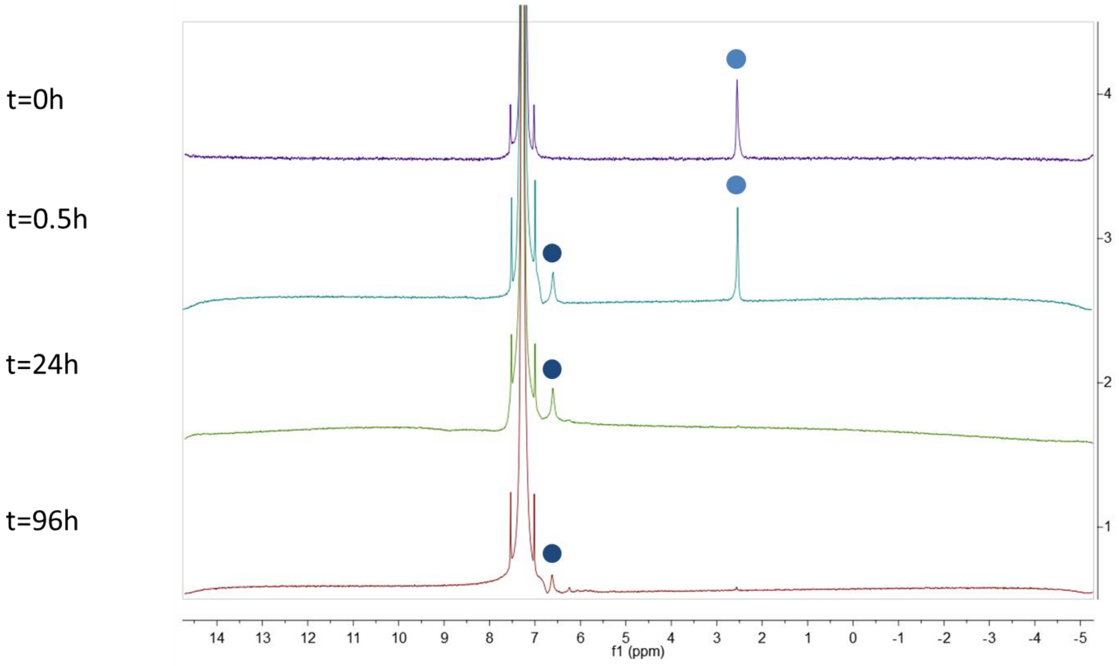

Figure S3: Stacked ^{2}H NMR spectra of the 1.3-haloboration of **1a^{D}** with $PhBCl_2$.

2.2 NMR spectra of starting materials.

Figure S4 ^{1}H NMR (400 MHz, $CDCl_3$, 298 K) spectrum of prop-2-yn-1-yl benzoate (**1a**).

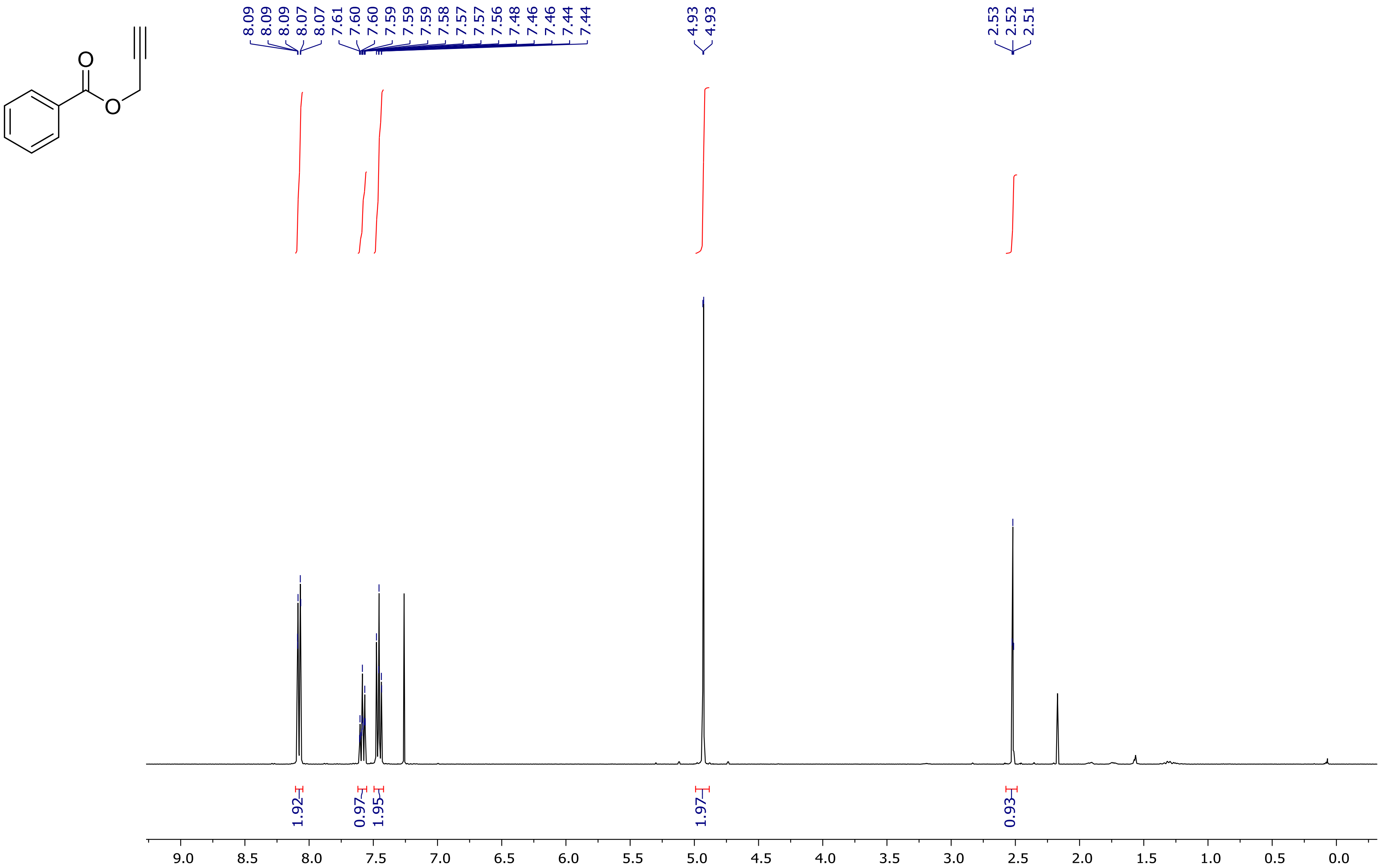

Figure S5 ^{13}C NMR (101 MHz, $CDCl_3$, 298 K) spectrum of prop-2-yn-1-yl benzoate (**1a**).

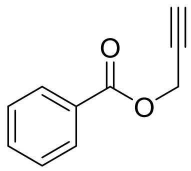

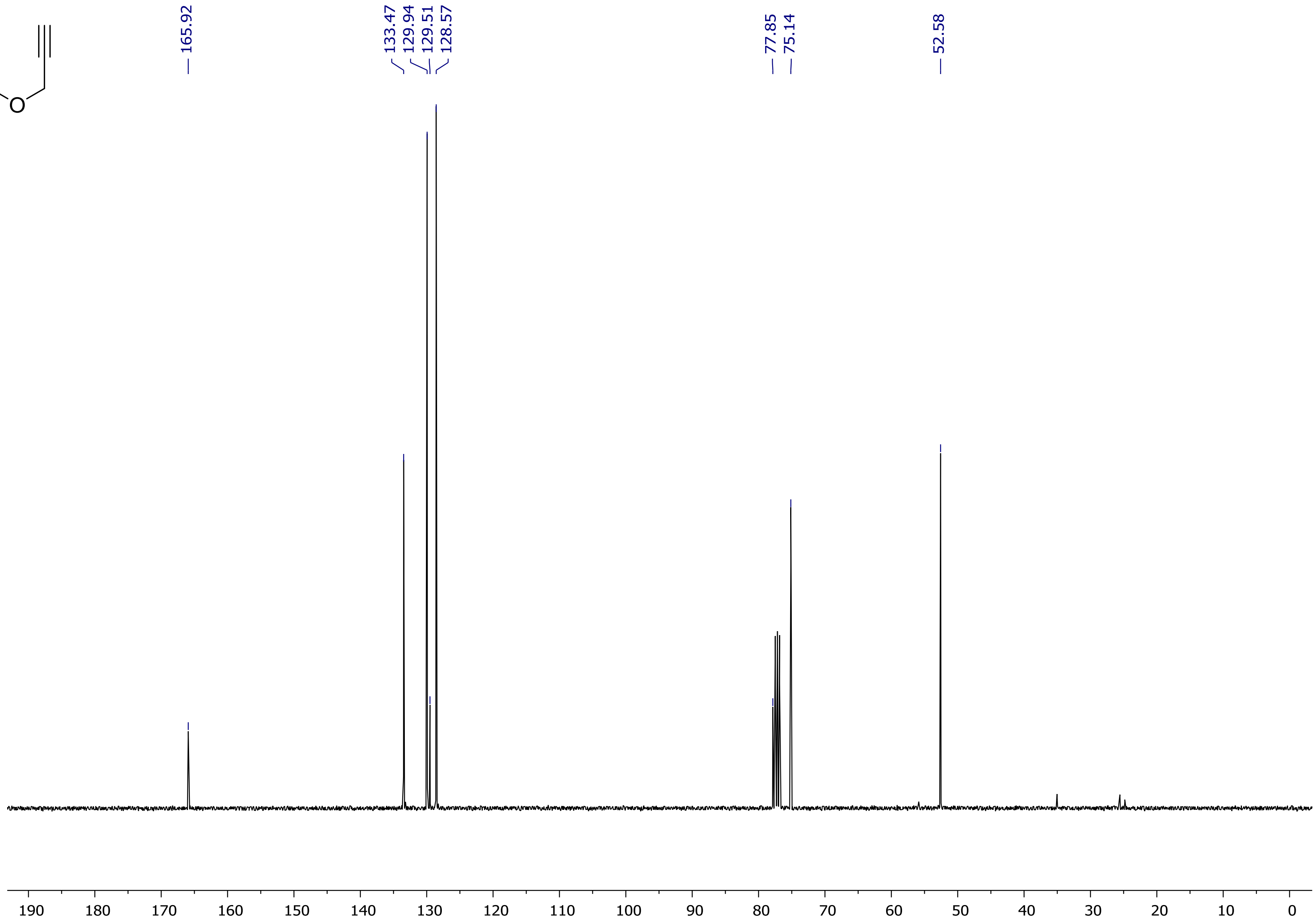

Figure S6 ^{1}H NMR (400 MHz, $CDCl_3$, 298 K) spectrum of prop-2-yn-1-yl 4-fluorobenzoate (**1b**).

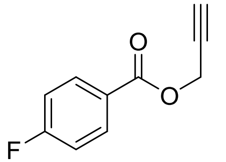

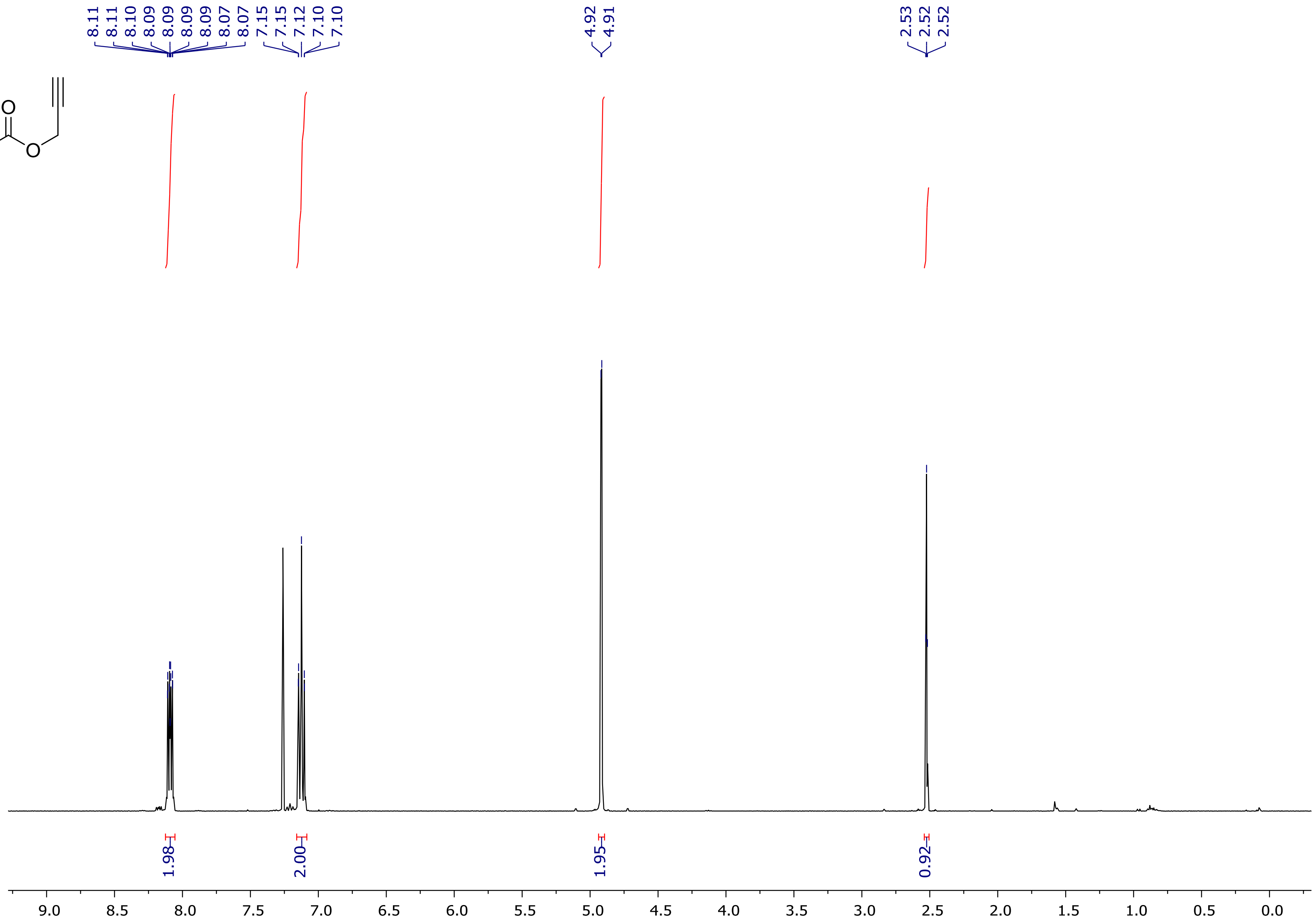

Figure S7 ^{13}C NMR (101 MHz, $CDCl_3$, 298 K) spectrum of prop-2-yn-1-yl 4-fluorobenzoate (**1b**).

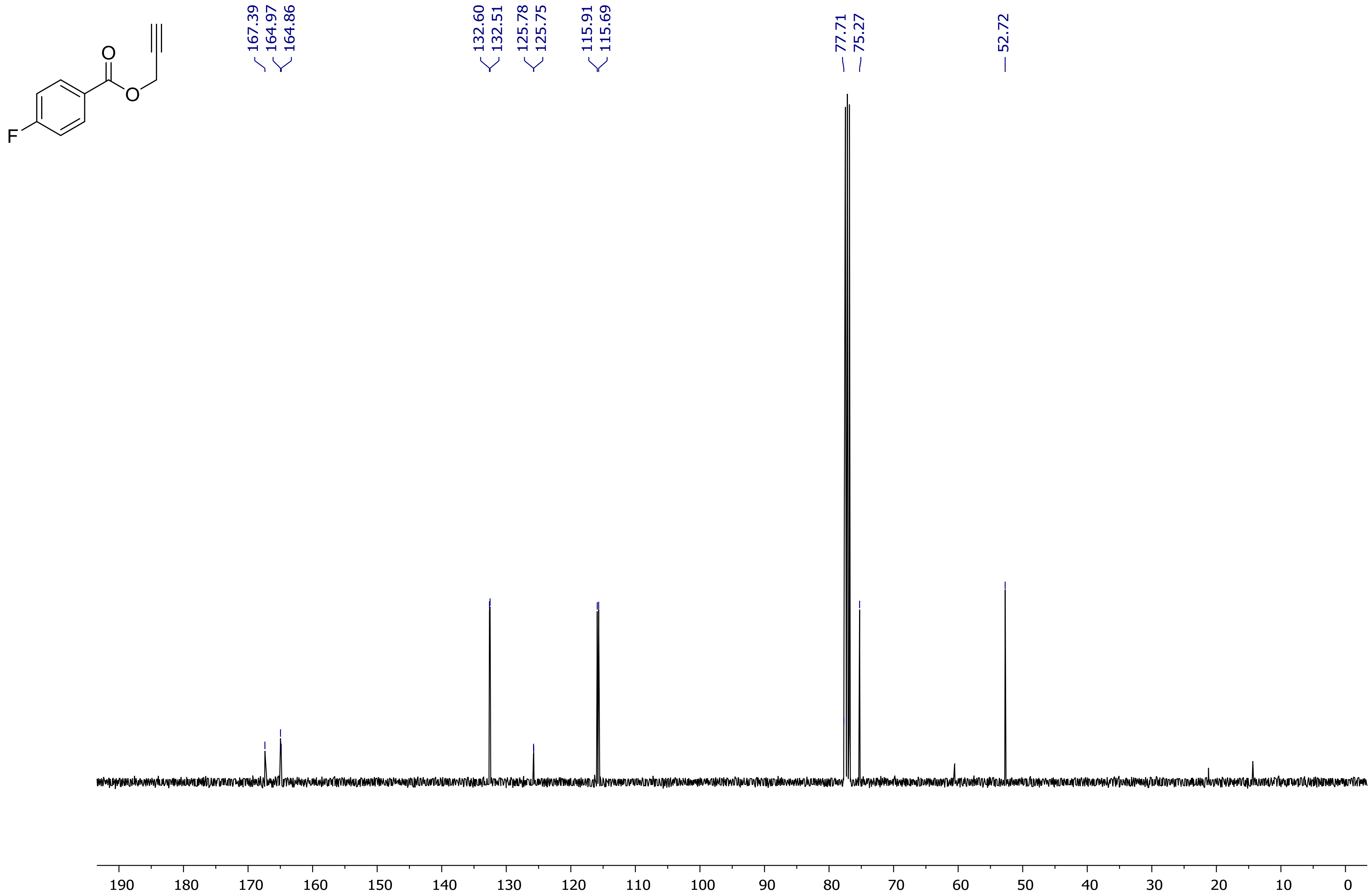

Figure S8 ^{19}F NMR (376 MHz, $CDCl_3$, 298 K) spectrum of prop-2-yn-1-yl 4-fluorobenzoate (**1b**).

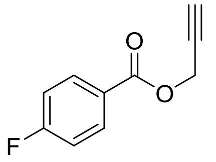

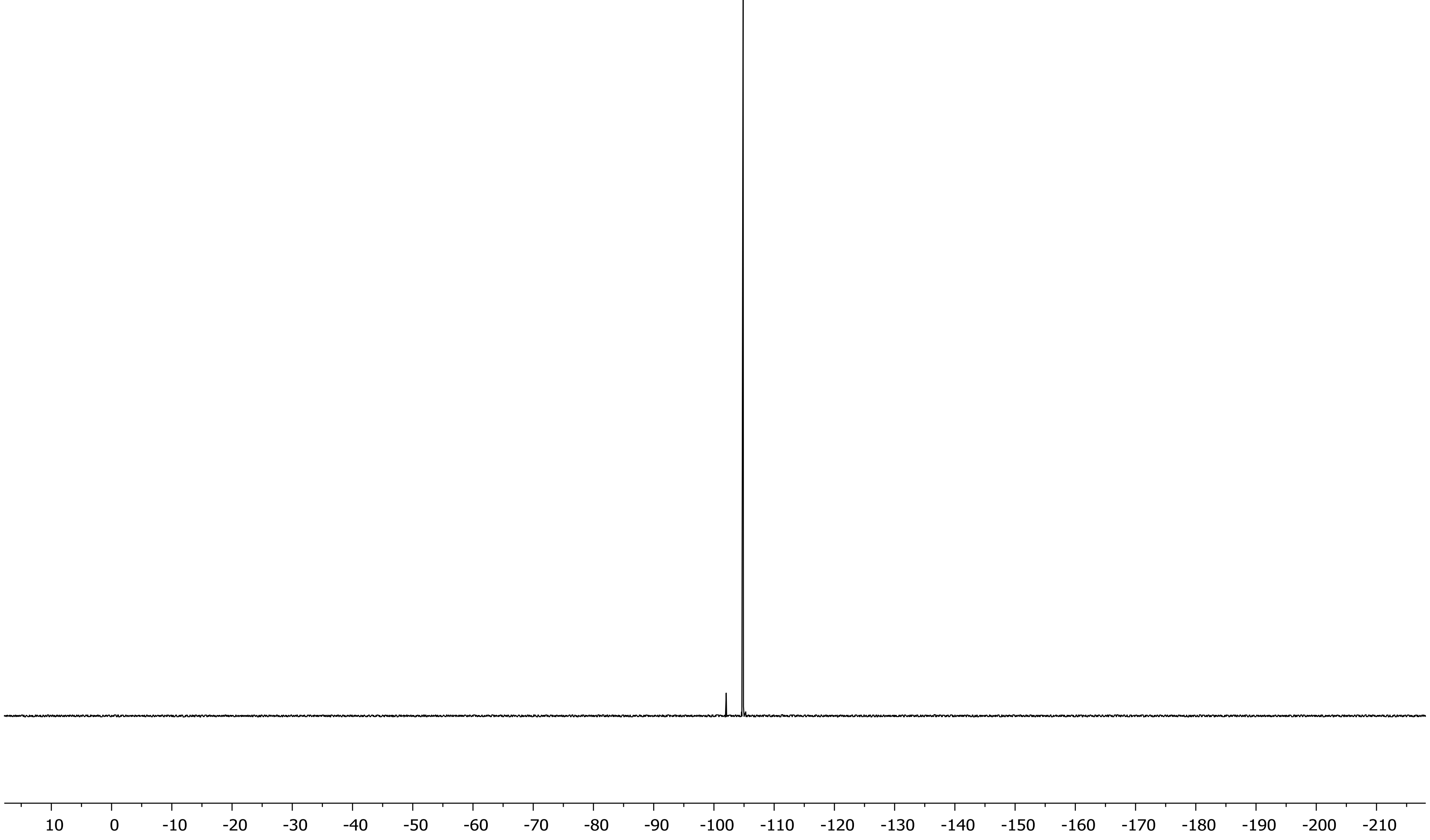

Figure S9 ^{1}H NMR (500 MHz, $CDCl_3$, 298 K) spectrum of prop-2-yn-1-yl 4-nitrobenzoate (**1c**).

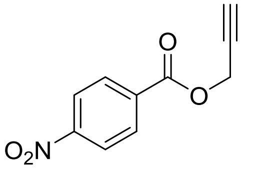

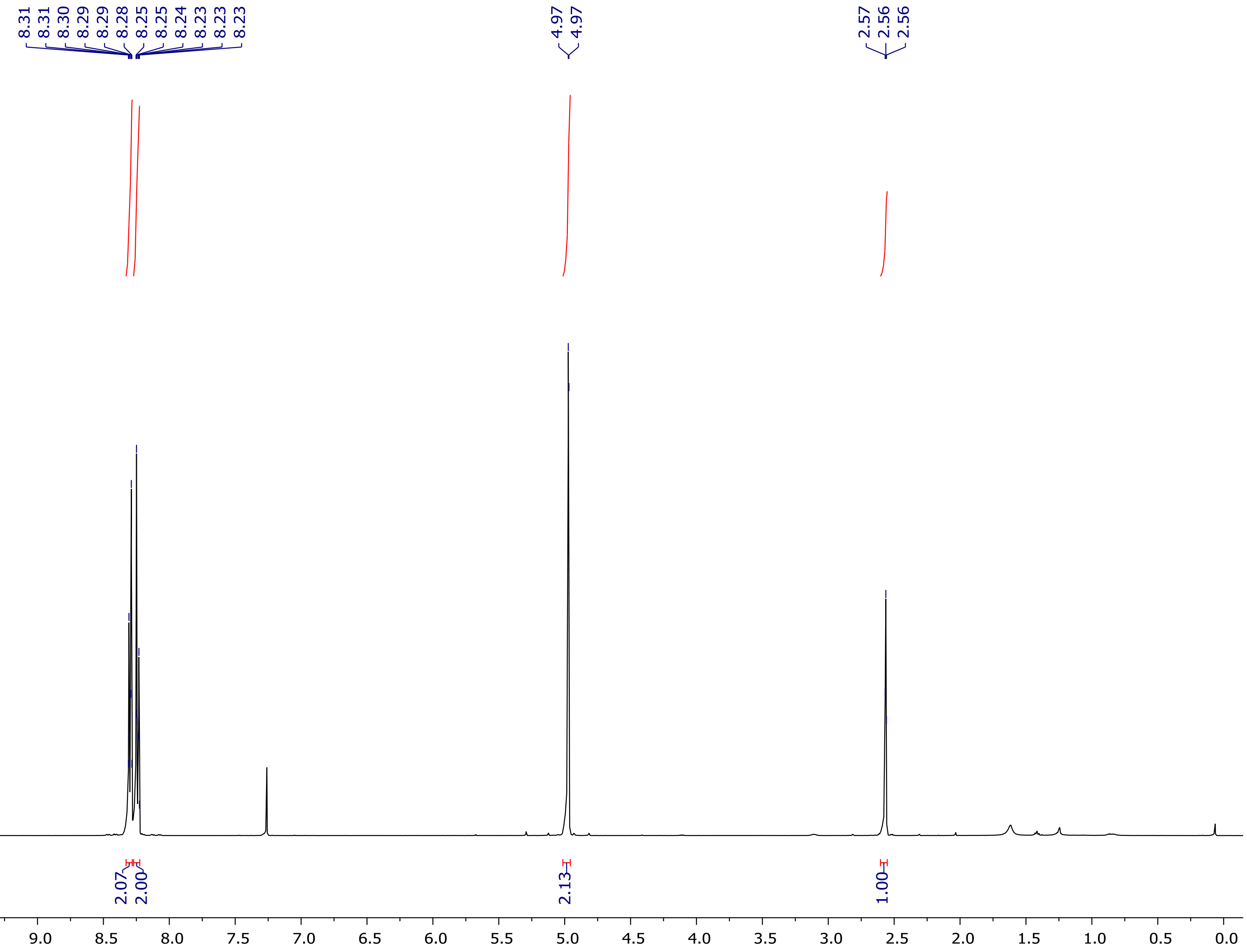

Figure S10 ^{13}C NMR (126 MHz, $CDCl_3$, 298 K) spectrum of prop-2-yn-1-yl 4-nitrobenzoate (**1c**).

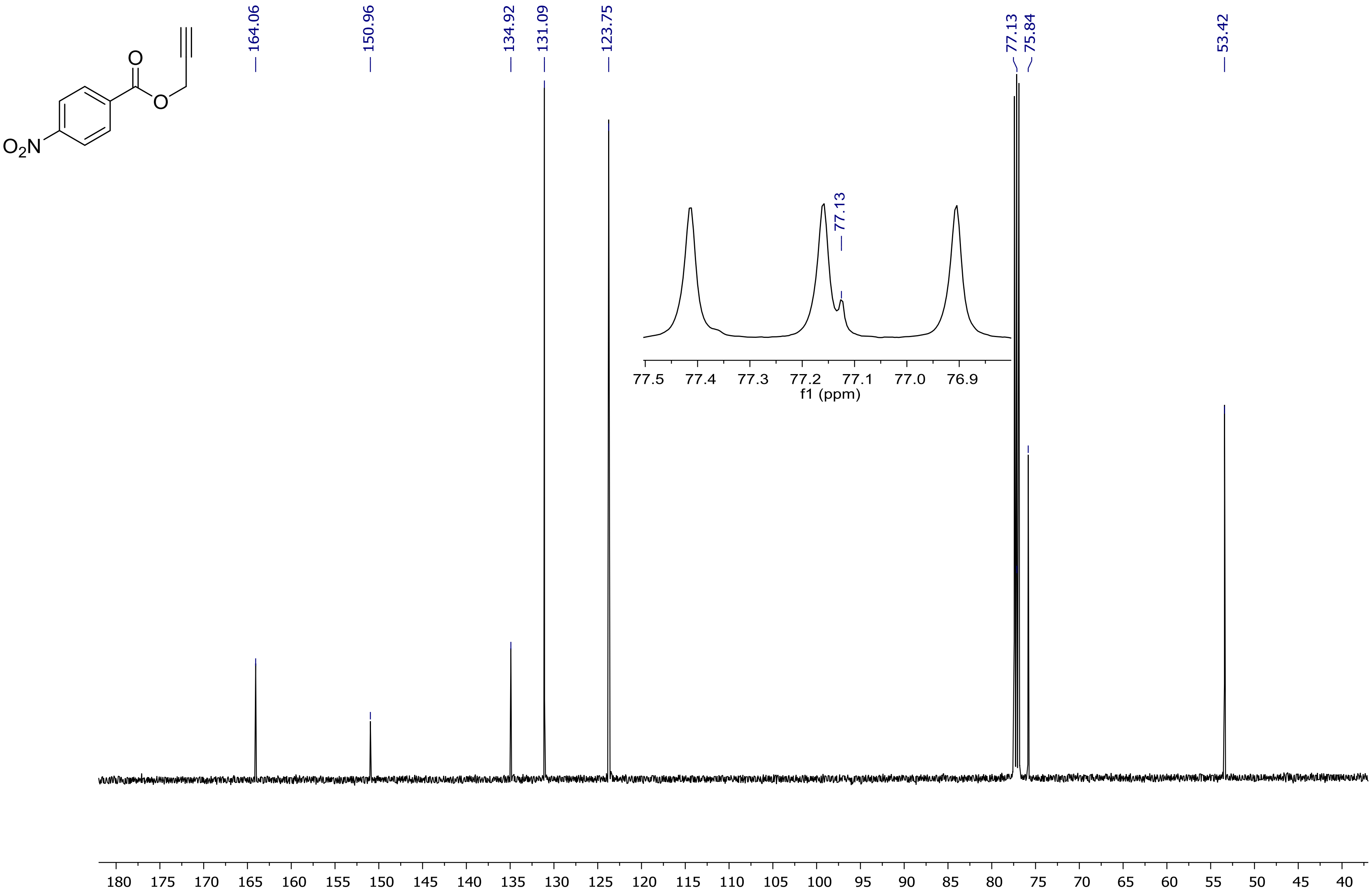

Figure S11 ^{1}H NMR (400 MHz, $CDCl_3$, 298 K) spectrum of prop-2-yn-1-yl 3-nitrobenzoate (**1d**).

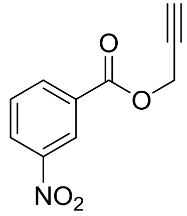

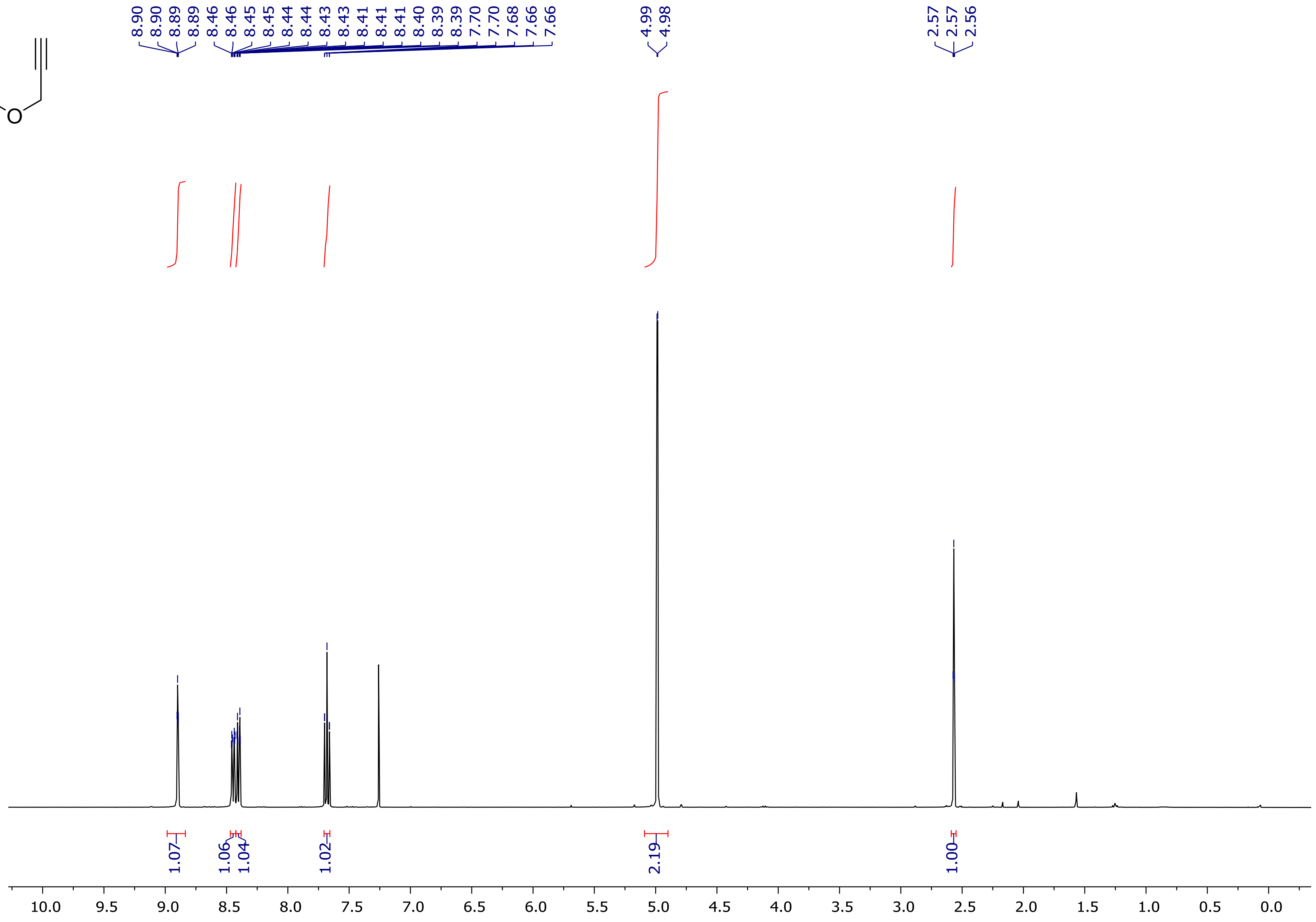

Figure S12 ^{13}C NMR (101 MHz, $CDCl_3$, 298 K) spectrum of prop-2-yn-1-yl 3-nitrobenzoate (**1d**).

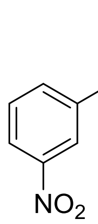

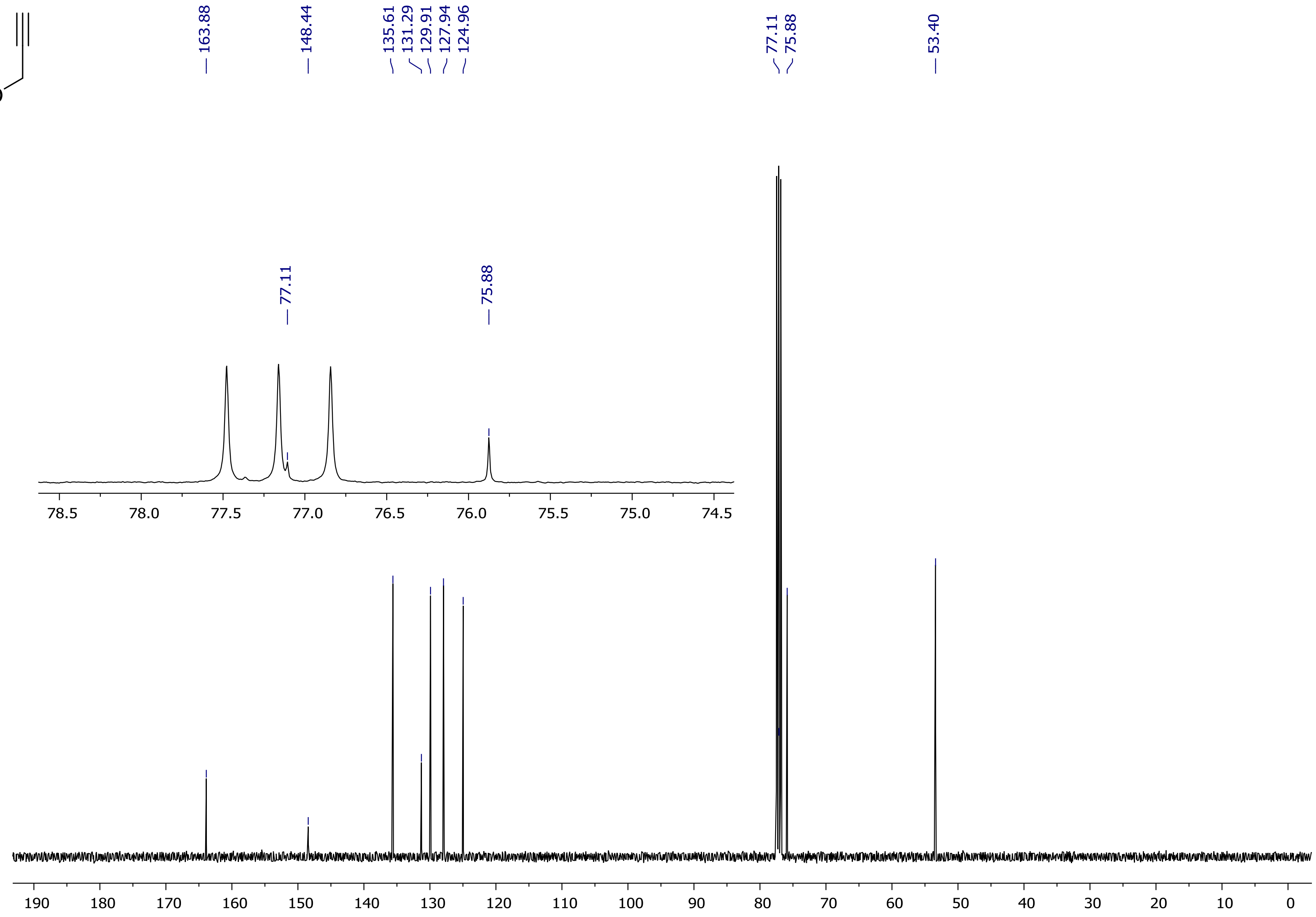

Figure S13 ^{1}H NMR (400 MHz, $CDCl_3$, 298 K) spectrum of 2-prop-3-yn-3-d-1-yl benzoate (**1a^D**).

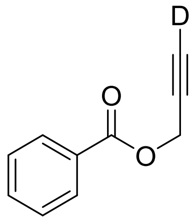

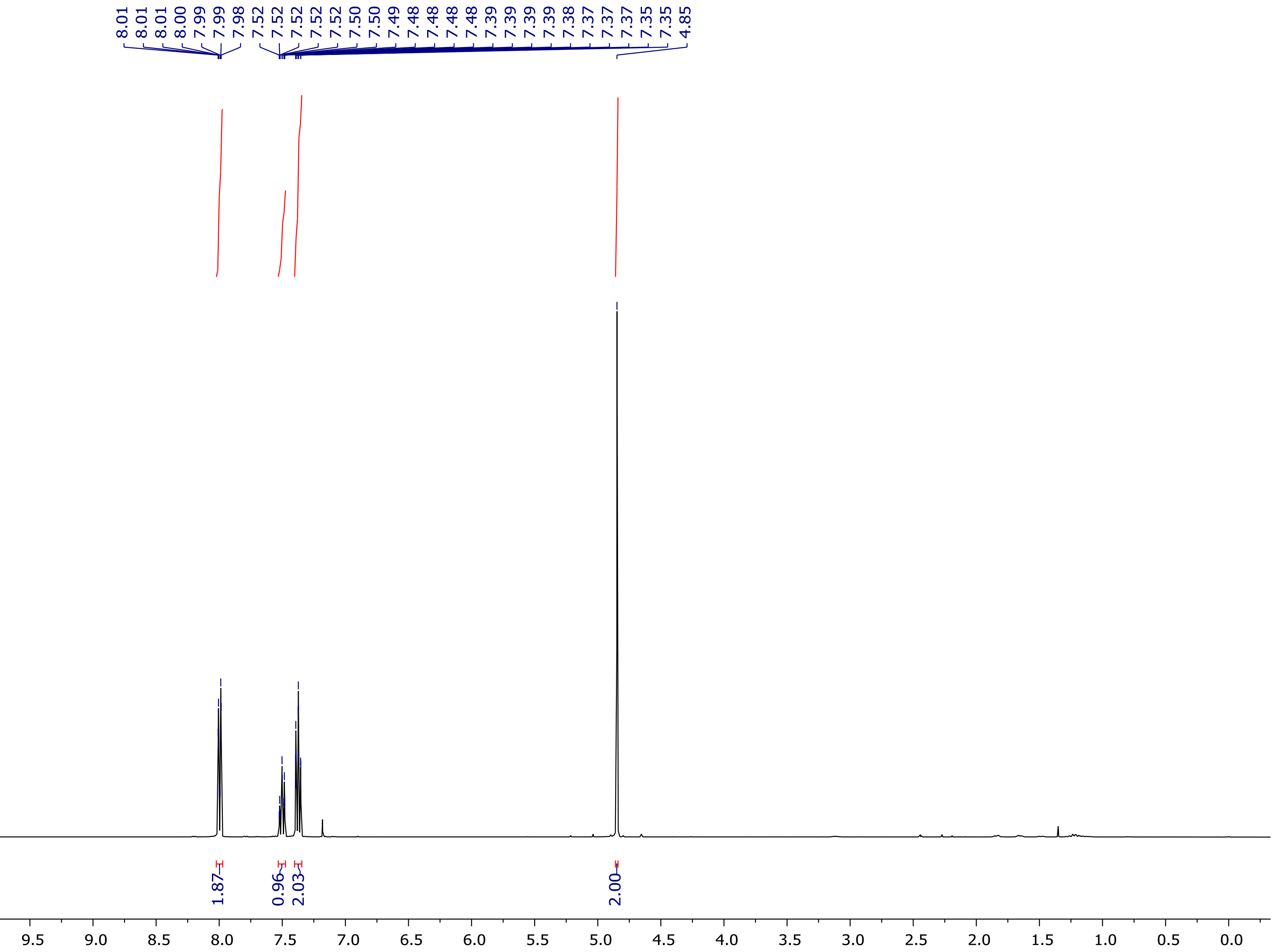

Figure S14 ^{2}H NMR (61 MHz, $CDCl_3$, 298 K) spectrum of benzoate of 2-prop-3-yn-3-d-1-yl benzoate (**1a^D**).

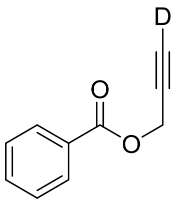

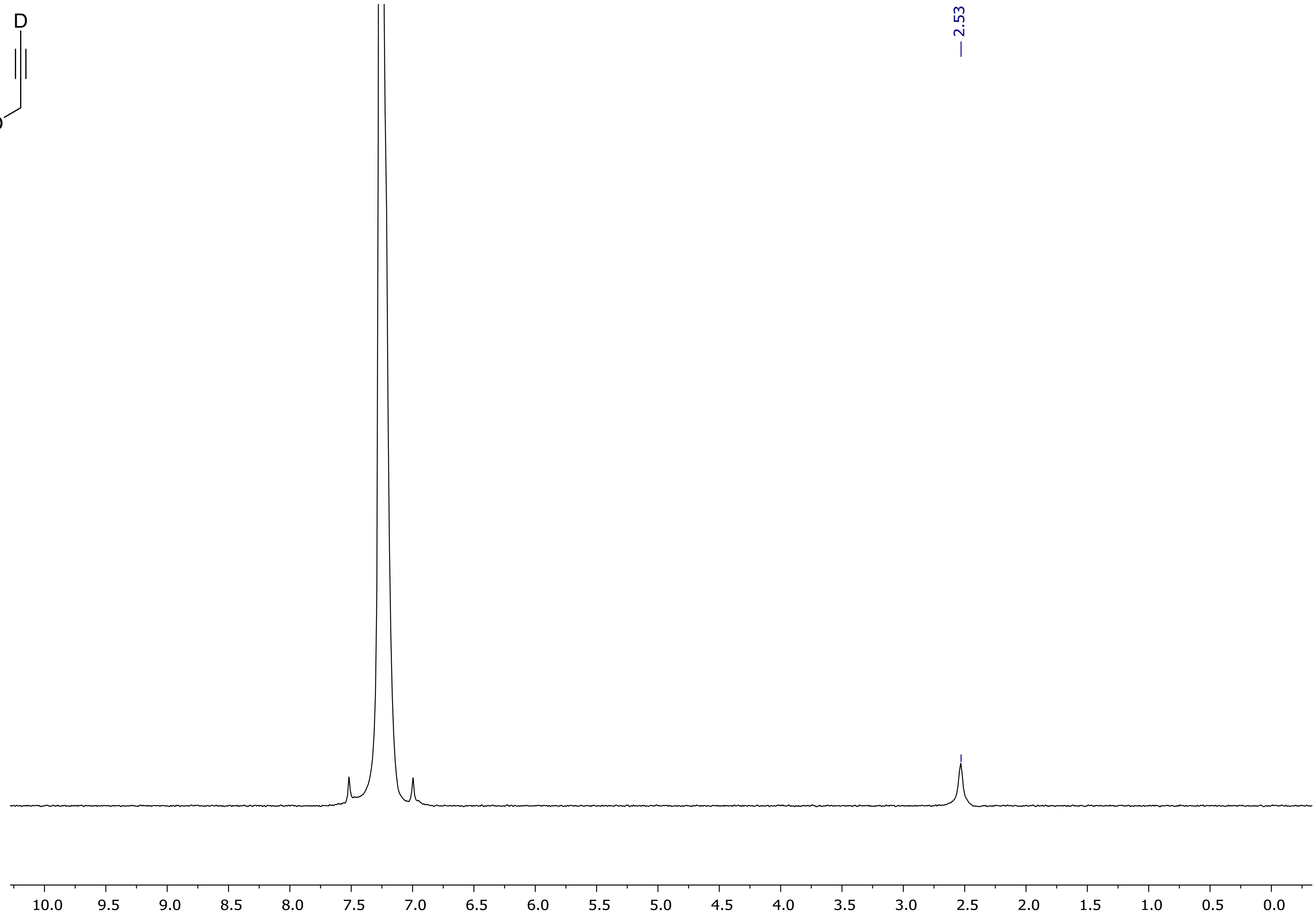

Figure S15 ^{13}C NMR (101 MHz, $CDCl_3$, 298 K) spectrum of 2-prop-3-yn-3-d-1-yl benzoate (**1a^{D}**).

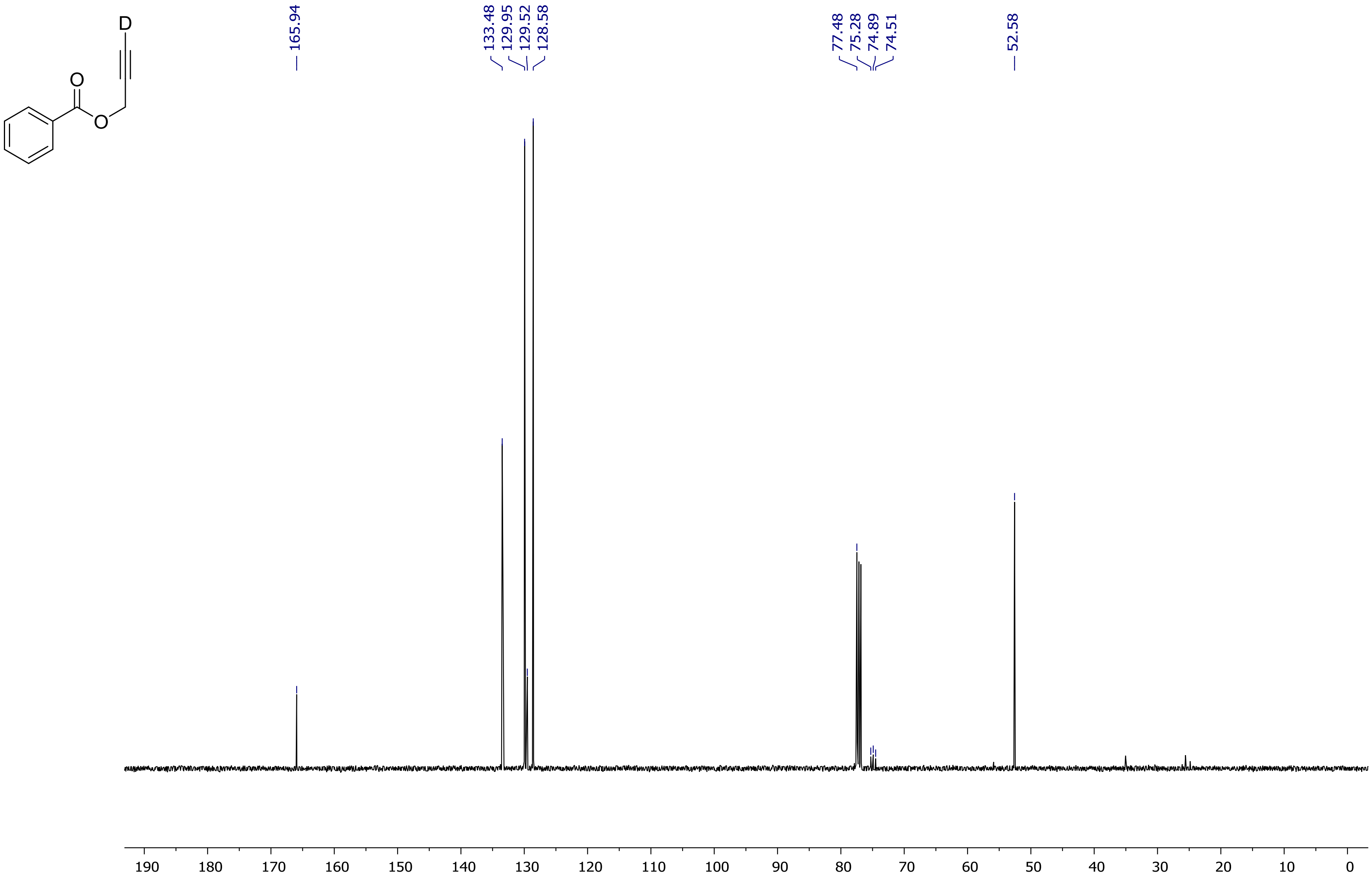

Figure S16 ^{1}H NMR (400 MHz, $CDCl_3$, 298 K) spectrum of 2-methylbut-3-yn-2-yl benzoate (**2a**).

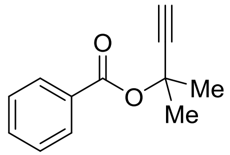

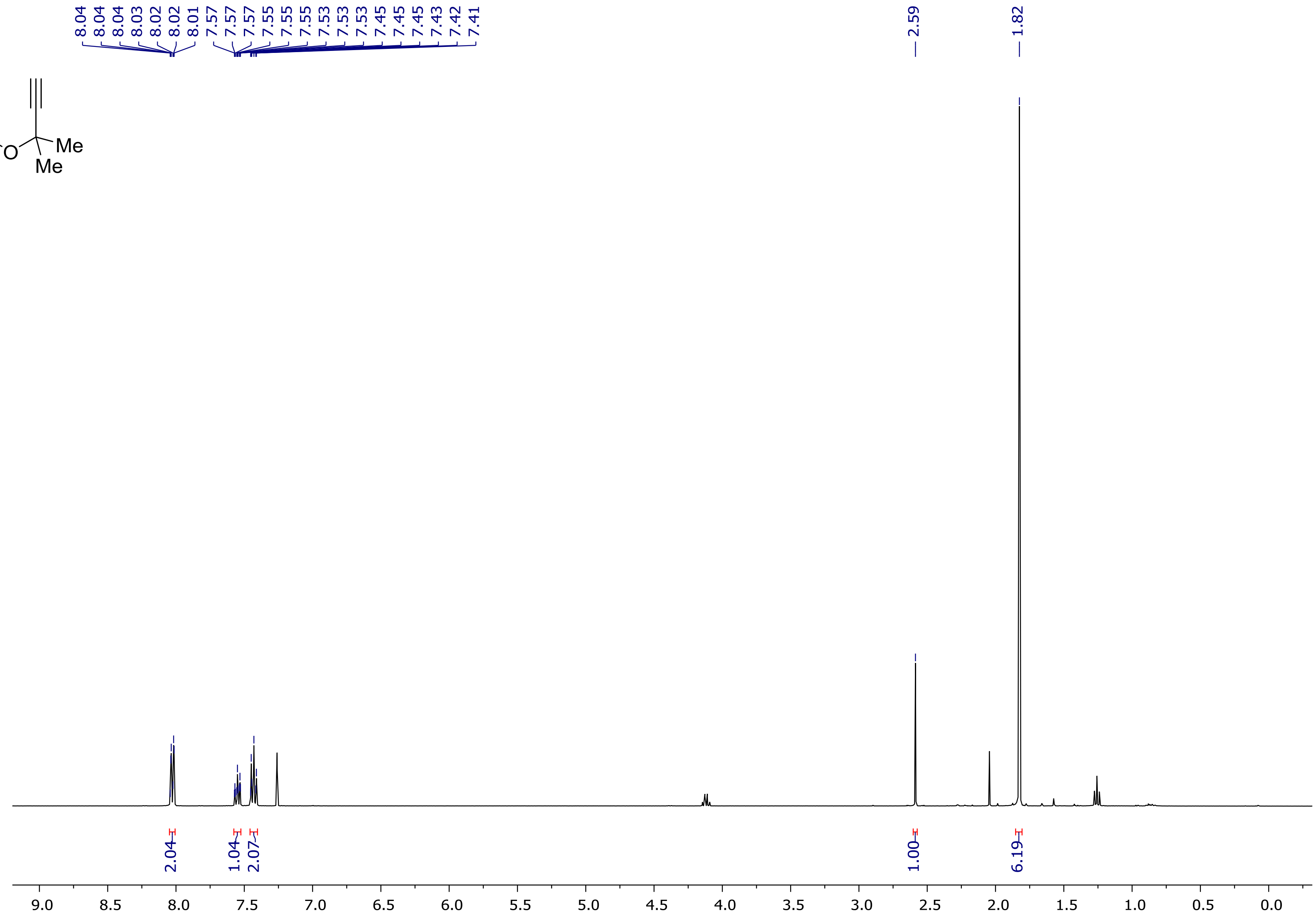

Figure S17 ^{13}C NMR (101 MHz, $CDCl_3$, 298 K) spectrum of 2-methylbut-3-yn-2-yl benzoate (**2a**).

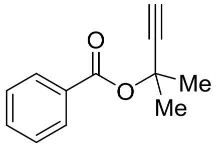

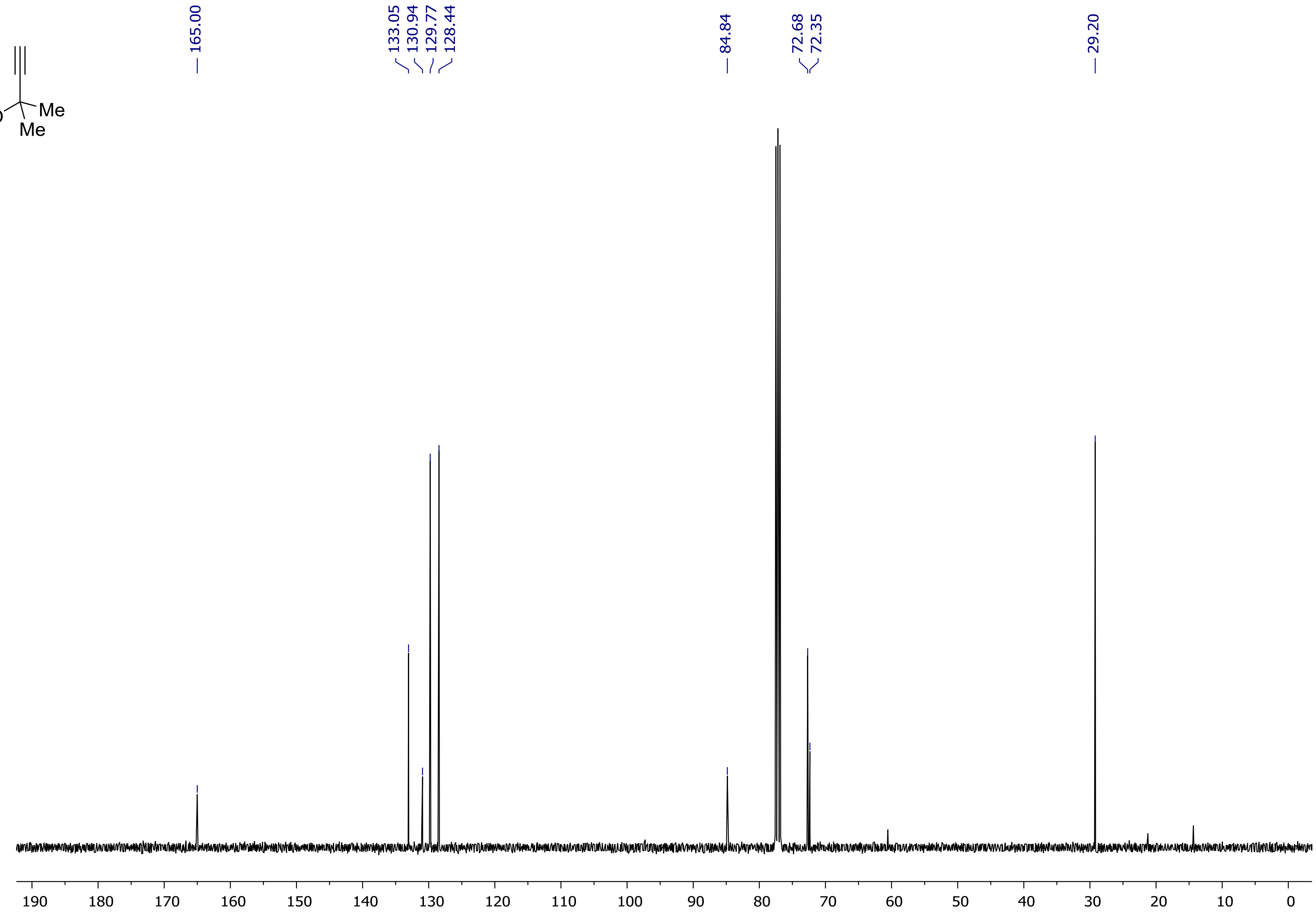

Figure S18 ^{1}H NMR (400 MHz, $CDCl_3$, 298 K) spectrum of 2-methylbut-3-yn-2-yl 4-methylbenzoate (**2b**).

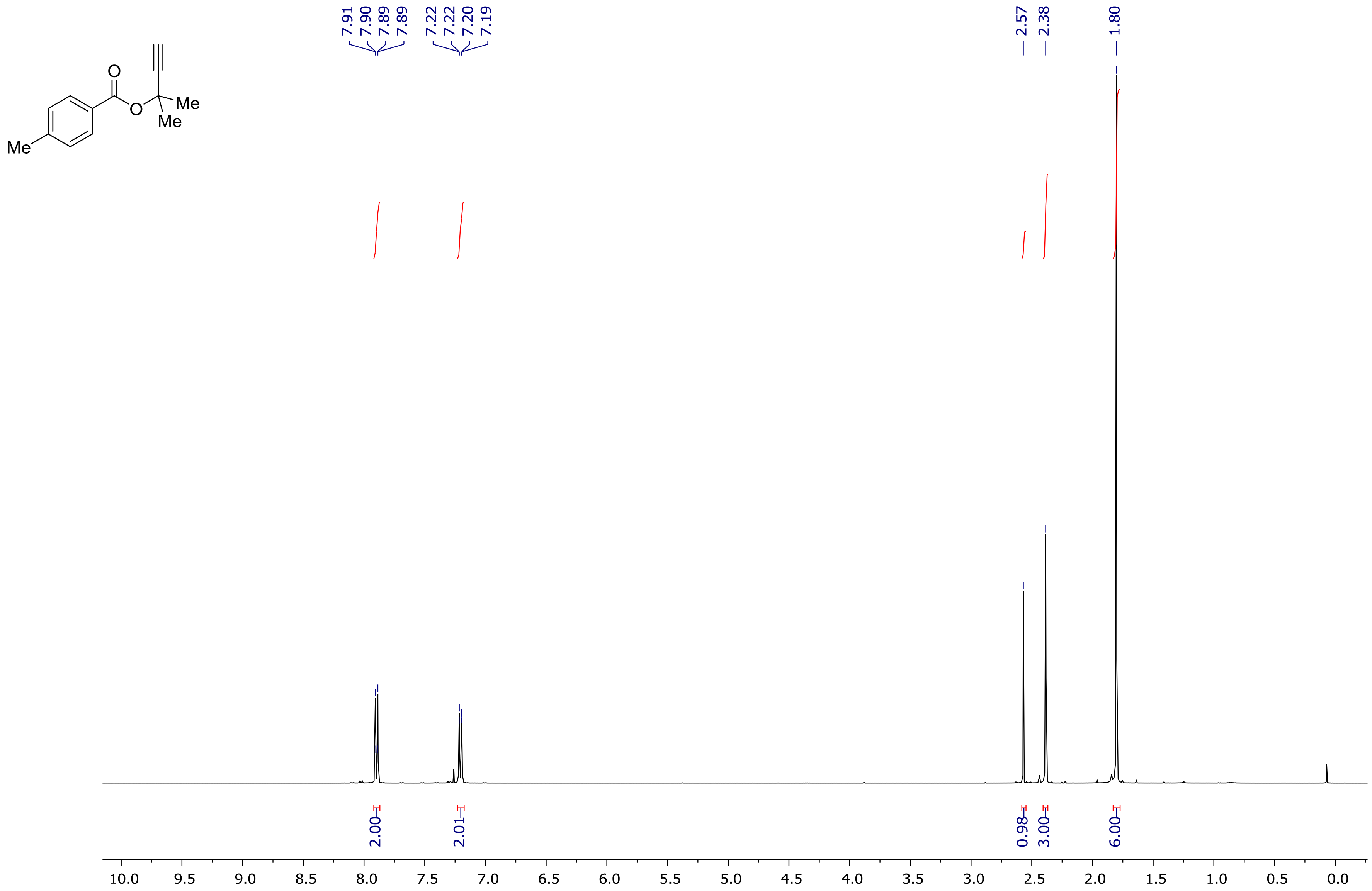

Figure S19 ^{13}C NMR (101 MHz, $CDCl_3$, 298 K) spectrum of 2-methylbut-3-yn-2-yl 4-methylbenzoate (**2b**).

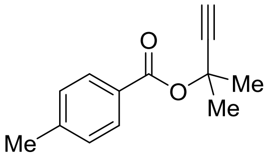

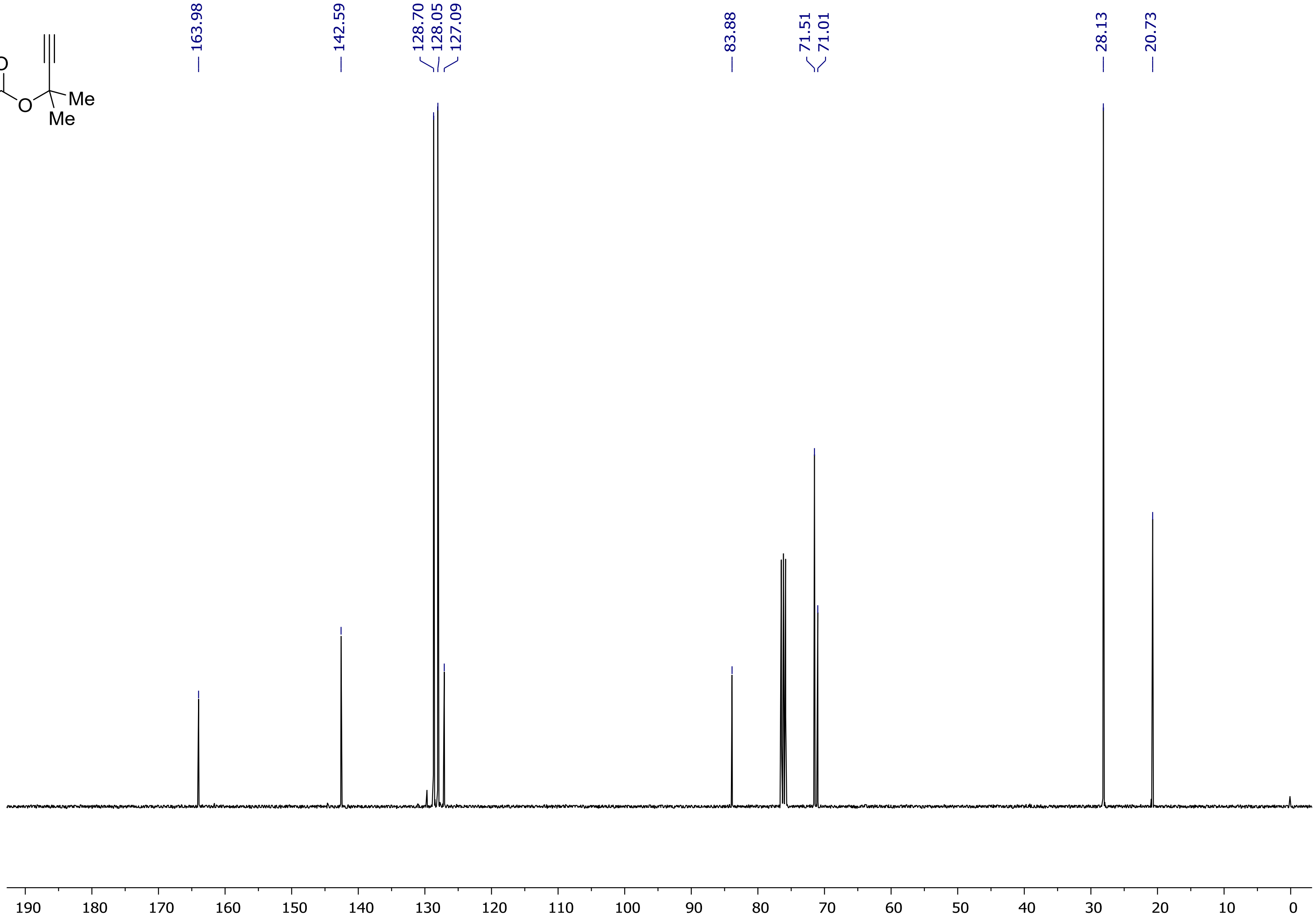

Figure S20 ^{1}H NMR (400 MHz, $CDCl_3$, 298 K) spectrum of 2-methylbut-3-yn-2-yl 4-fluorobenzoate (**2c**).

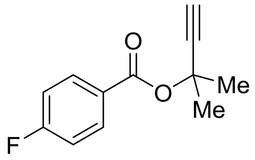

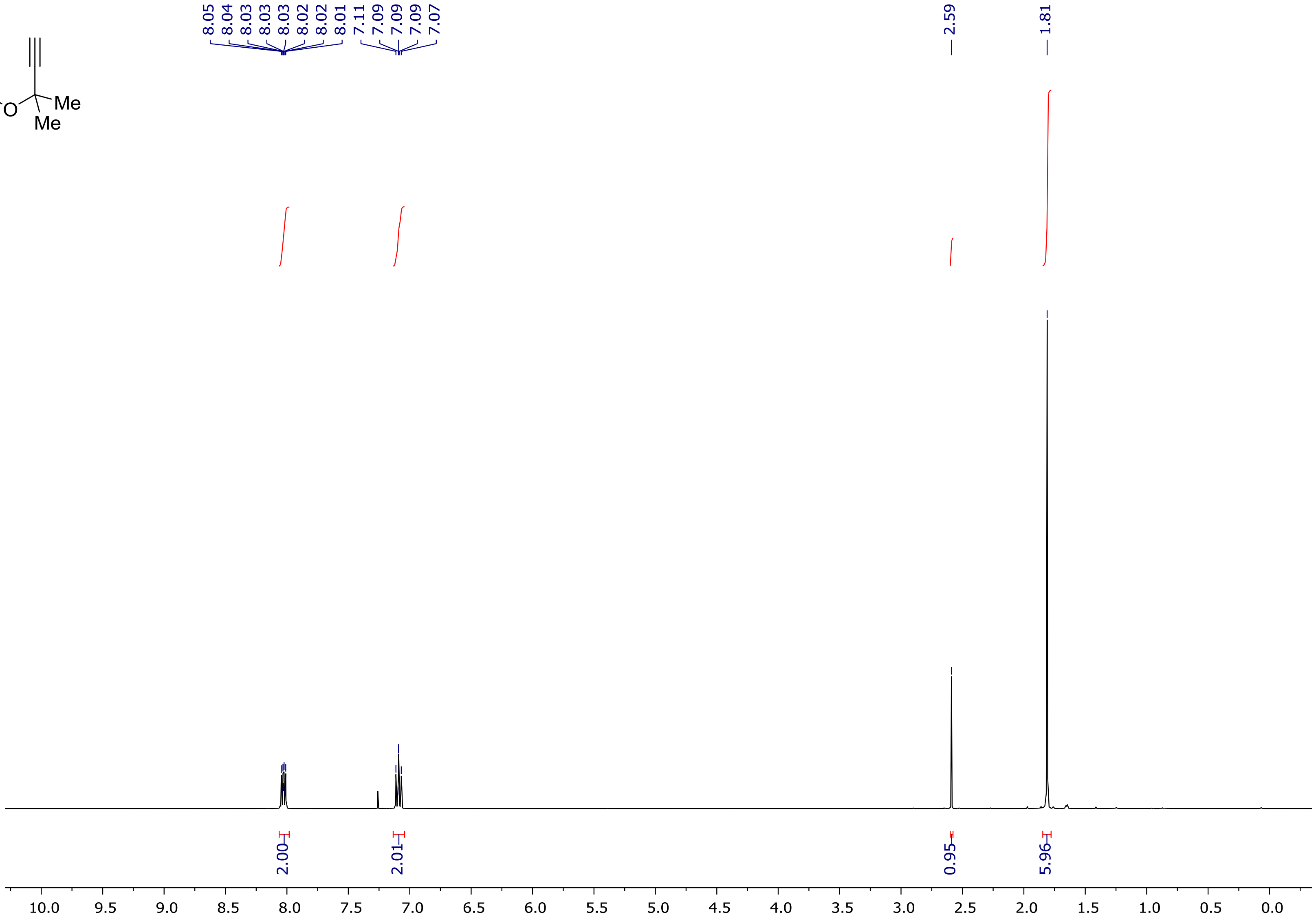

Figure S21 ^{13}C NMR (101 MHz, $CDCl_3$, 298 K) spectrum of 2-methylbut-3-yn-2-yl benzoate (**2c**).

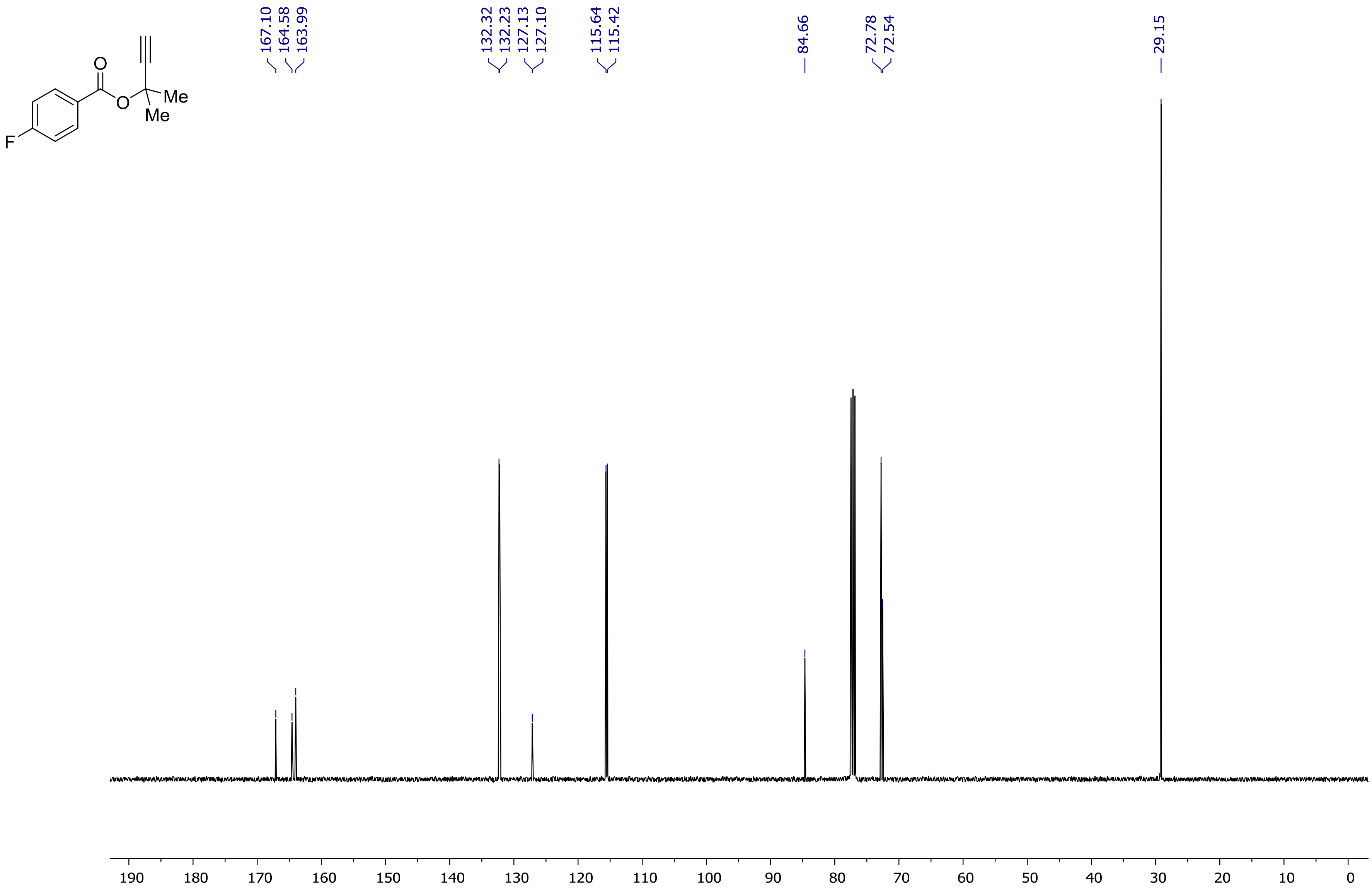

Figure S22 ^{19}F NMR (471 MHz, $CDCl_3$, 298 K) spectrum of 2-methylbut-3-yn-2-yl benzoate (**2c**).

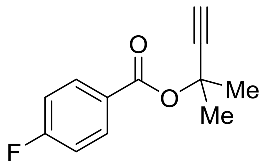

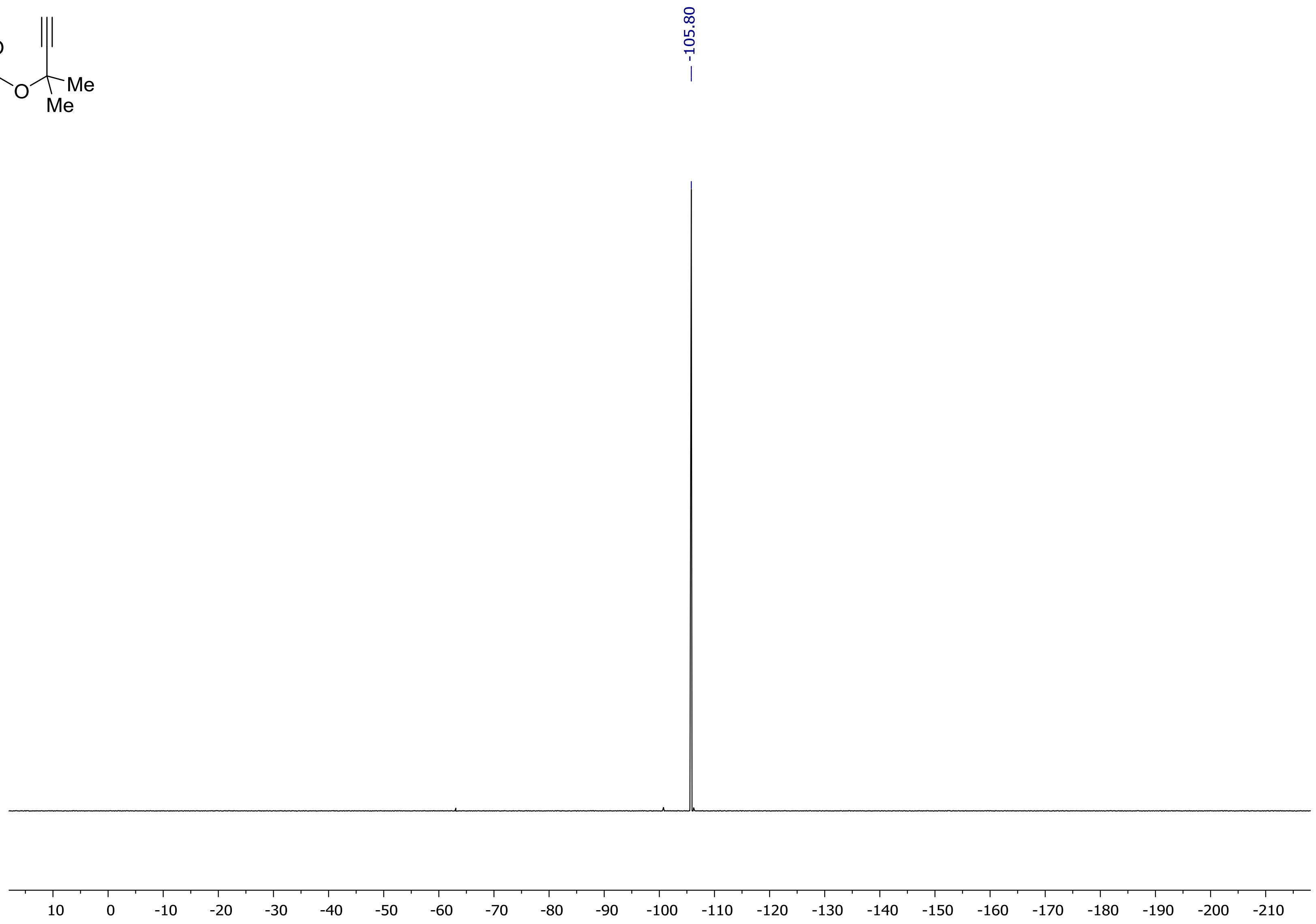

Figure S23 ^{1}H NMR (400 MHz, $CDCl_3$, 298 K) spectrum of but-3-yn-2-yl benzoate (**5**).

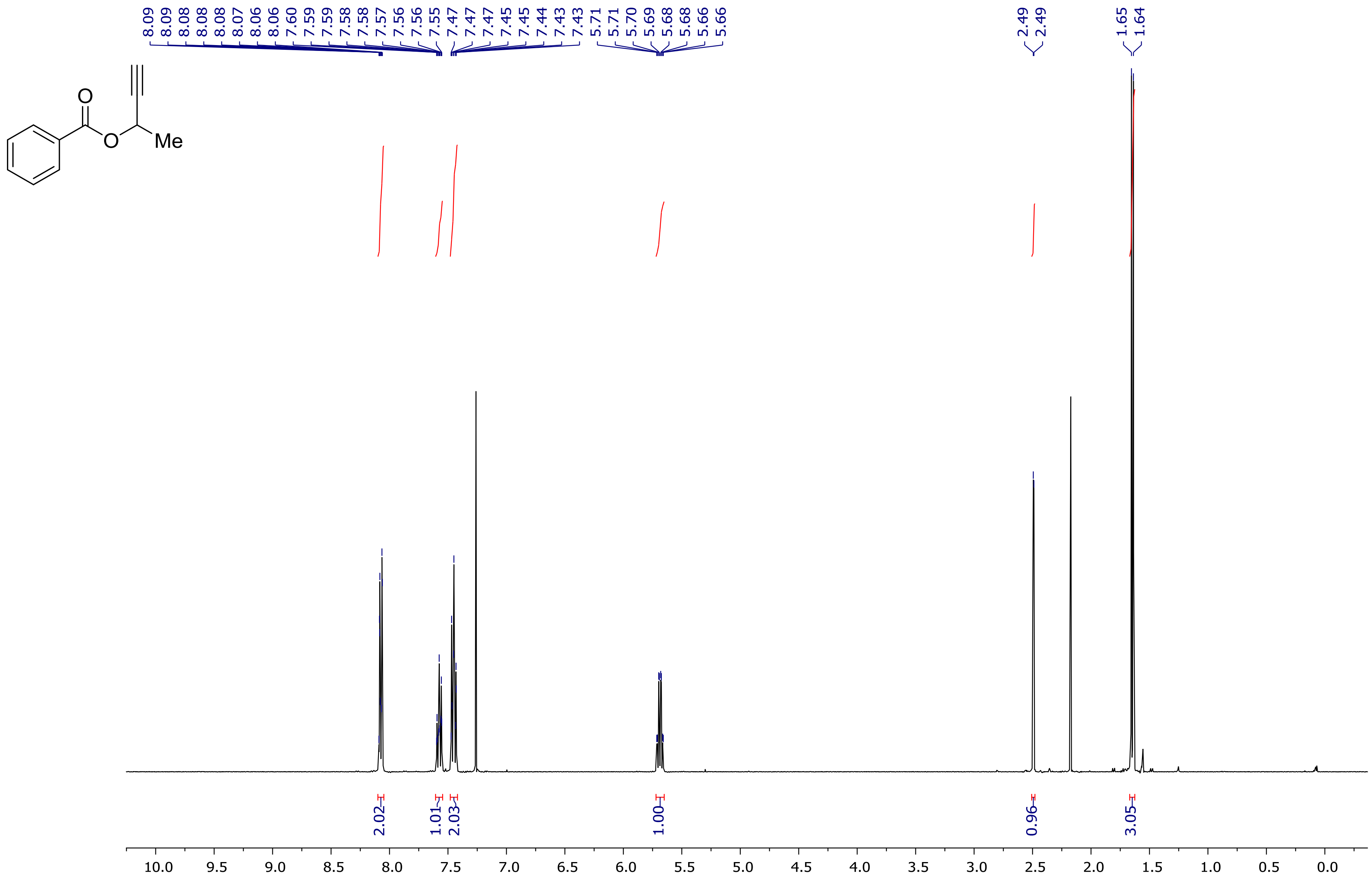

Figure S24 ^{13}C NMR (101 MHz, $CDCl_3$, 298 K) spectrum of but-3-yn-2-yl benzoate (**5**).

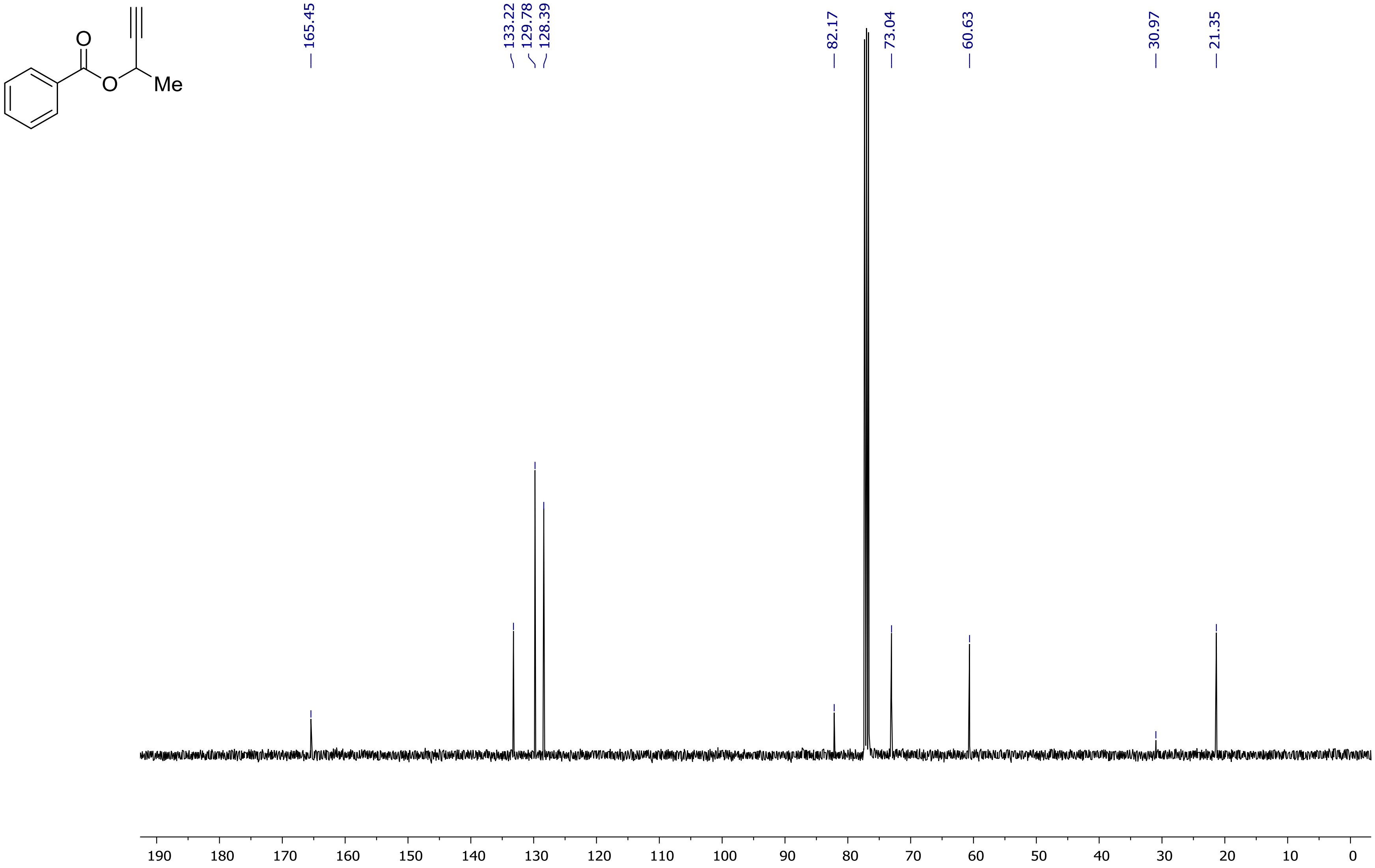

2.3 NMR spectra of products.

Figure S25 ^{1}H NMR (400 MHz, $CDCl_3$, 298 K) spectrum (*E*)-3-chloro-1-(chloro(phenyl)boryl)prop-1-en-2-yl benzoate (**3a**).

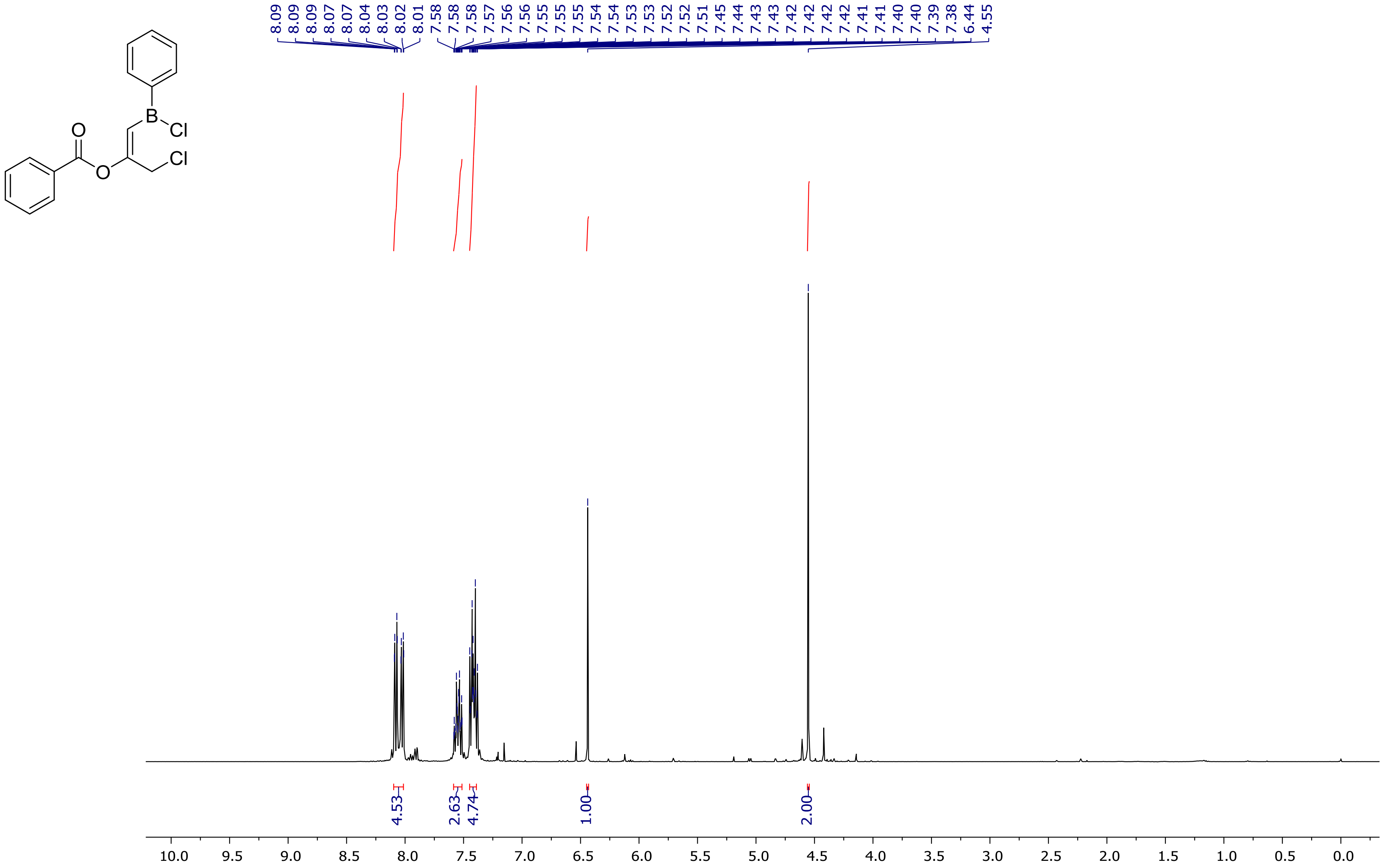

Figure S26 ^{13}C NMR (101 MHz, $CDCl_3$, 298 K) spectrum of (*E*)-3-chloro-1-(chloro(phenyl)boryl)prop-1-en-2-yl benzoate (**3a**).

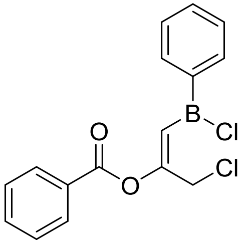

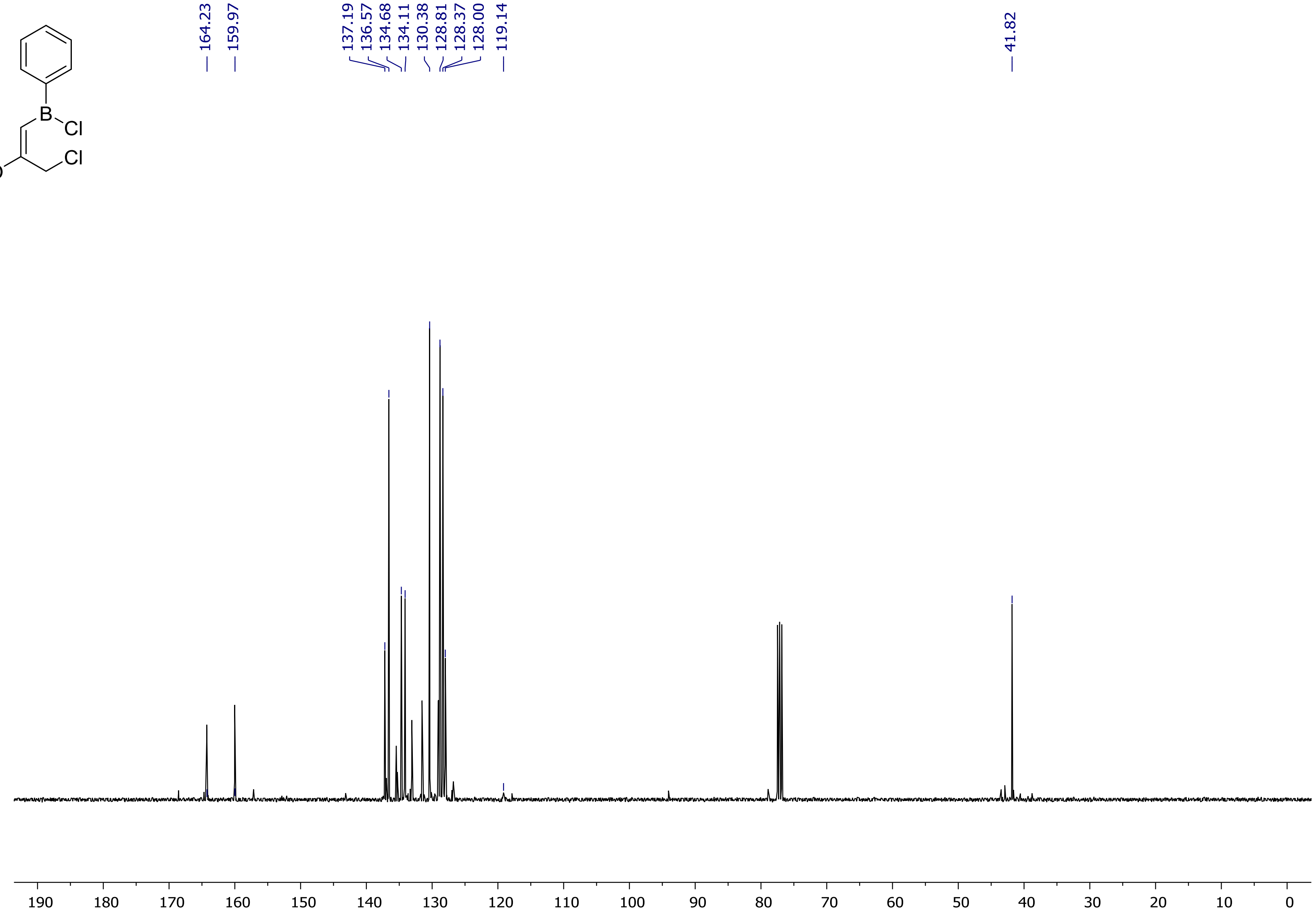

Figure S27 ^{11}B NMR (160 MHz, $CDCl_3$, 298 K) spectrum of (*E*)-3-chloro-1-(chloro(phenyl)boryl)prop-1-en-2-yl benzoate (**3a**).

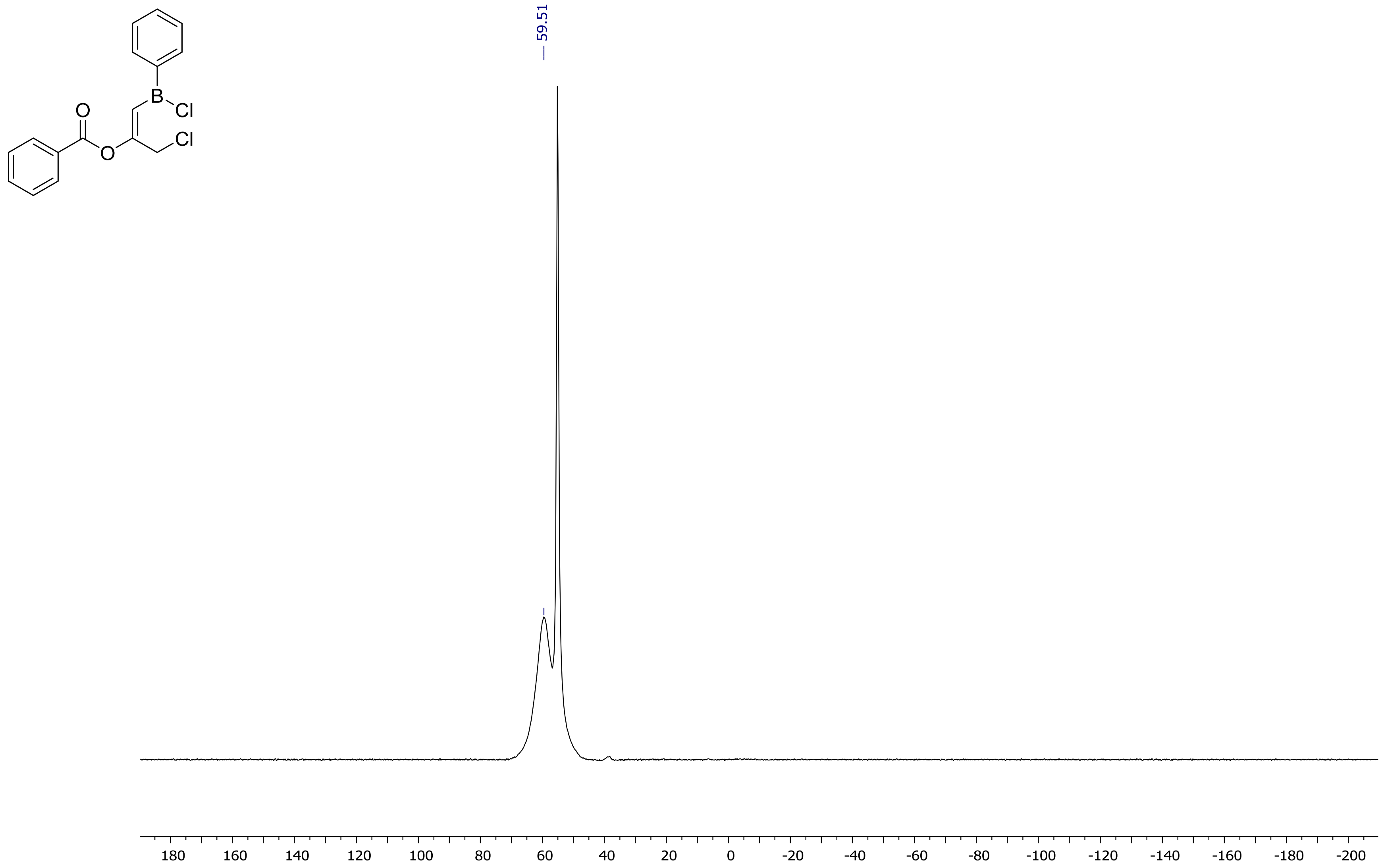

Figure S28 HSQC (^{1}H-^{13}C) spectrum of (*E*)-3-chloro-1-(chloro(phenyl)boryl)prop-1-en-2-yl benzoate (**3a**).

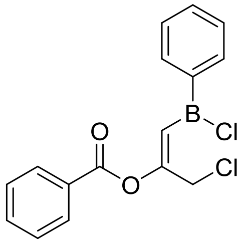

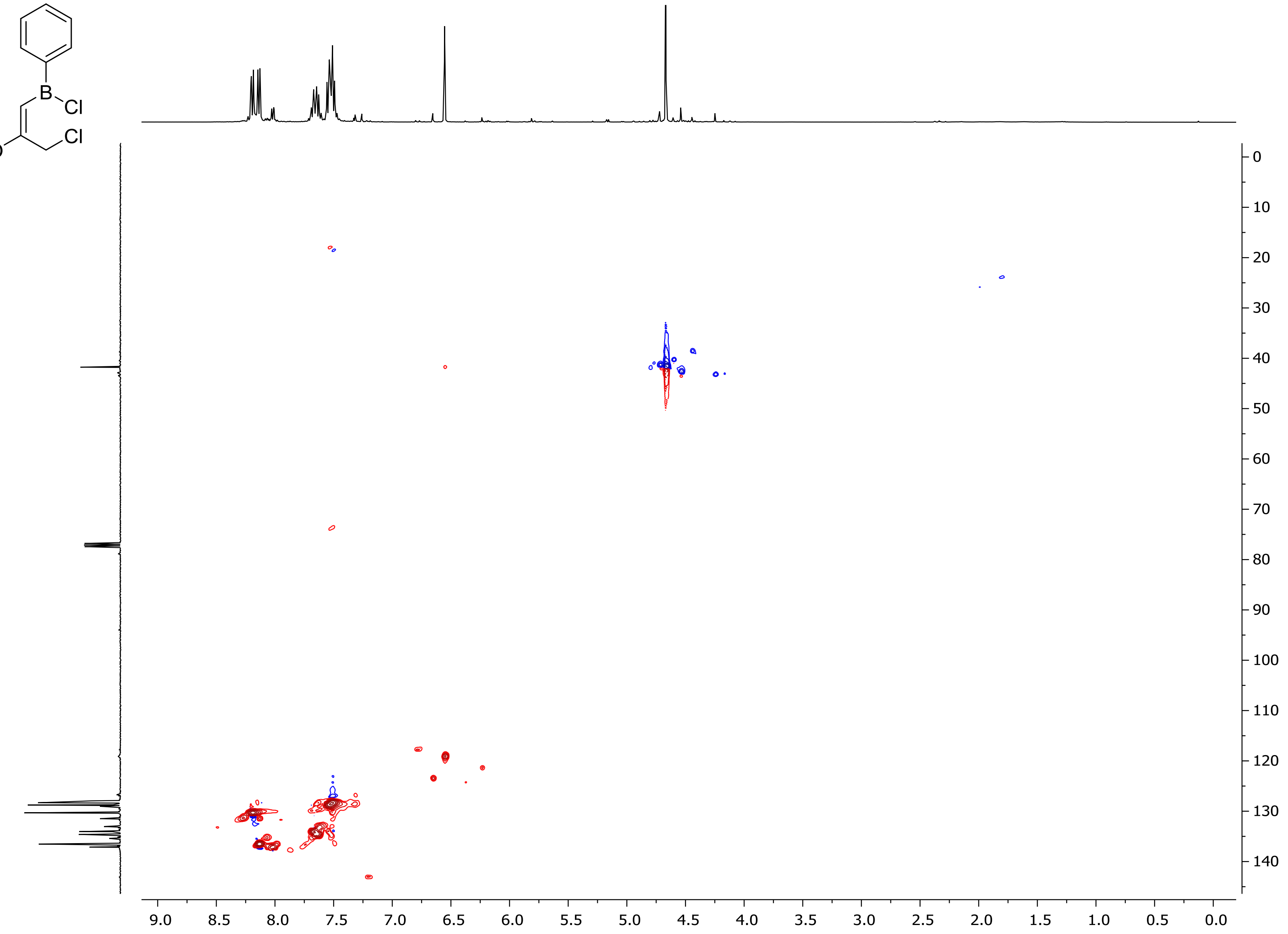

Figure S29 HMBC (^{1}H-^{13}C) spectrum of (*E*)-3-chloro-1-(chloro(phenyl)boryl)prop-1-en-2-yl benzoate (**3a**).

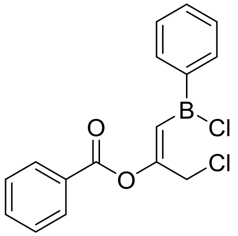

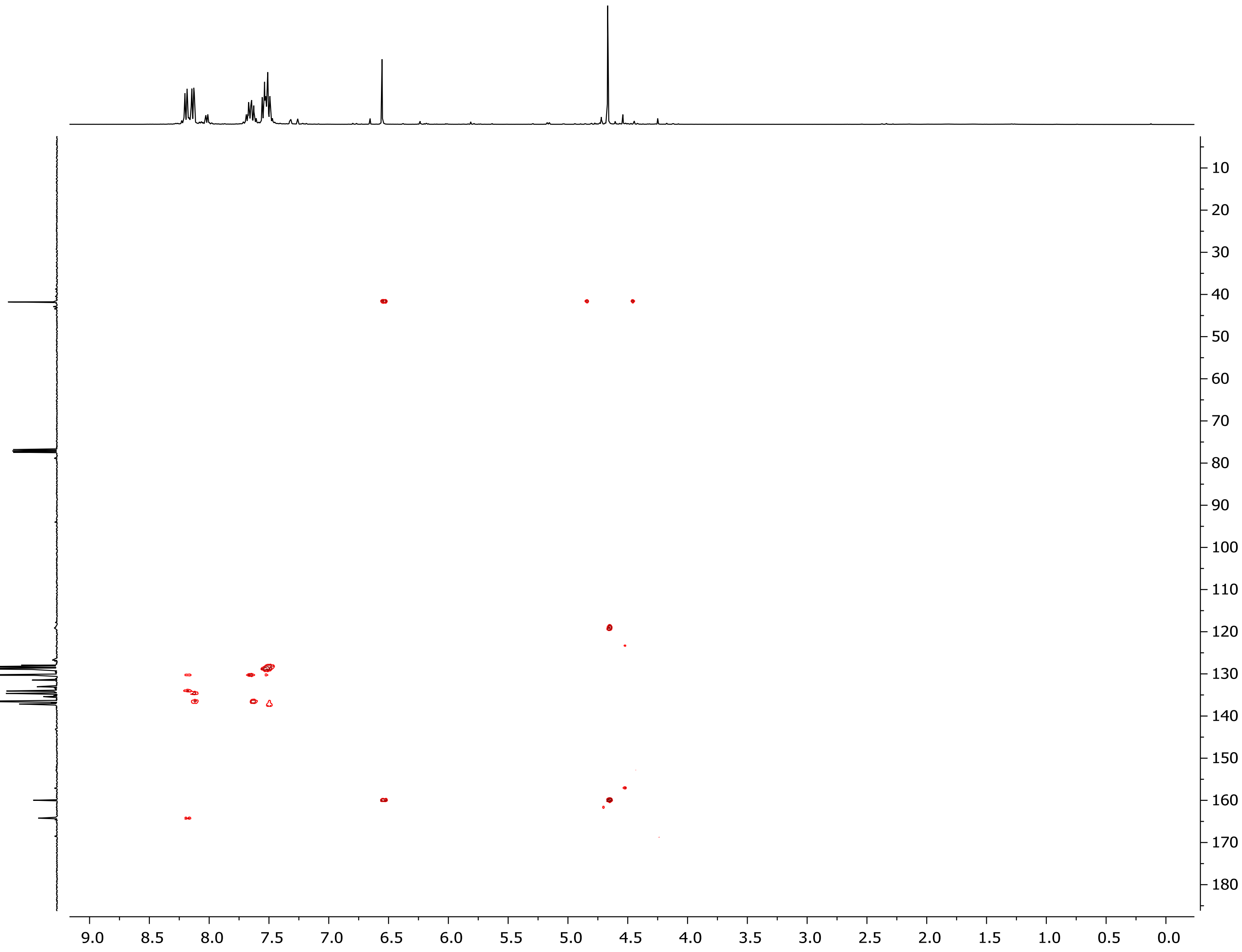

Figure S30 ^{1}H NMR (400 MHz, $CDCl_3$, 298 K) spectrum (*E*)-3-chloro-1-(chloro(phenyl)boryl)prop-1-en-2-yl 4-fluorobenzoate (**3b**).

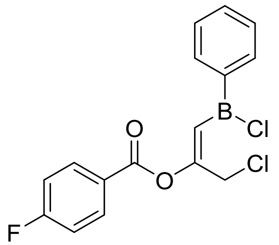

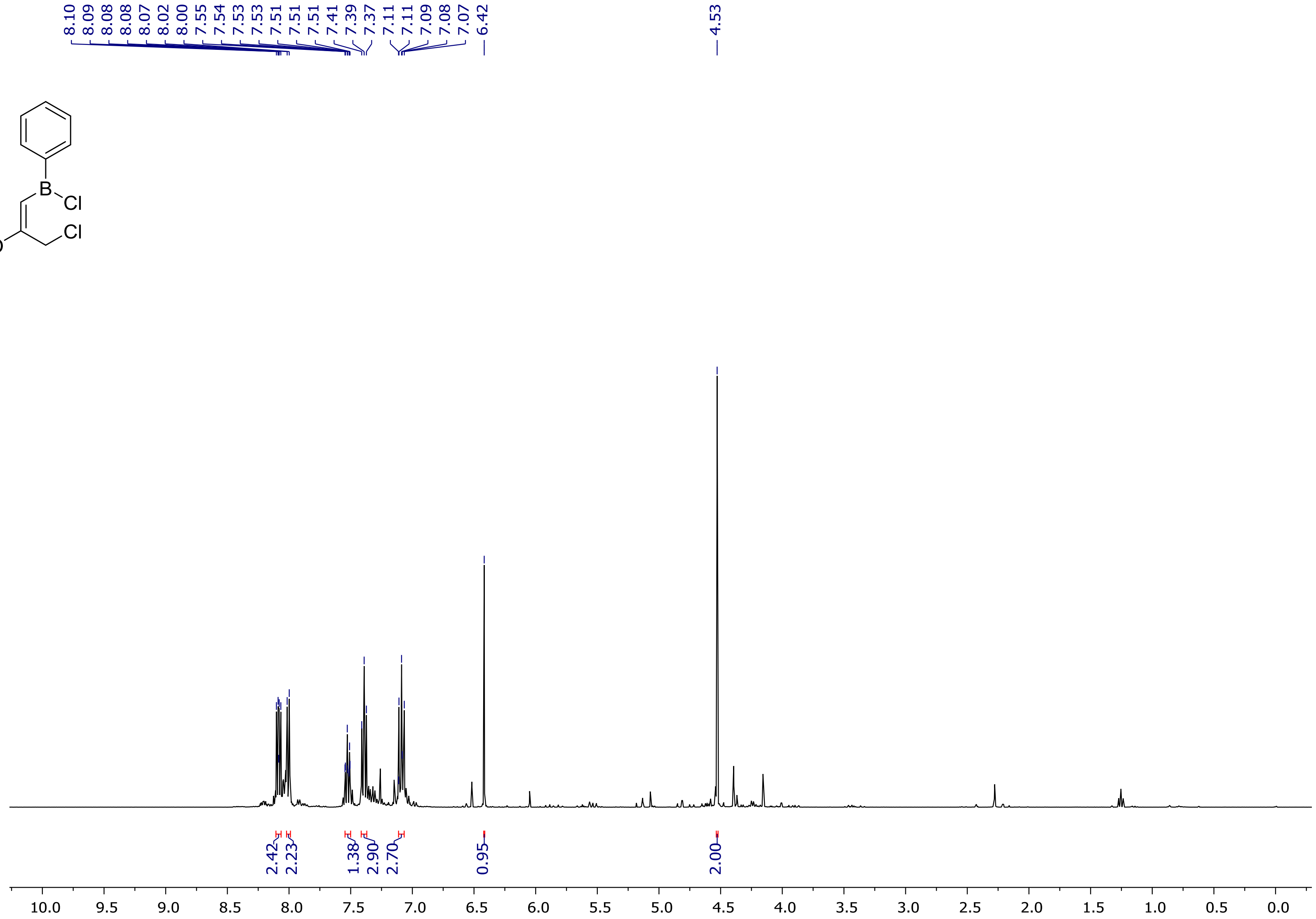

Figure S31 ^{13}C NMR (101 MHz, $CDCl_3$, 298 K) spectrum of (*E*)-3-chloro-1-(chloro(phenyl)boryl)prop-1-en-2-yl 4-fluorobenzoate (**3b**).

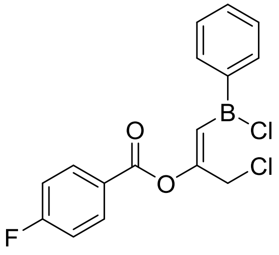

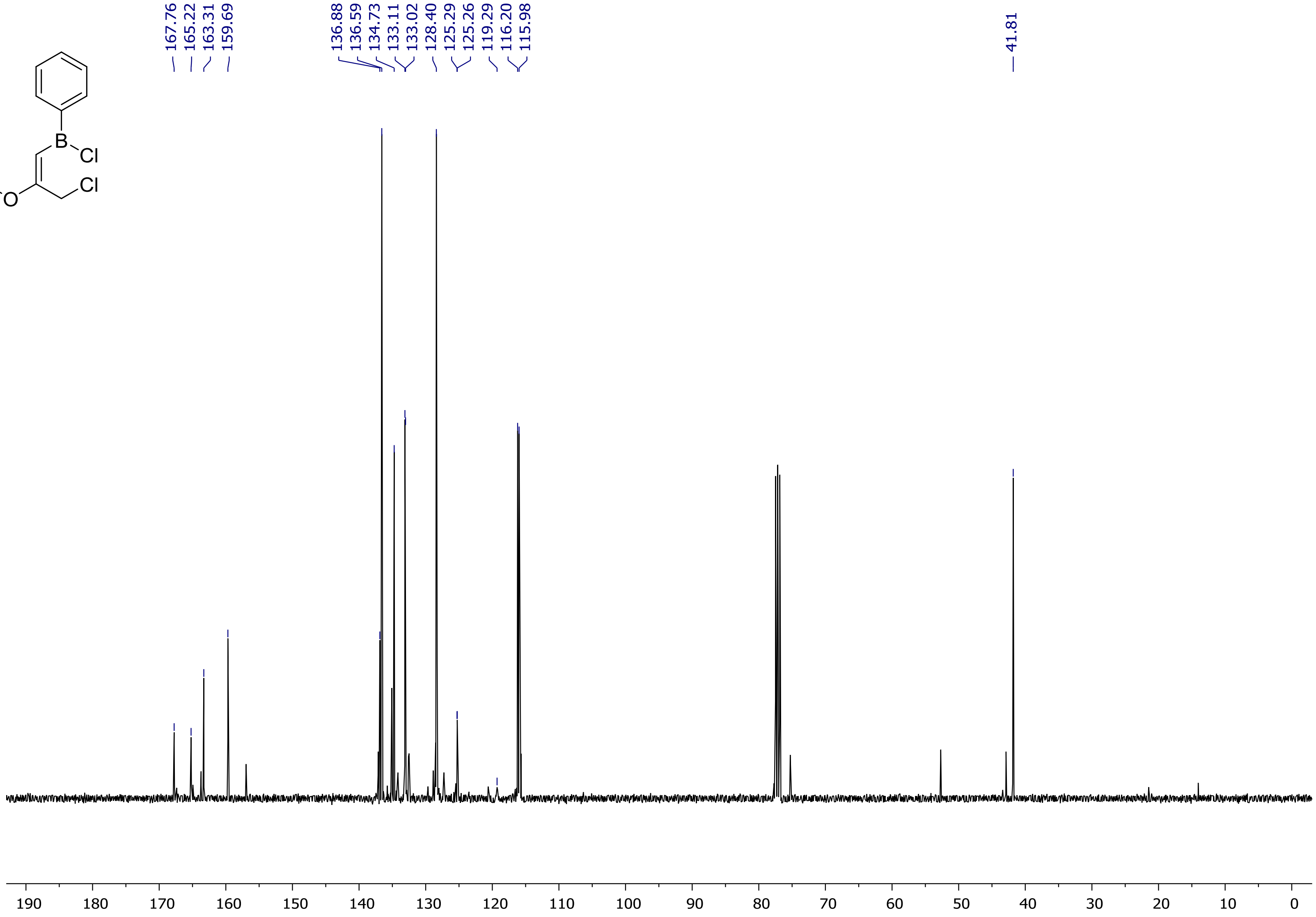

Figure S32 ^{11}B NMR (160 MHz, $CDCl_3$, 298 K) spectrum of (*E*)-3-chloro-1-(chloro(phenyl)boryl)prop-1-en-2-yl 4-fluorobenzoate (**3b**).

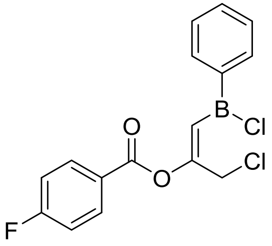

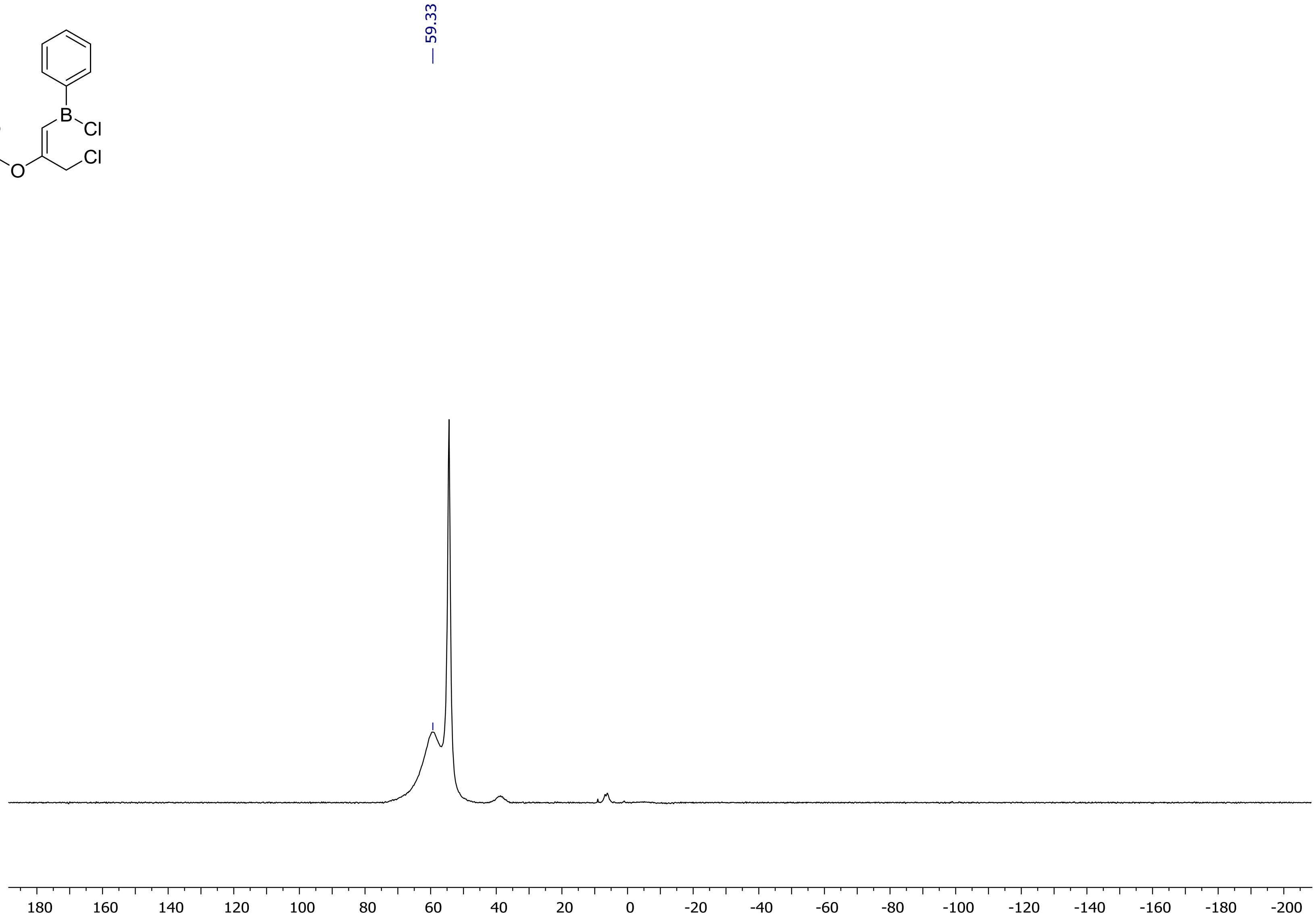

Figure S33 ^{19}F NMR (471 MHz, $CDCl_3$, 298 K) spectrum of (*E*)-3-chloro-1-(chloro(phenyl)boryl)prop-1-en-2-yl 4-fluorobenzoate (**3b**).

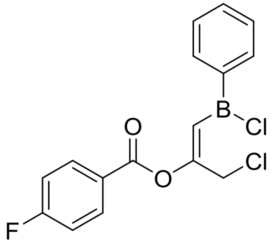

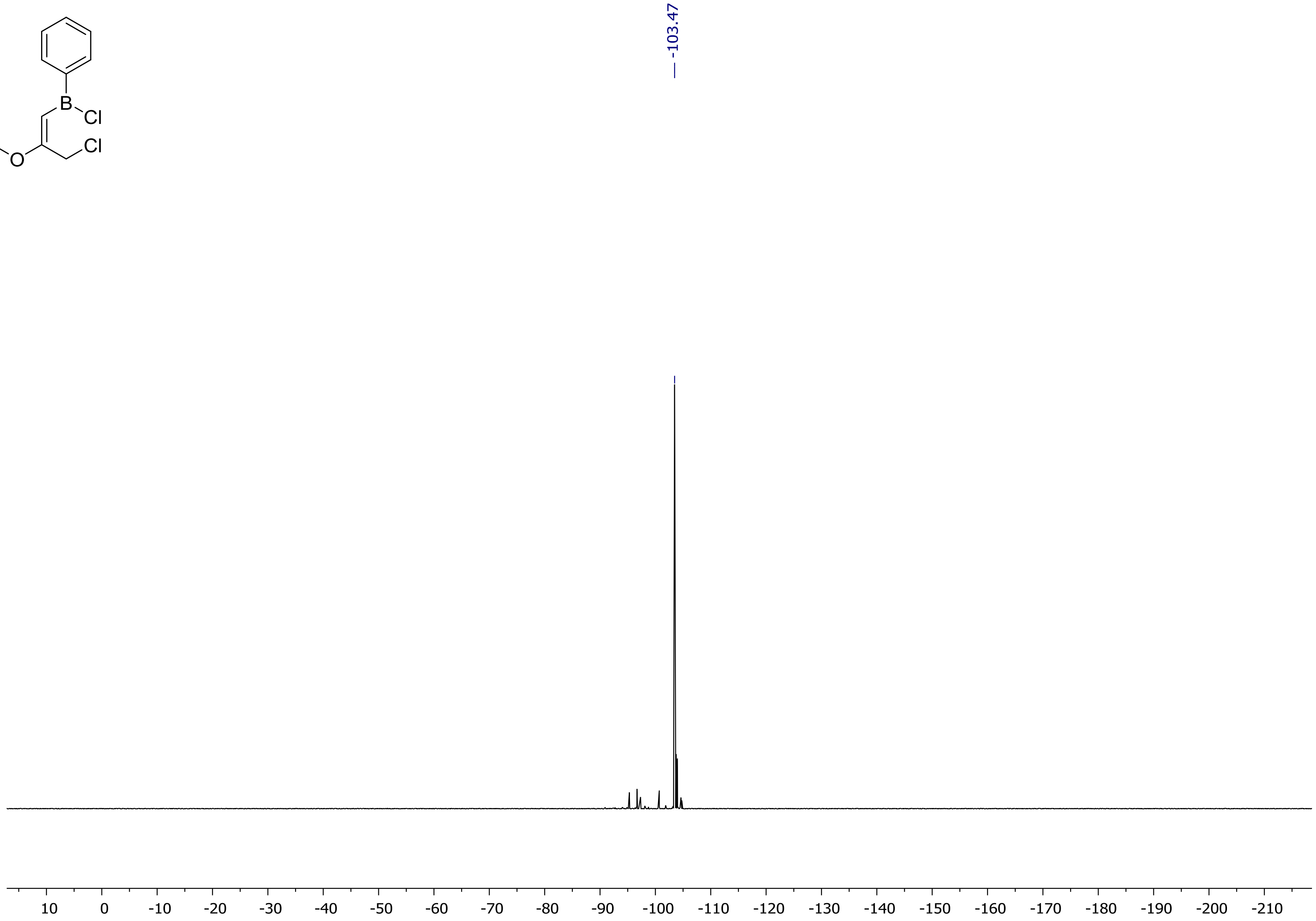

Figure S34 HSQC (^{1}H-^{13}C) spectrum of (*E*)-3-chloro-1-(chloro(phenyl)boryl)prop-1-en-2-yl 4-fluorobenzoate (**3b**).

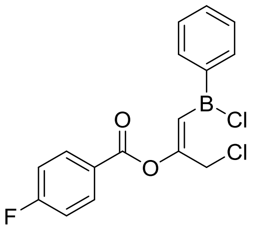

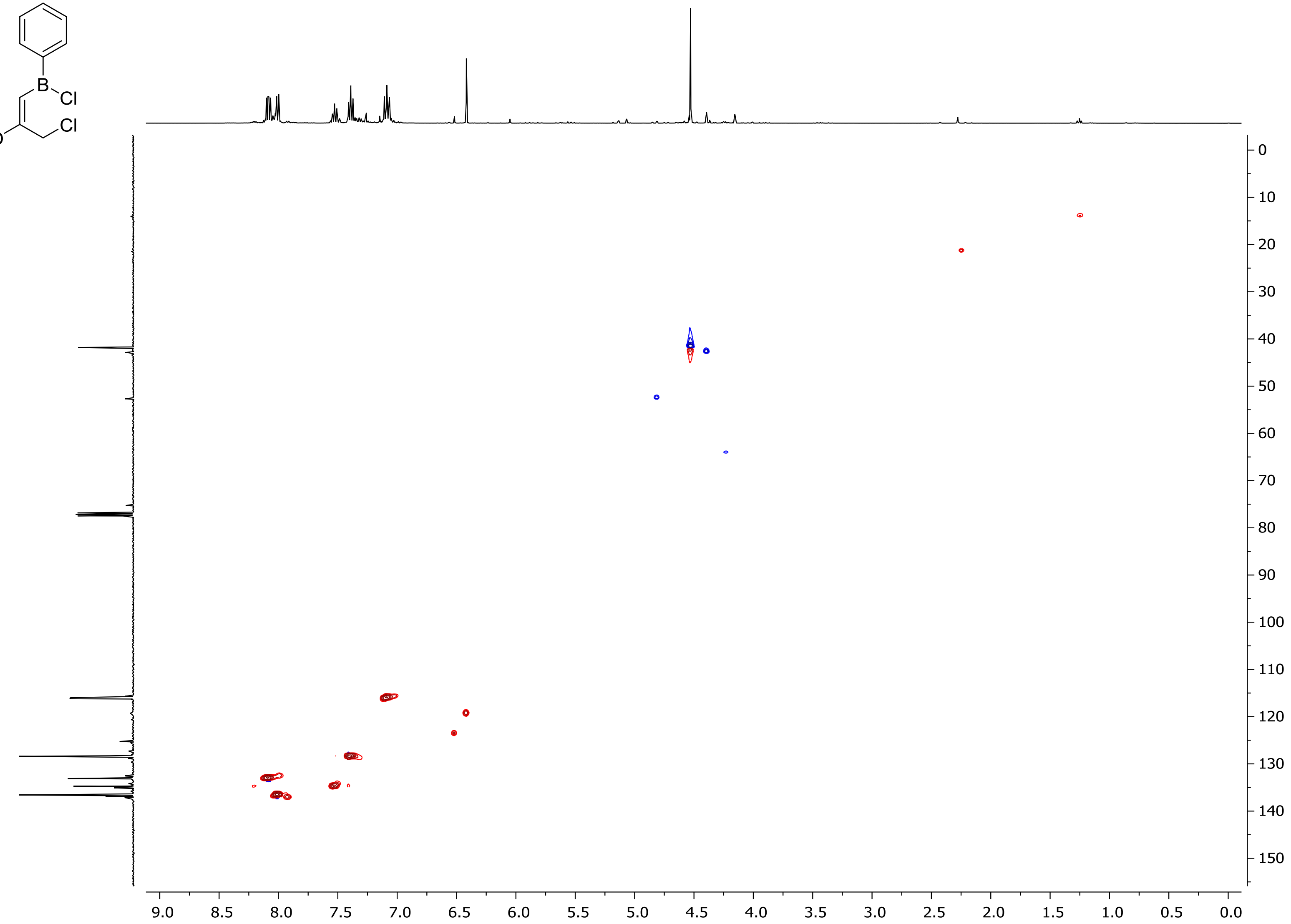

Figure S35 HMBC (^{1}H-^{13}C) spectrum of (*E*)-3-chloro-1-(chloro(phenyl)boryl)prop-1-en-2-yl 4-fluorobenzoate (**3b**).

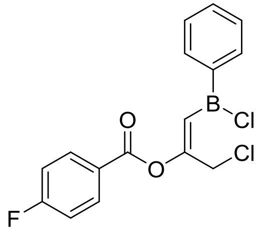

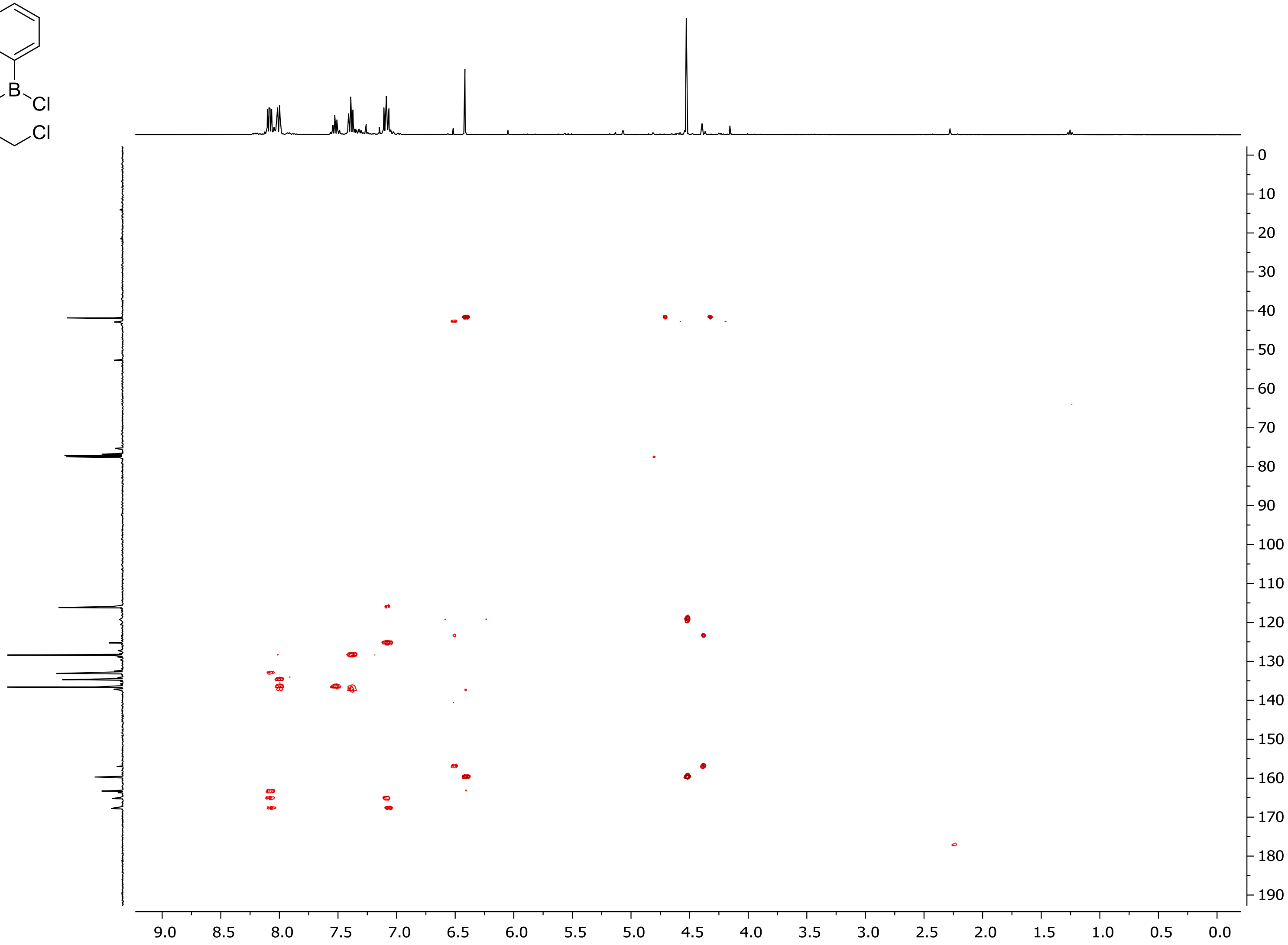

Figure S36 ^{1}H NMR (400 MHz, $CDCl_3$, 298 K) spectrum of (*E*)-3-chloro-1-(chloro(phenyl)boryl)prop-1-en-2-yl-4-nitro benzoate (**3c**).

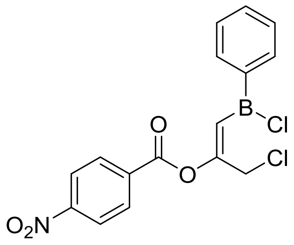

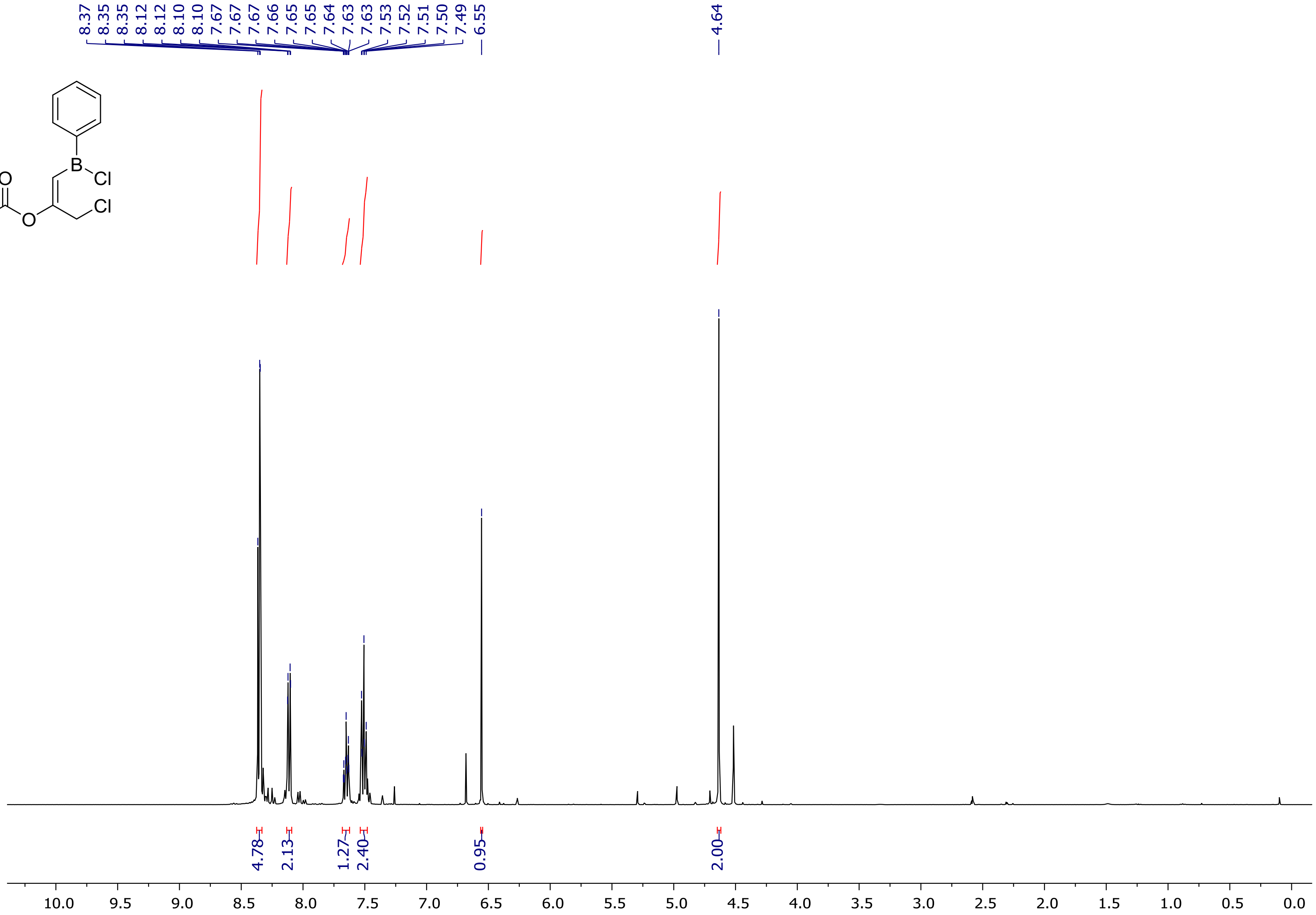

Figure S37 ^{13}C NMR (101 MHz, $CDCl_3$, 298 K) spectrum of (*E*)-3-chloro-1-(chloro(phenyl)boryl)prop-1-en-2-yl-4-nitro benzoate (**3c**).

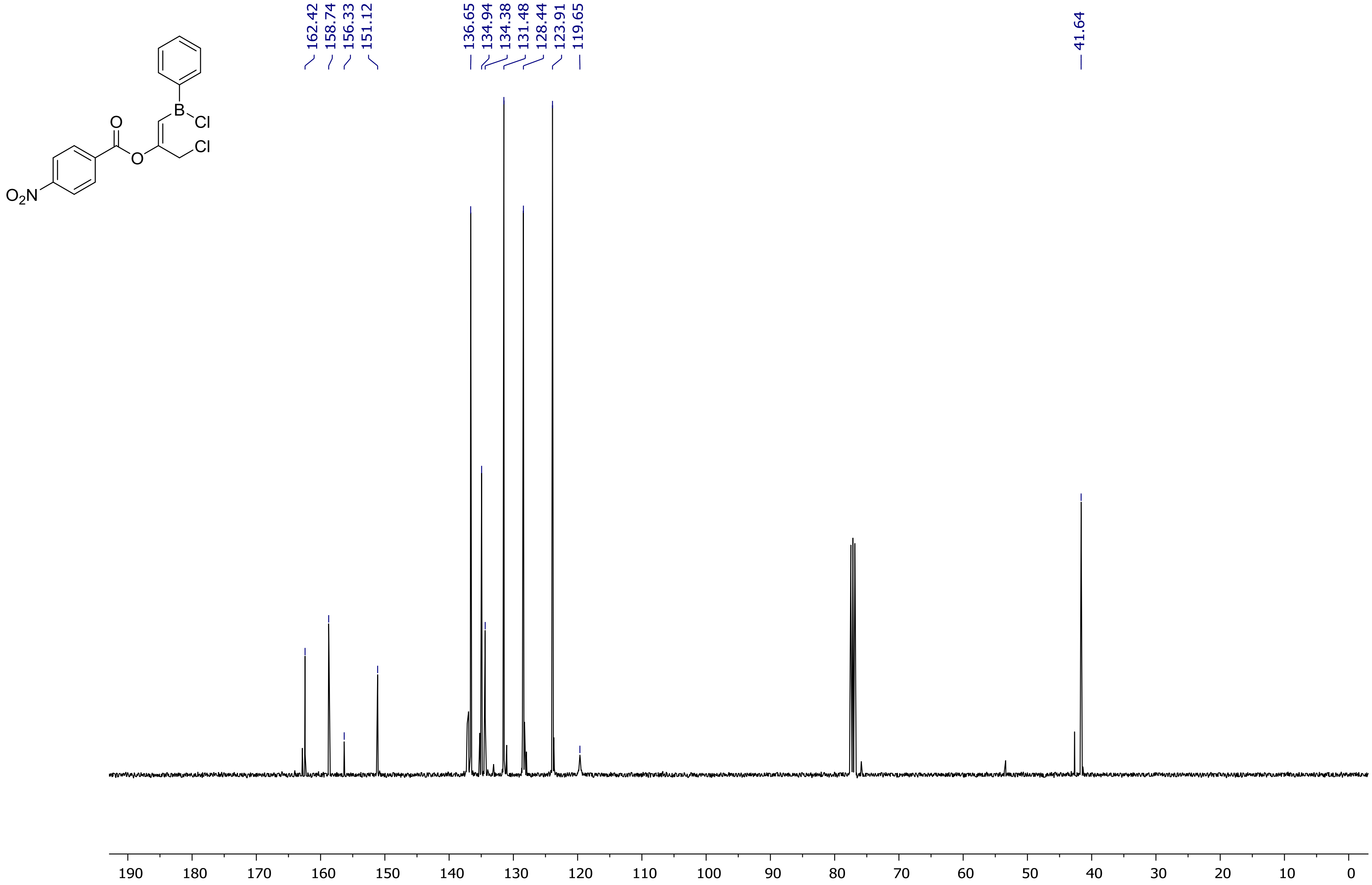

Figure S38 ^{11}B NMR (160 MHz, $CDCl_3$, 298 K) spectrum of (*E*)-3-chloro-1-(chloro(phenyl)boryl)prop-1-en-2-yl-4-nitro benzoate (**3c**).

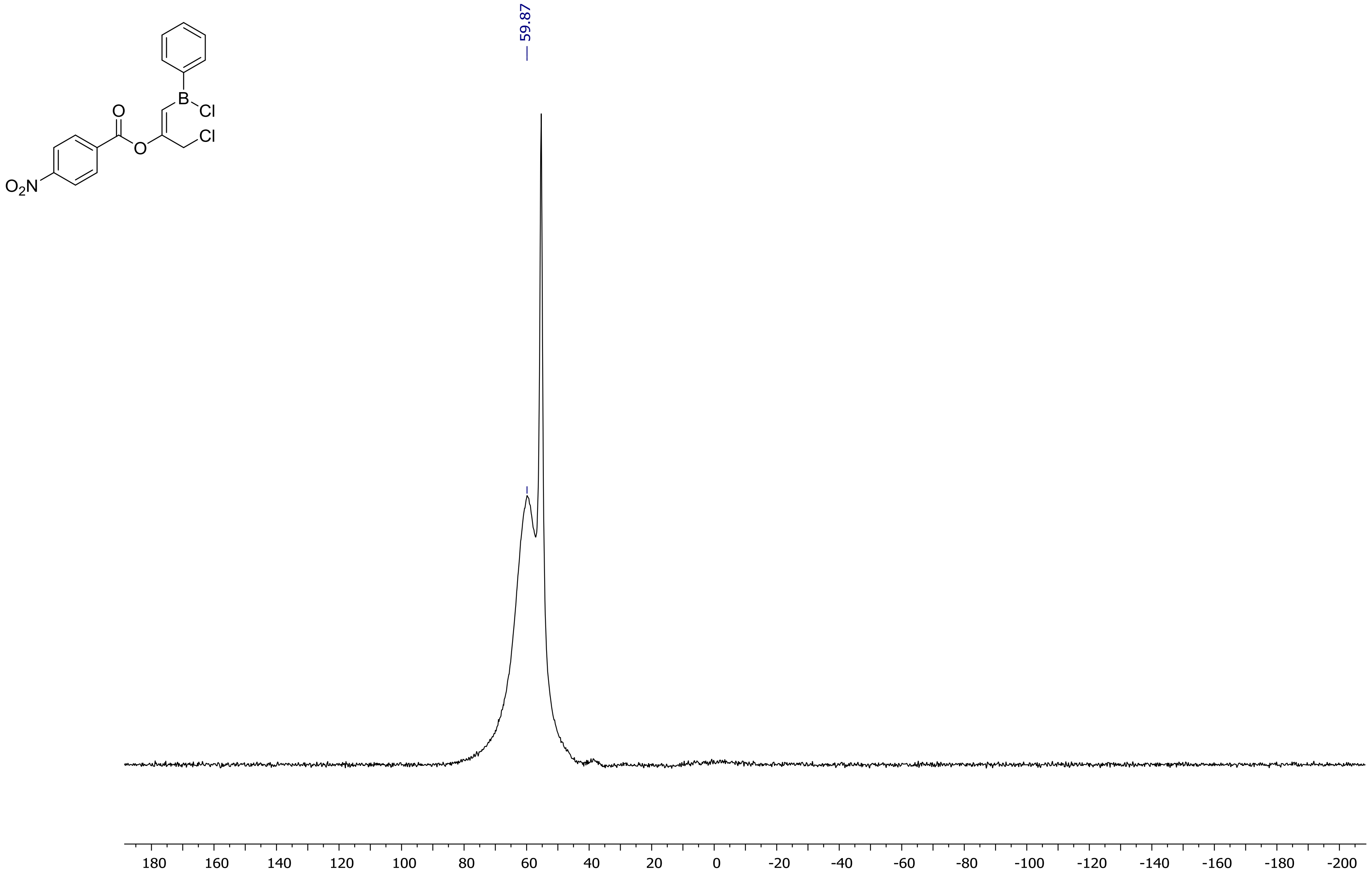

Figure S39 HSQC (^{1}H-^{13}C) spectrum of (*E*)-3-chloro-1-(chloro(phenyl)boryl)prop-1-en-2-yl-4-nitro benzoate (**3c**).

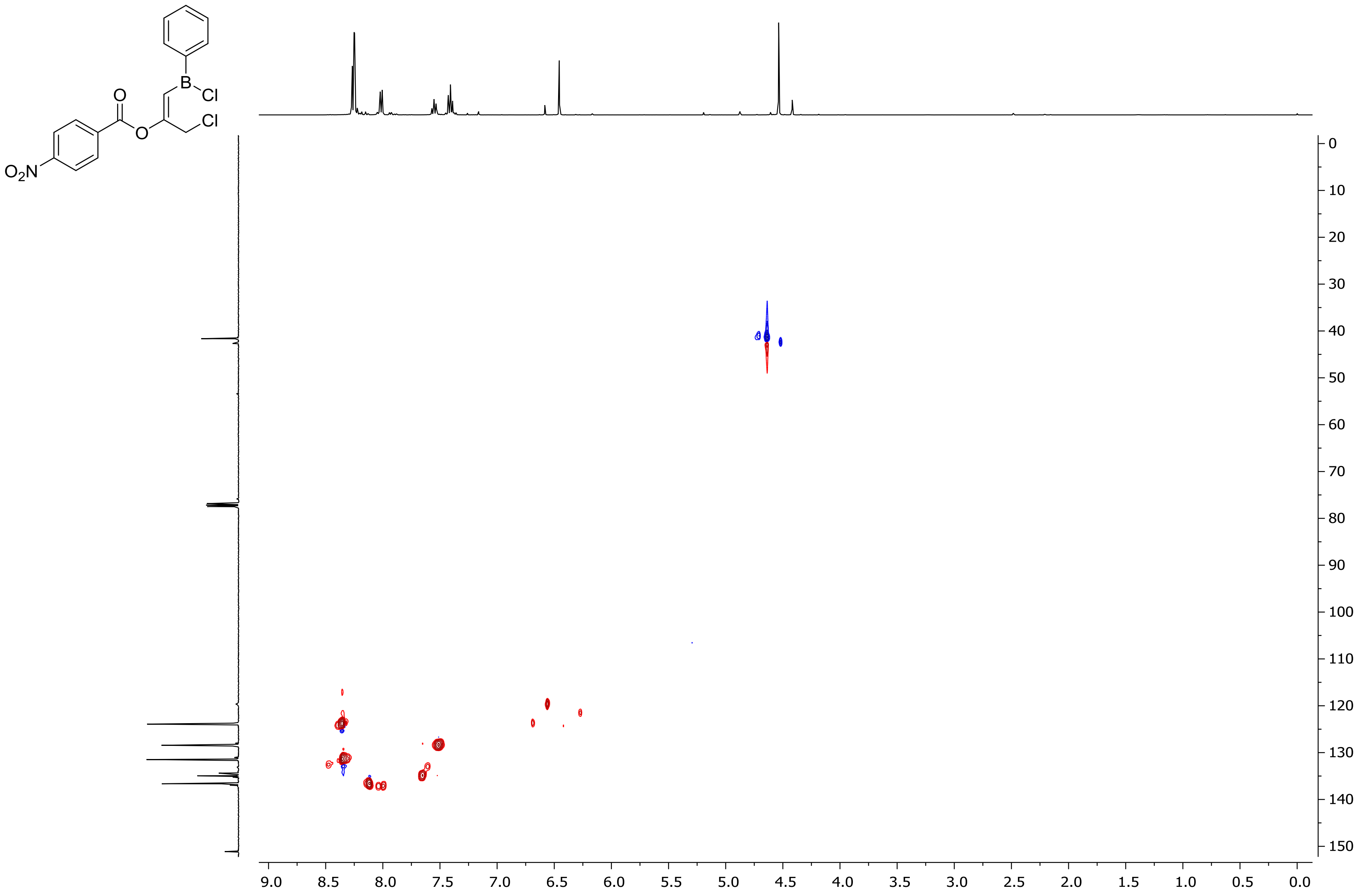

Figure S40 HMBC (^{1}H-^{13}C) spectrum of (*E*)-3-chloro-1-(chloro(phenyl)boryl)prop-1-en-2-yl-4-nitro benzoate (**3c**).

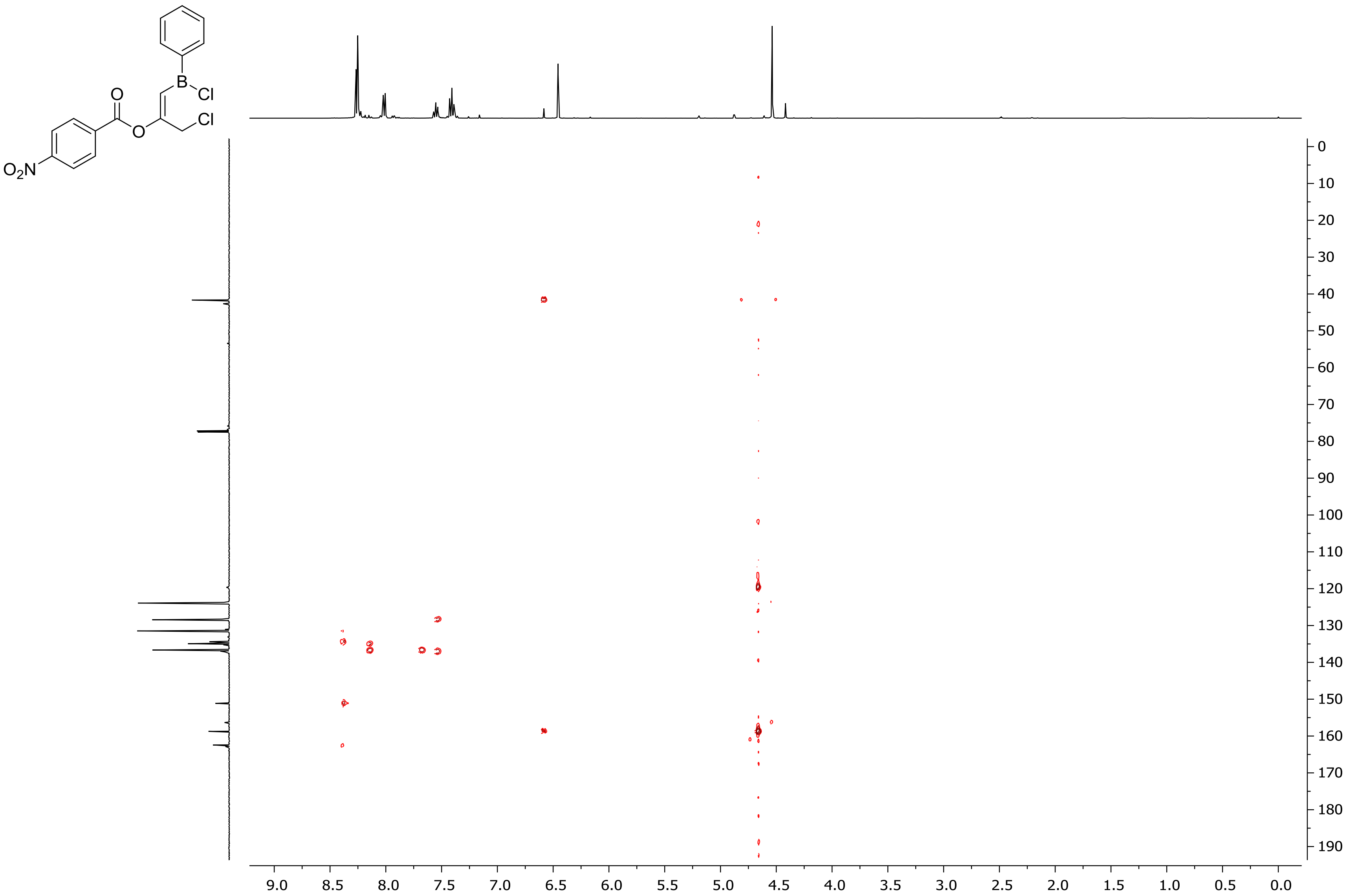

Figure S41 ^{1}H NMR (400 MHz, $CDCl_3$, 298 K) spectrum of (*E*)-3-chloro-1-(chloro(phenyl)boryl)prop-1-en-2-yl-3-nitro benzoate (**3d**).

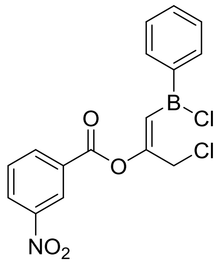

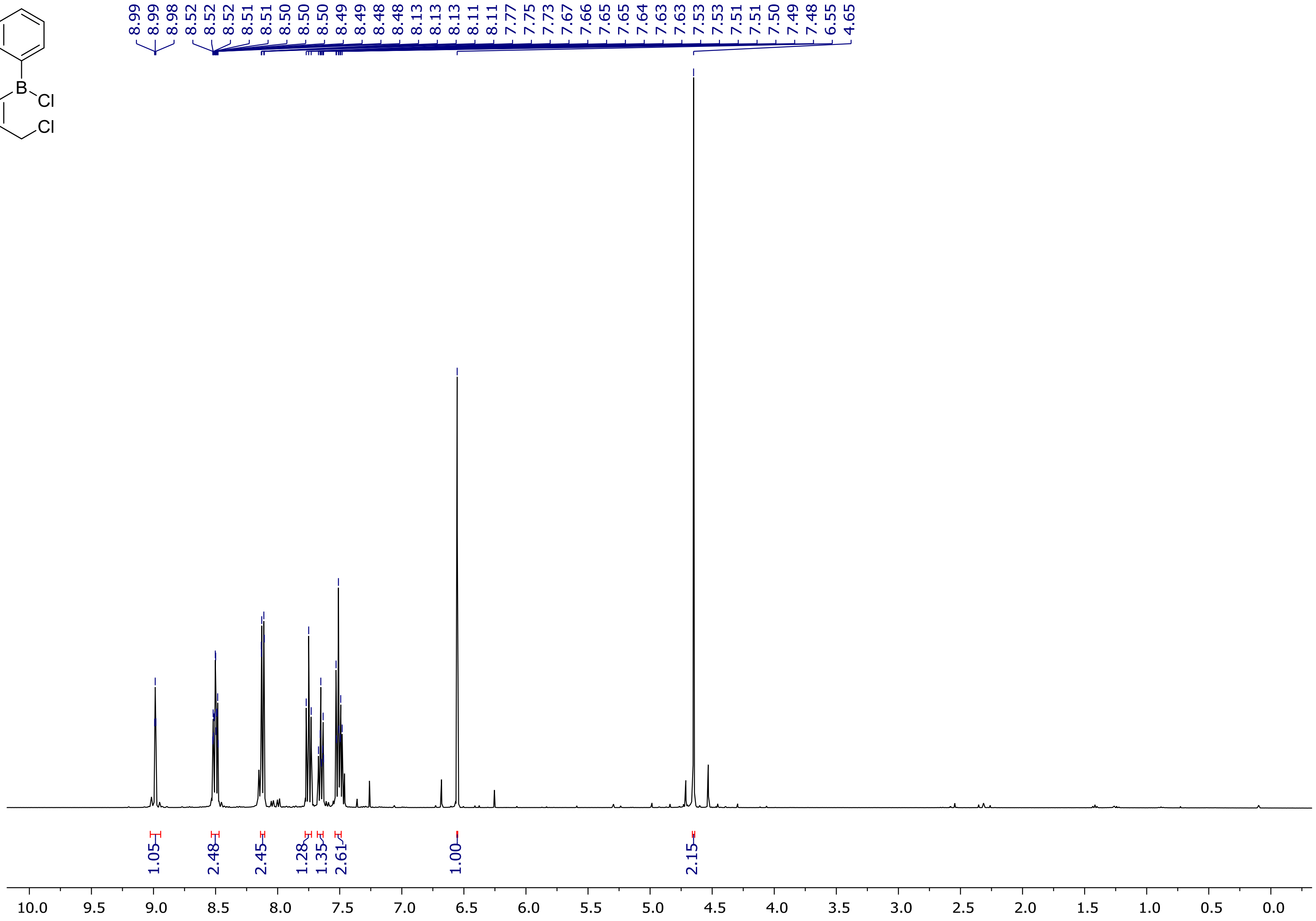

Figure S42 ^{13}C NMR (101 MHz, $CDCl_3$, 298 K) spectrum of (*E*)-3-chloro-1-(chloro(phenyl)boryl)prop-1-en-2-yl-3-nitro benzoate (**3d**).

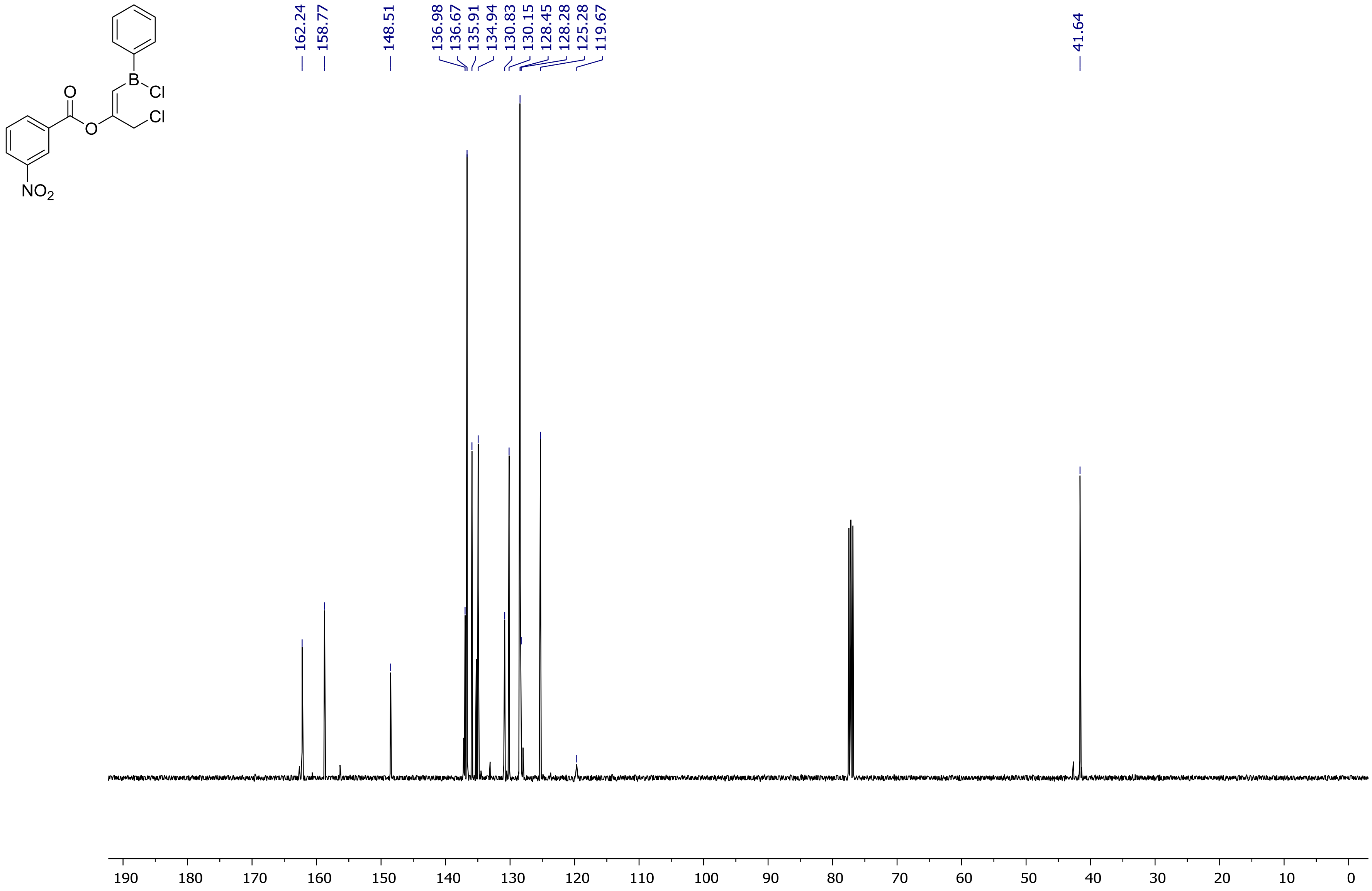

Figure S43 ^{11}B NMR (160 MHz, $CDCl_3$, 298 K) spectrum of (*E*)-3-chloro-1-(chloro(phenyl)boryl)prop-1-en-2-yl-3-nitro benzoate (**3d**).

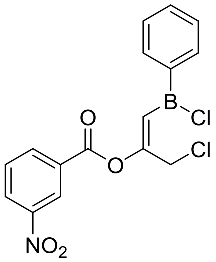

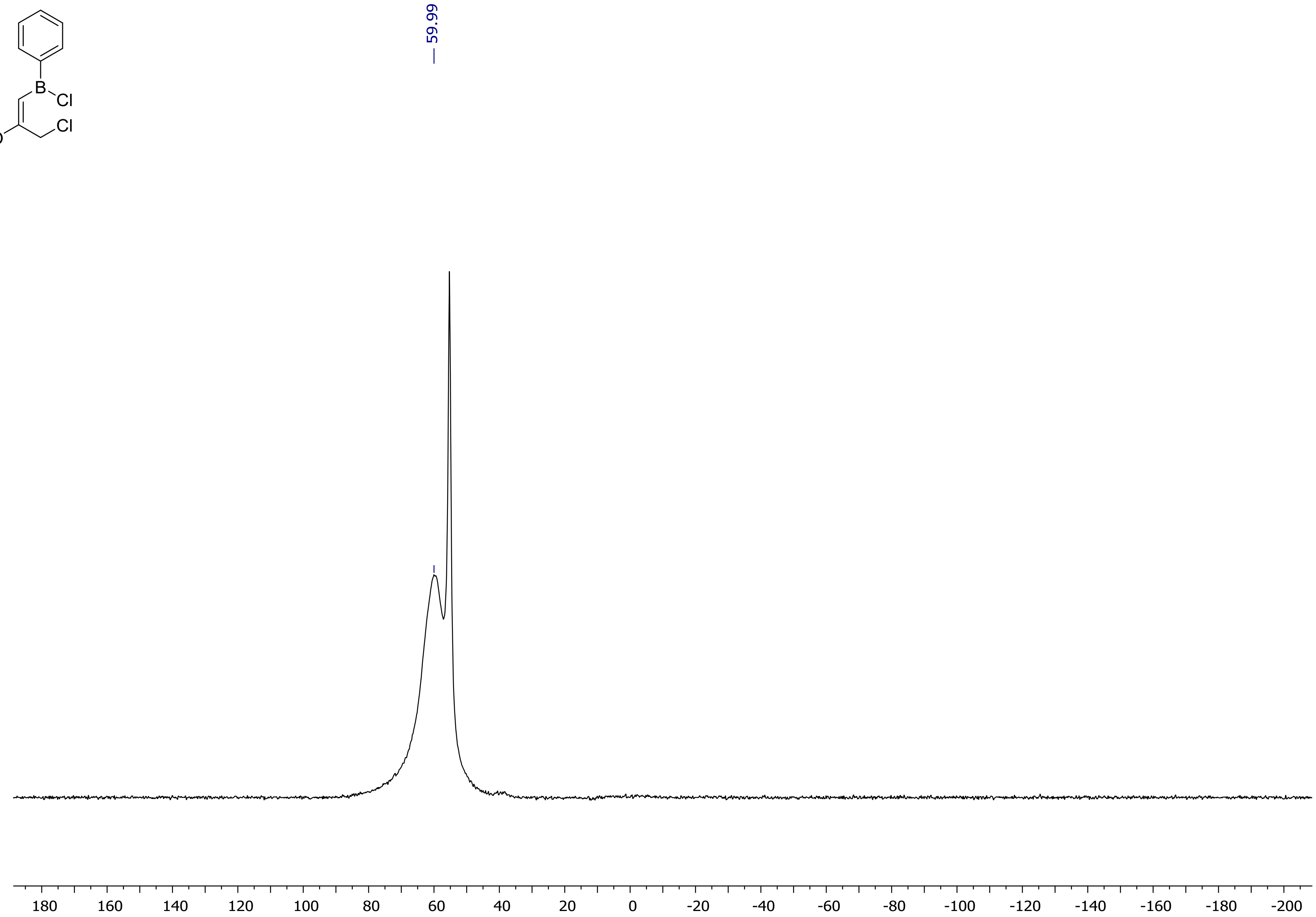

Figure S44 HSQC (^{1}H-^{13}C) spectrum of (*E*)-3-chloro-1-(chloro(phenyl)boryl)prop-1-en-2-yl-3-nitro benzoate (**3d**).

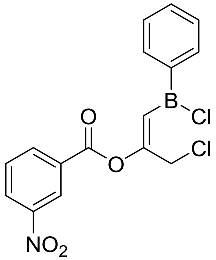

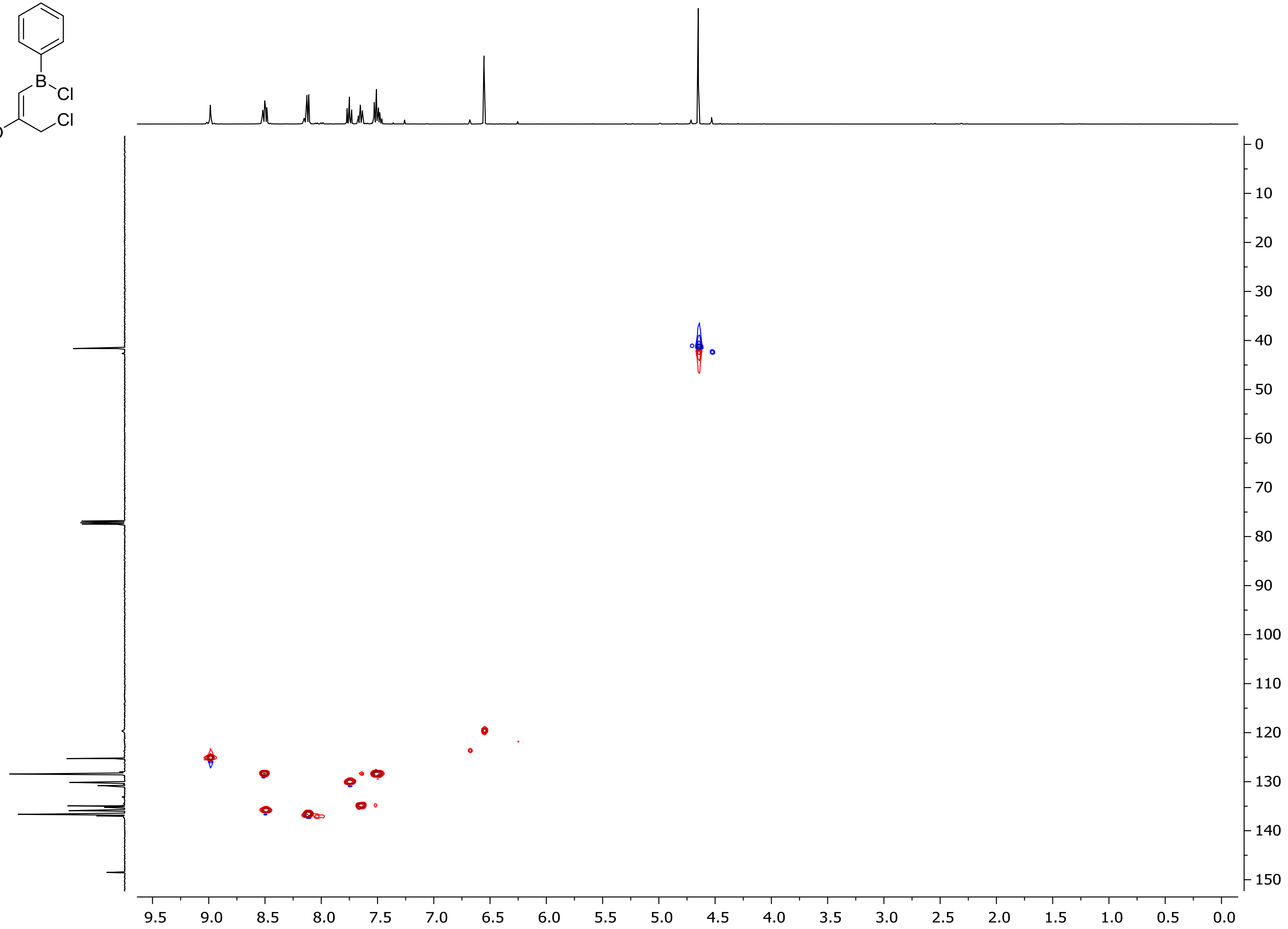

Figure S45 HMBC (^{1}H-^{13}C) spectrum of (*E*)-3-chloro-1-(chloro(phenyl)boryl)prop-1-en-2-yl-3-nitro benzoate (**3d**).

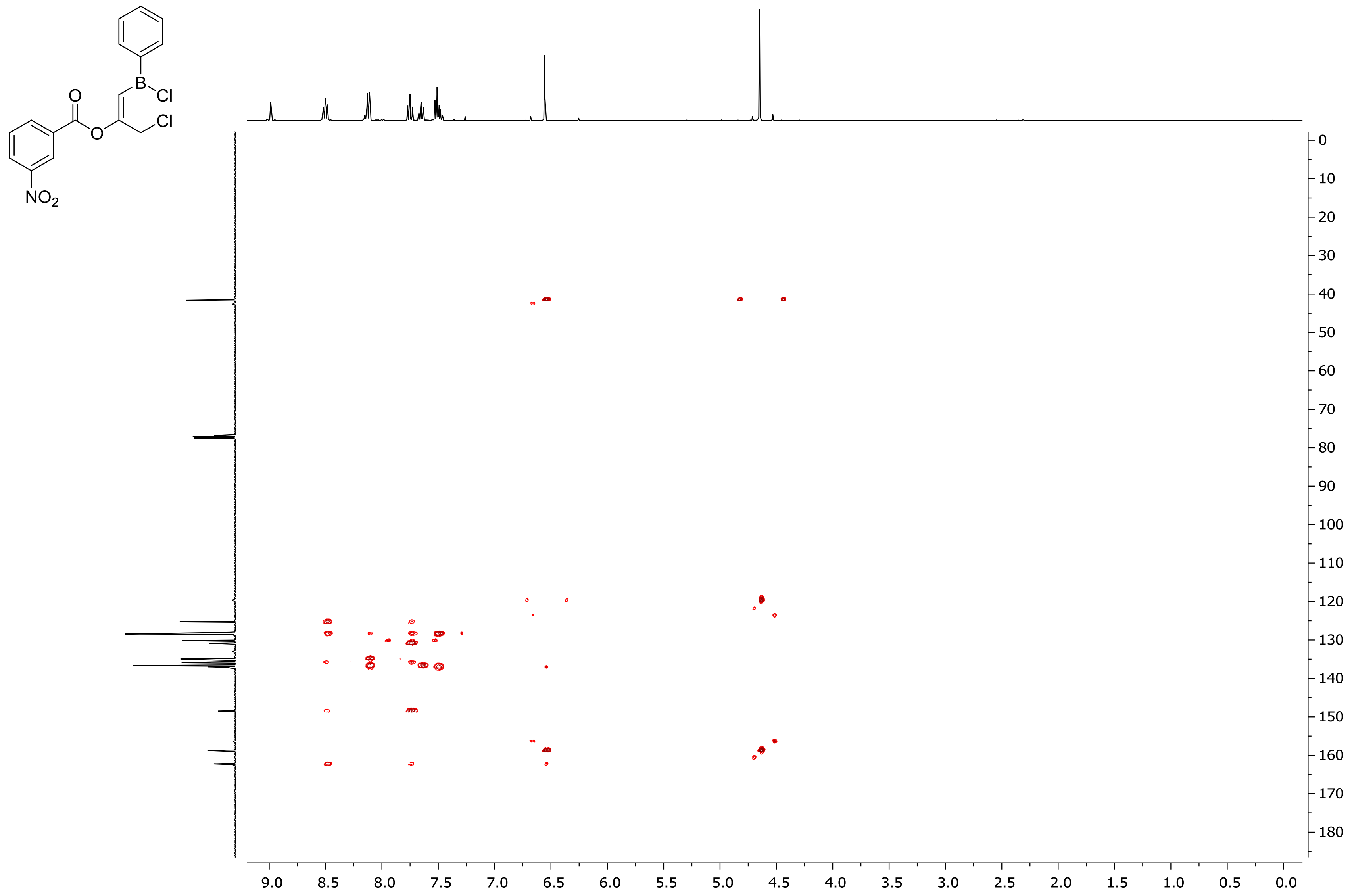

Figure S46 ^{1}H NMR (400 MHz, $CDCl_3$, 298 K) spectrum 2,3-dichloro-2,6-diphenyl-4-(propan-2-ylidene)-3,4-dihydro-2H-1,5,2-dioxaborinin-1-ium-2-uide (**4a**).

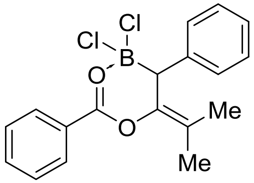

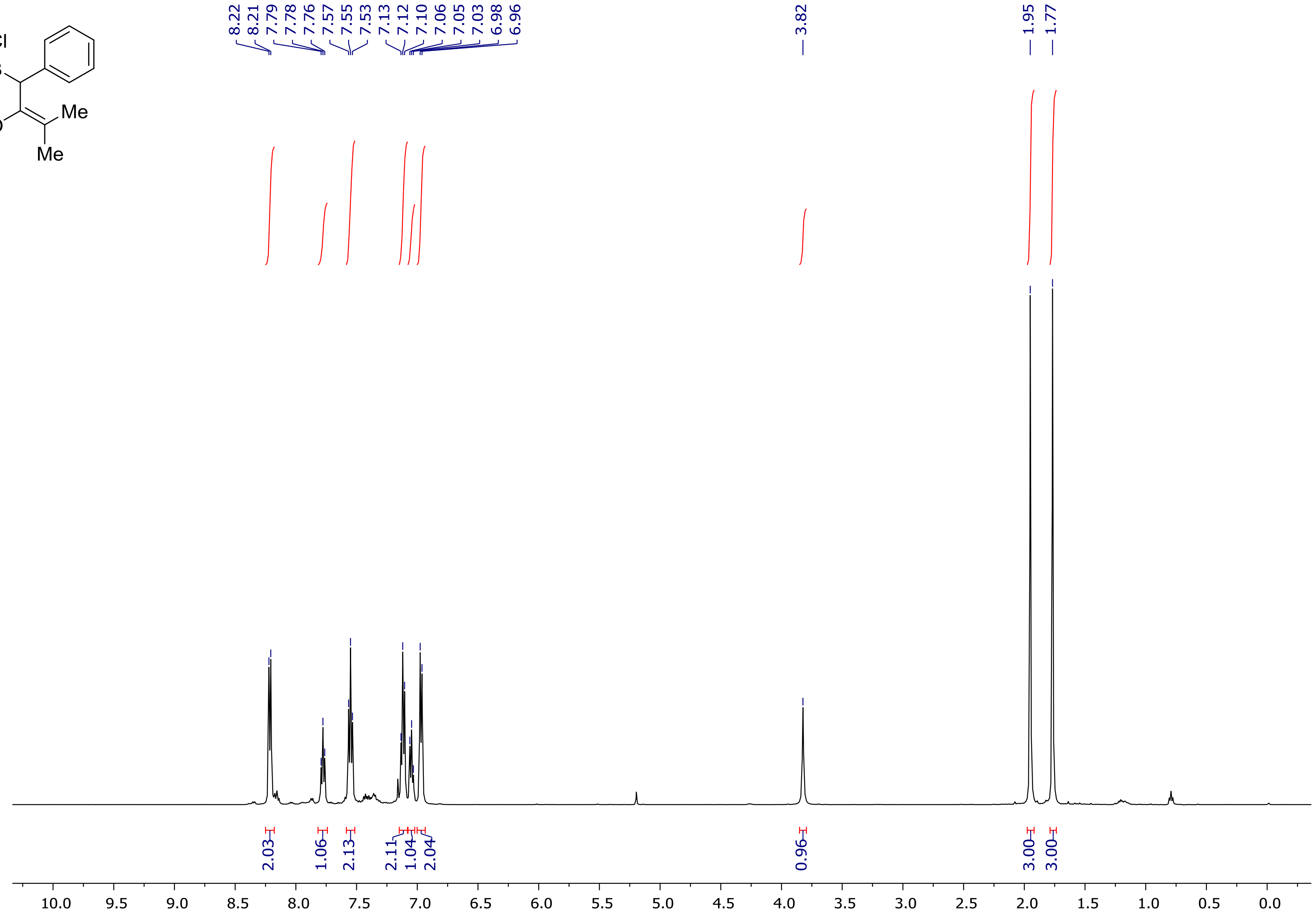

Figure S47 ^{13}C NMR (101 MHz, $CDCl_3$, 298 K) spectrum of 2,3-dichloro-2,6-diphenyl-4-(propan-2-ylidene)-3,4-dihydro-2H-1,5,2-dioxaborinin-1-ium-2-uide (**4a**).

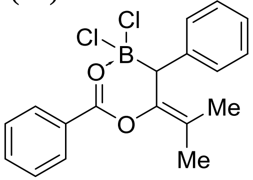

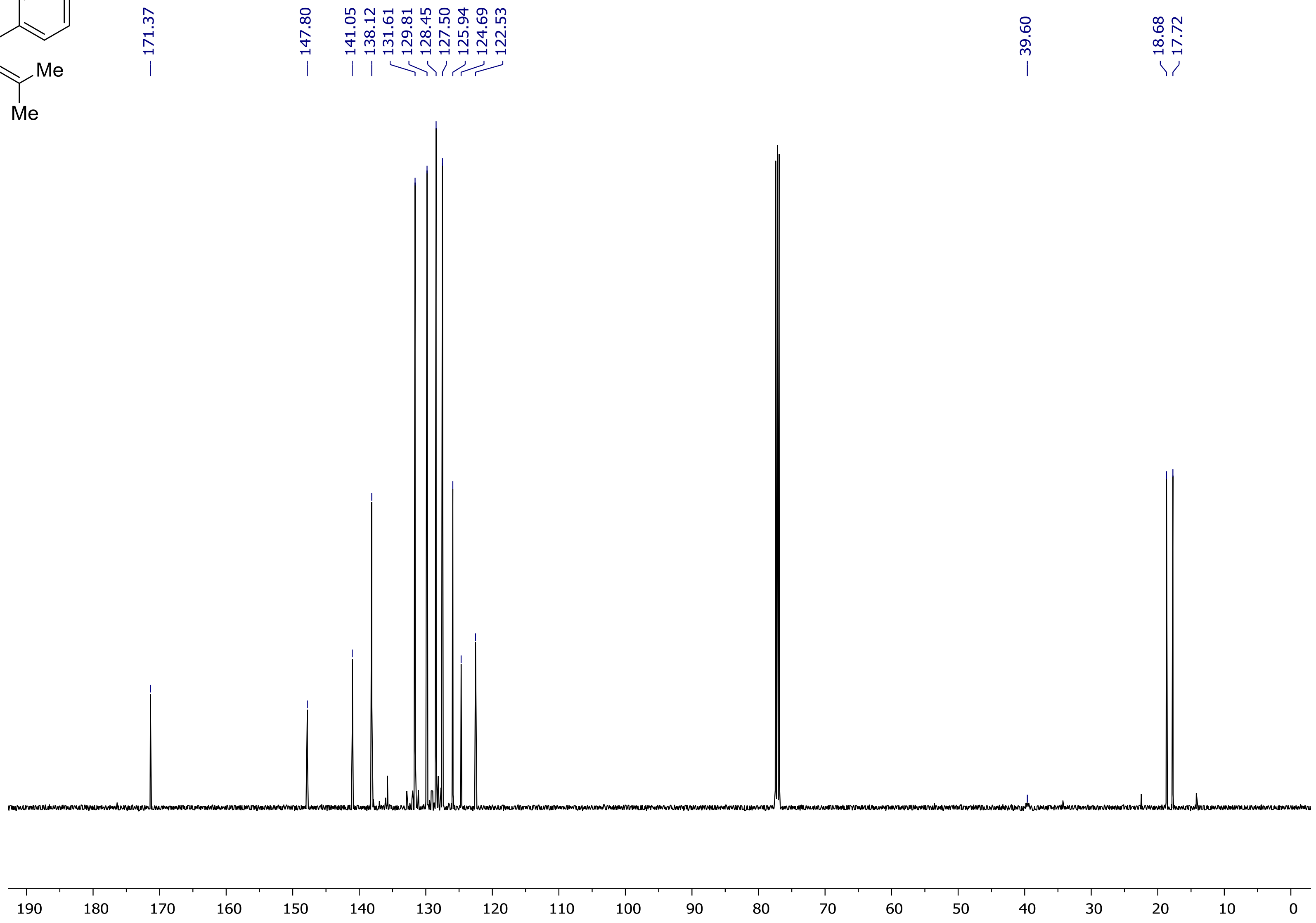

Figure S48 ^{11}B NMR (160 MHz, $CDCl_3$, 298 K) spectrum of 2,3-dichloro-2,6-diphenyl-4-(propan-2-ylidene)-3,4-dihydro-2H-1,5,2-dioxaborinin-1-ium-2-uide (**4a**).

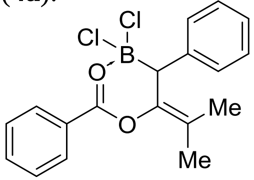

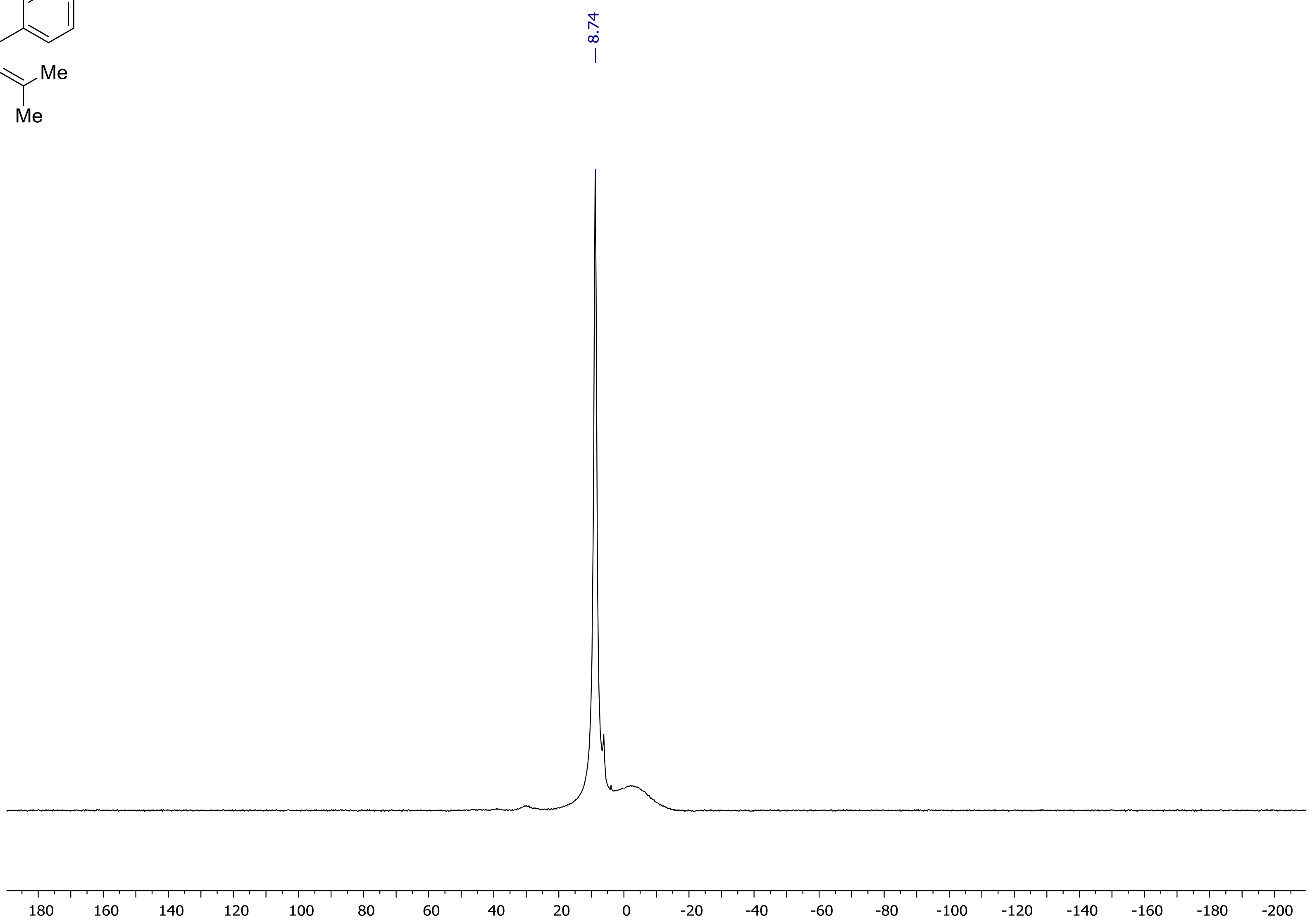

Figure S49 HSQC (^1H-^{13}C) spectrum of 2,3-dichloro-2,6-diphenyl-4-(propan-2-ylidene)-3,4-dihydro-2H-1,5,2-dioxaborinin-1-ium-2-uide (**4a**).

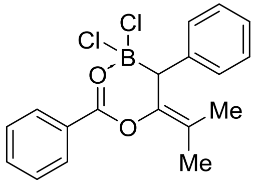

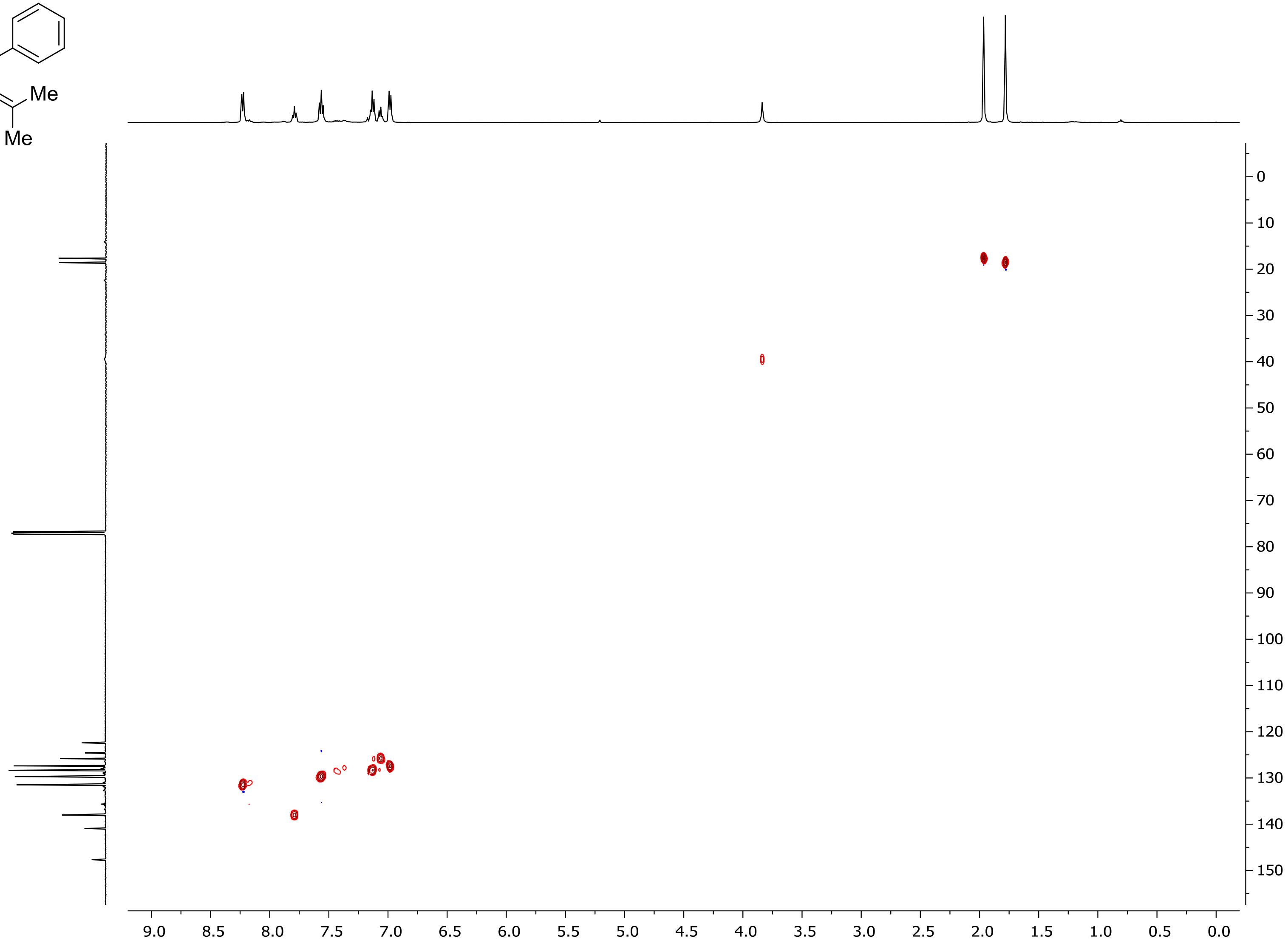

Figure S50 HMBC (^{1}H-^{13}C) spectrum of 2,3-dichloro-2,6-diphenyl-4-(propan-2-ylidene)-3,4-dihydro-2H-1,5,2-dioxaborinin-1-ium-2-uide (**4a**).

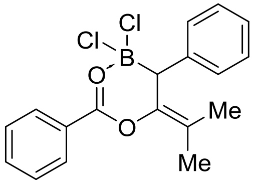

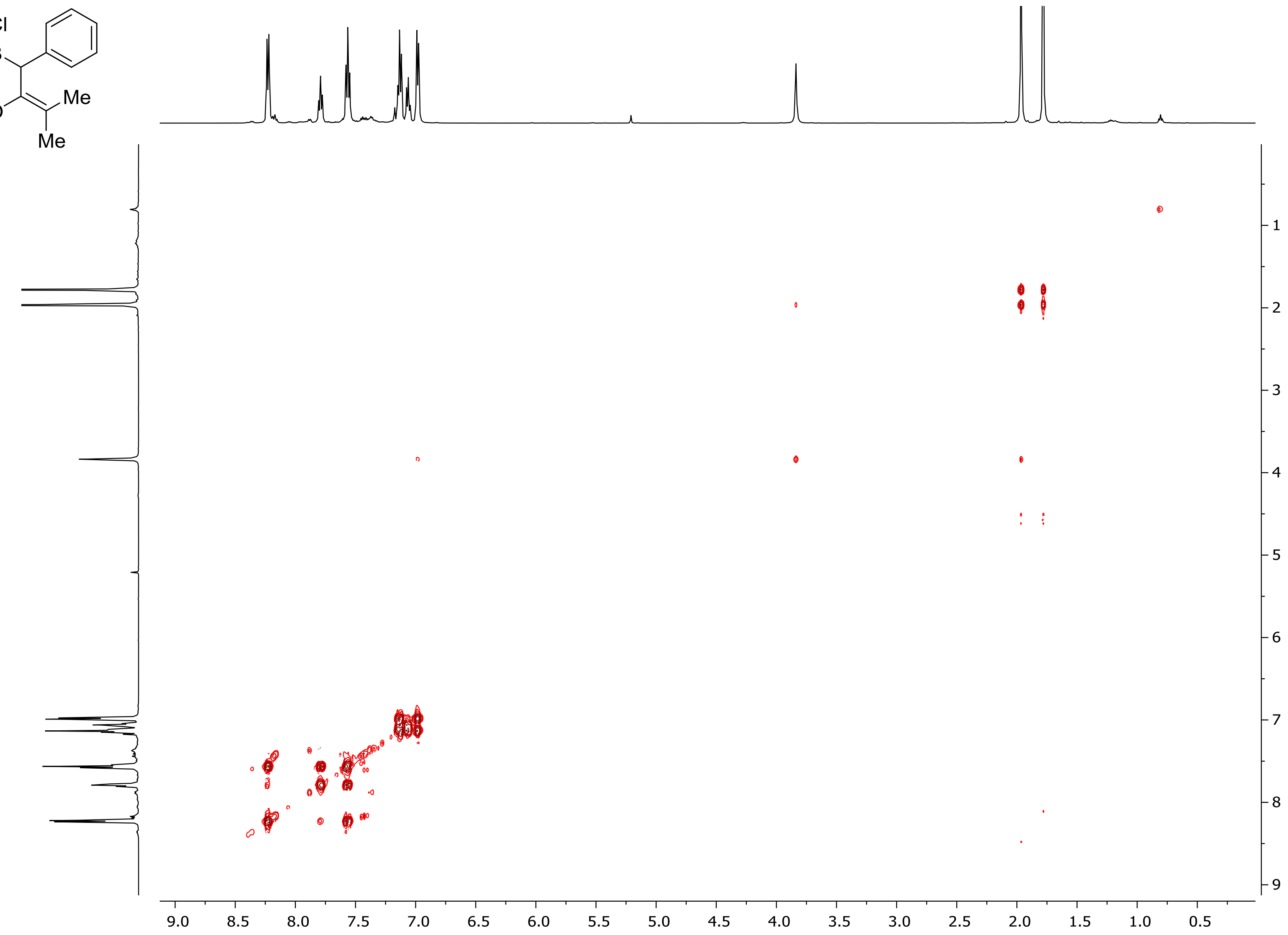

Figure S51 ^{1}H NMR (400 MHz, $CDCl_3$, 298 K) spectrum of 2,2-dichloro-3-phenyl-4-(propan-2-ylidene)-6-(4-(p-tolyl))-3,4-dihydro-2H-1,5,2-dioxaborinin-1-ium-2-uide (**4b**).

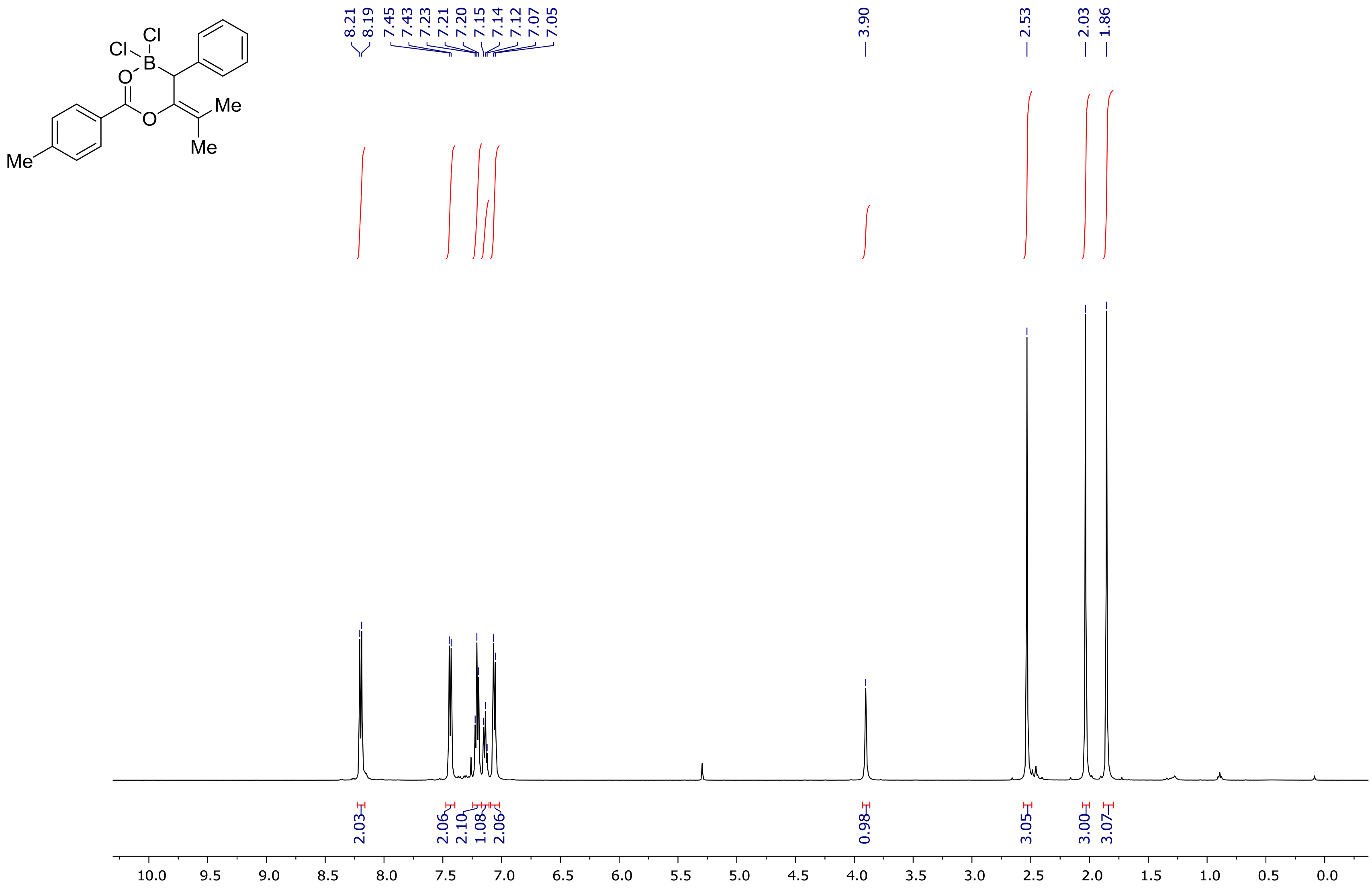

Figure S52 ^{13}C NMR (101 MHz, $CDCl_3$, 298 K) spectrum of 2,2-dichloro-3-phenyl-4-(propan-2-ylidene)-6-(4-(p-tolyl))-3,4-dihydro-2H-1,5,2-dioxaborinin-1-ium-2-uide (**4b**).

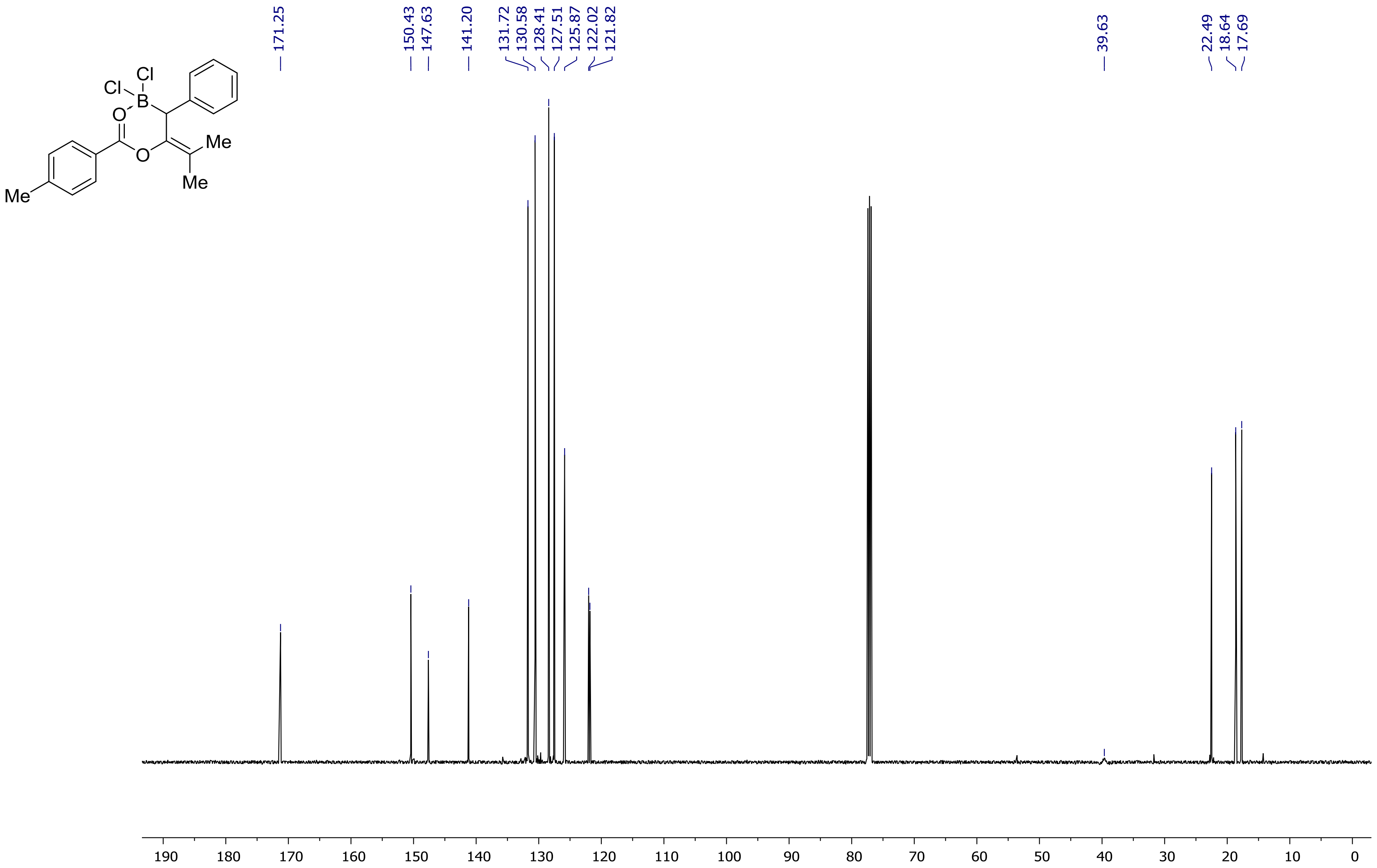

Figure S53 ^{11}B NMR (160 MHz, $CDCl_3$, 298 K) spectrum of 2,2-dichloro-3-phenyl-4-(propan-2-ylidene)-6-(4-(p-tolyl))-3,4-dihydro-2H-1,5,2-dioxaborinin-1-ium-2-uide (**4b**).

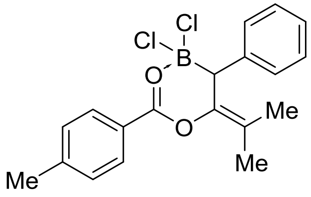

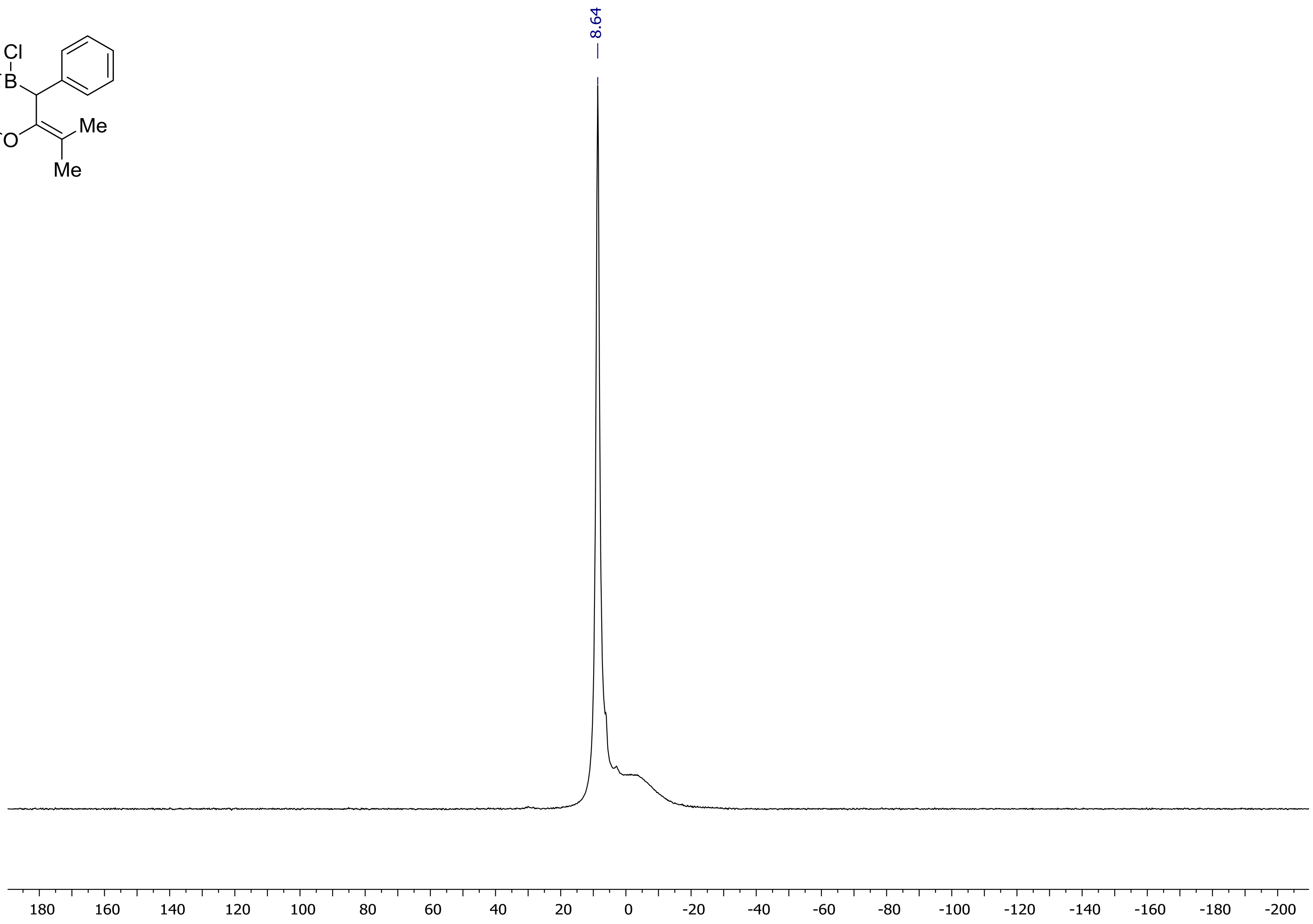

Figure S54 HSQC (^{1}H-^{13}C) spectrum of 2,2-dichloro-3-phenyl-4-(propan-2-ylidene)-6-(4-(p-tolyl))-3,4-dihydro-2H-1,5,2-dioxaborinin-1-ium-2-uide (**4b**).

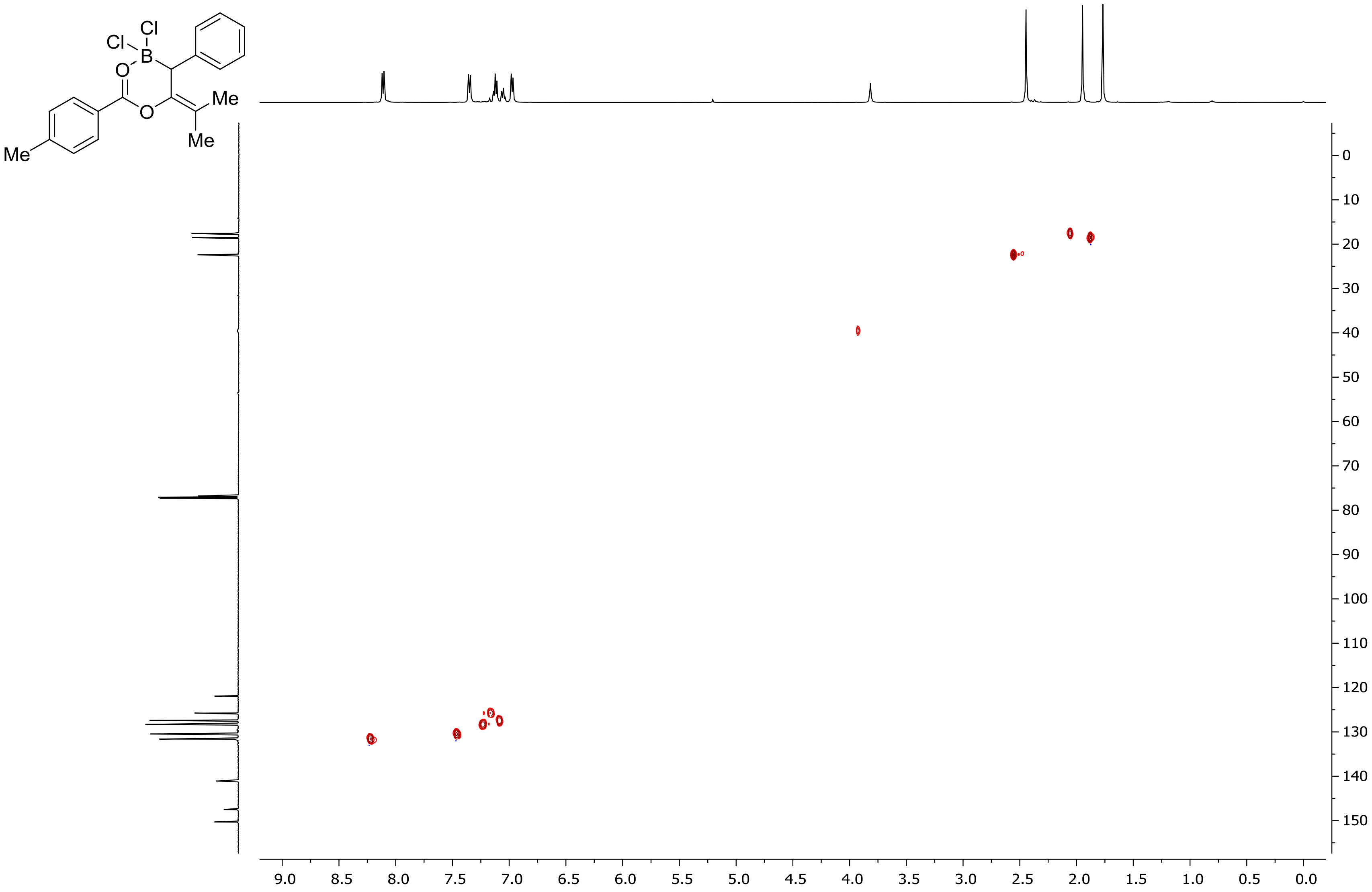

Figure S55 HMBC (^{1}H-^{13}C) spectrum of 2,2-dichloro-3-phenyl-4-(propan-2-ylidene)-6-(4-(p-tolyl))-3,4-dihydro-2H-1,5,2-dioxaborinin-1-ium-2-uide (**4b**).

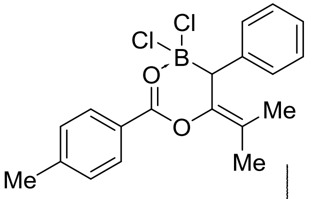

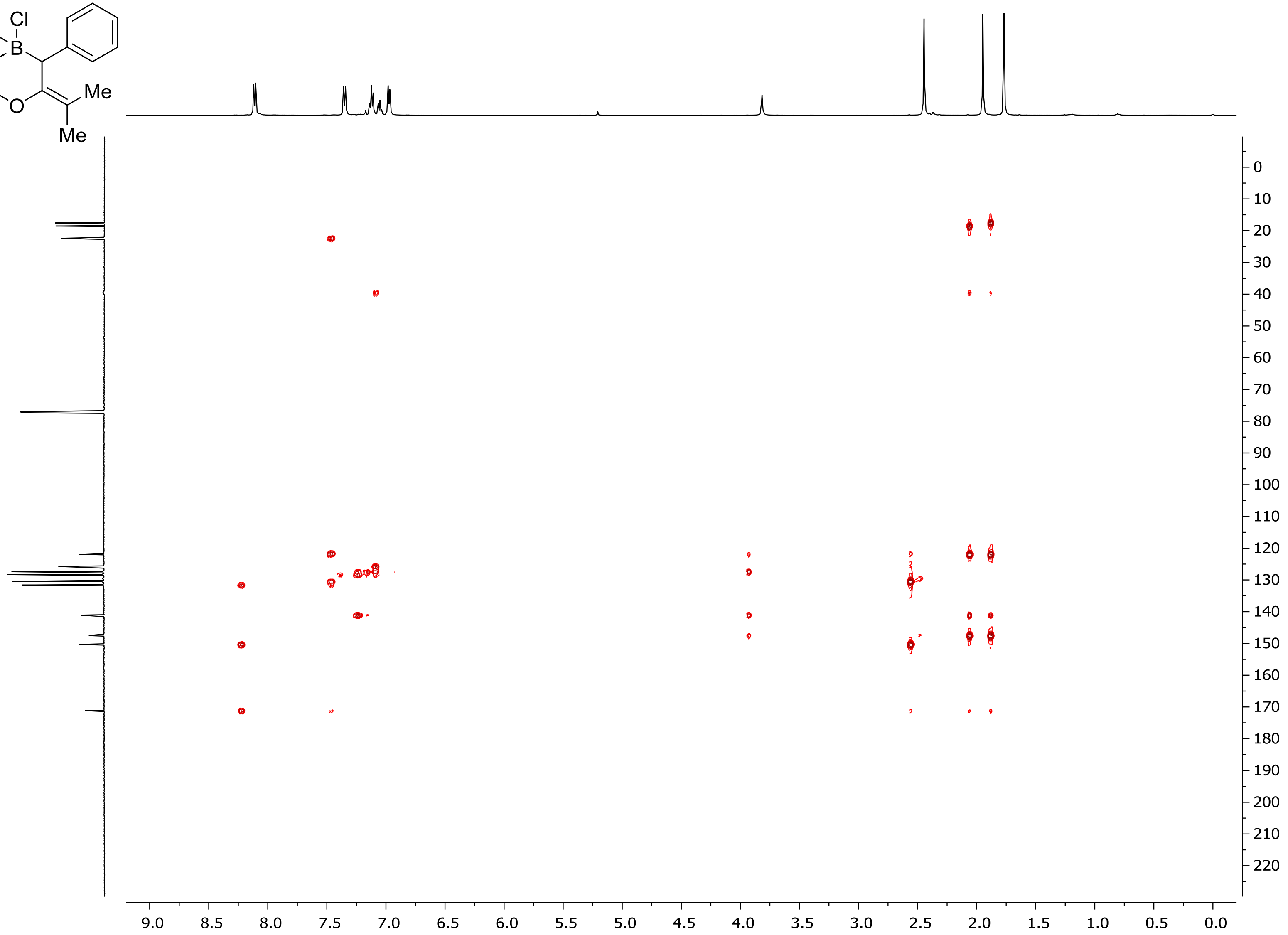

Figure S56 ^{1}H NMR (400 MHz, $CDCl_3$, 298 K) spectrum of 2,2-dichloro-6-(4-fluorophenyl)-3-phenyl-4-(propan-2-ylidene)-3,4-dihydro-2H-1,5,2-dioxaborinin-1-ium-2-uide (**4c**).

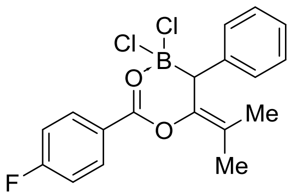

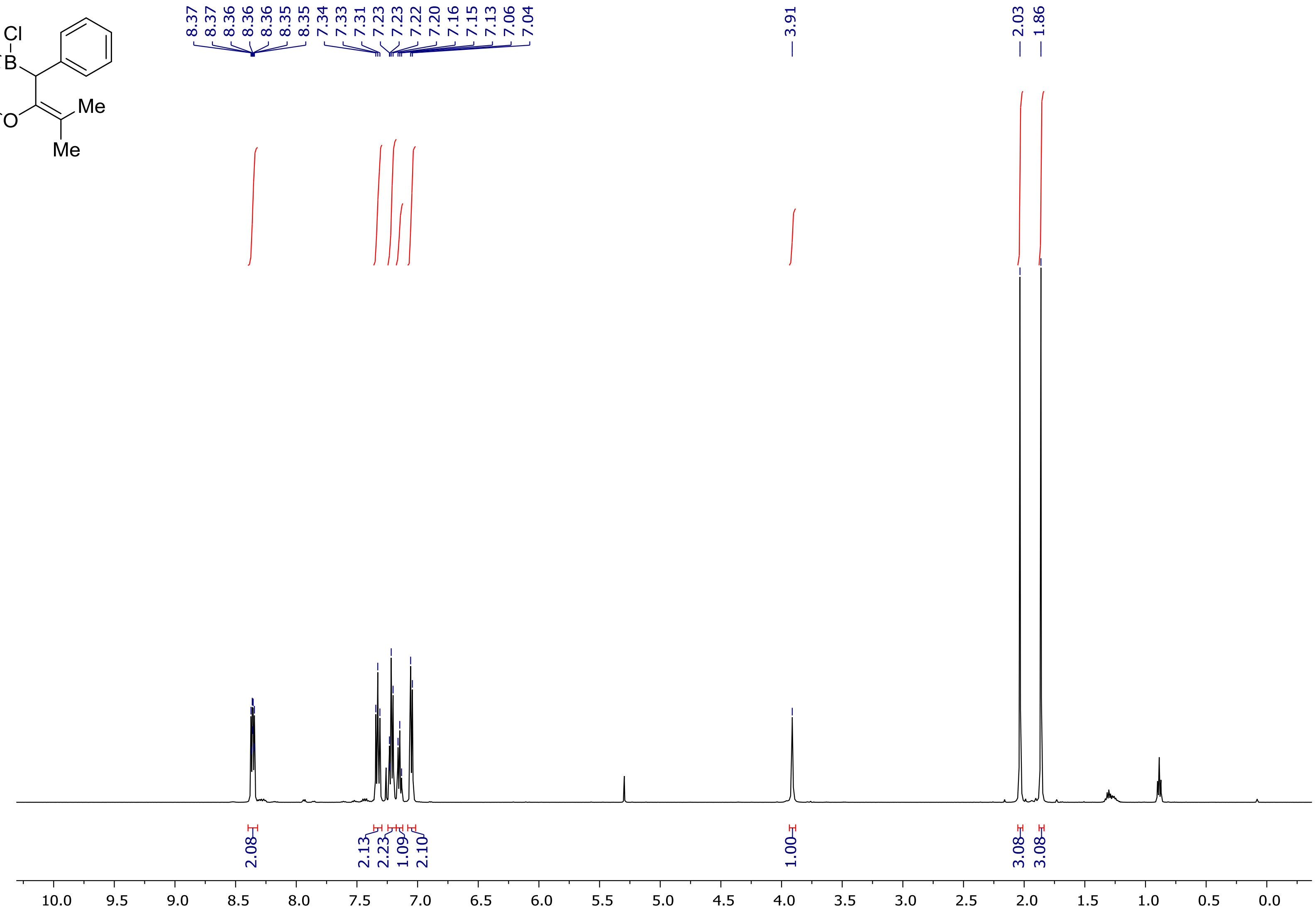

Figure S57 ^{13}C NMR (101 MHz, $CDCl_3$, 298 K) spectrum of 2,2-dichloro-6-(4-fluorophenyl)-3-phenyl-4-(propan-2-ylidene)-3,4-dihydro-2H-1,5,2-dioxaborinin-1-ium-2-uide (**4c**).

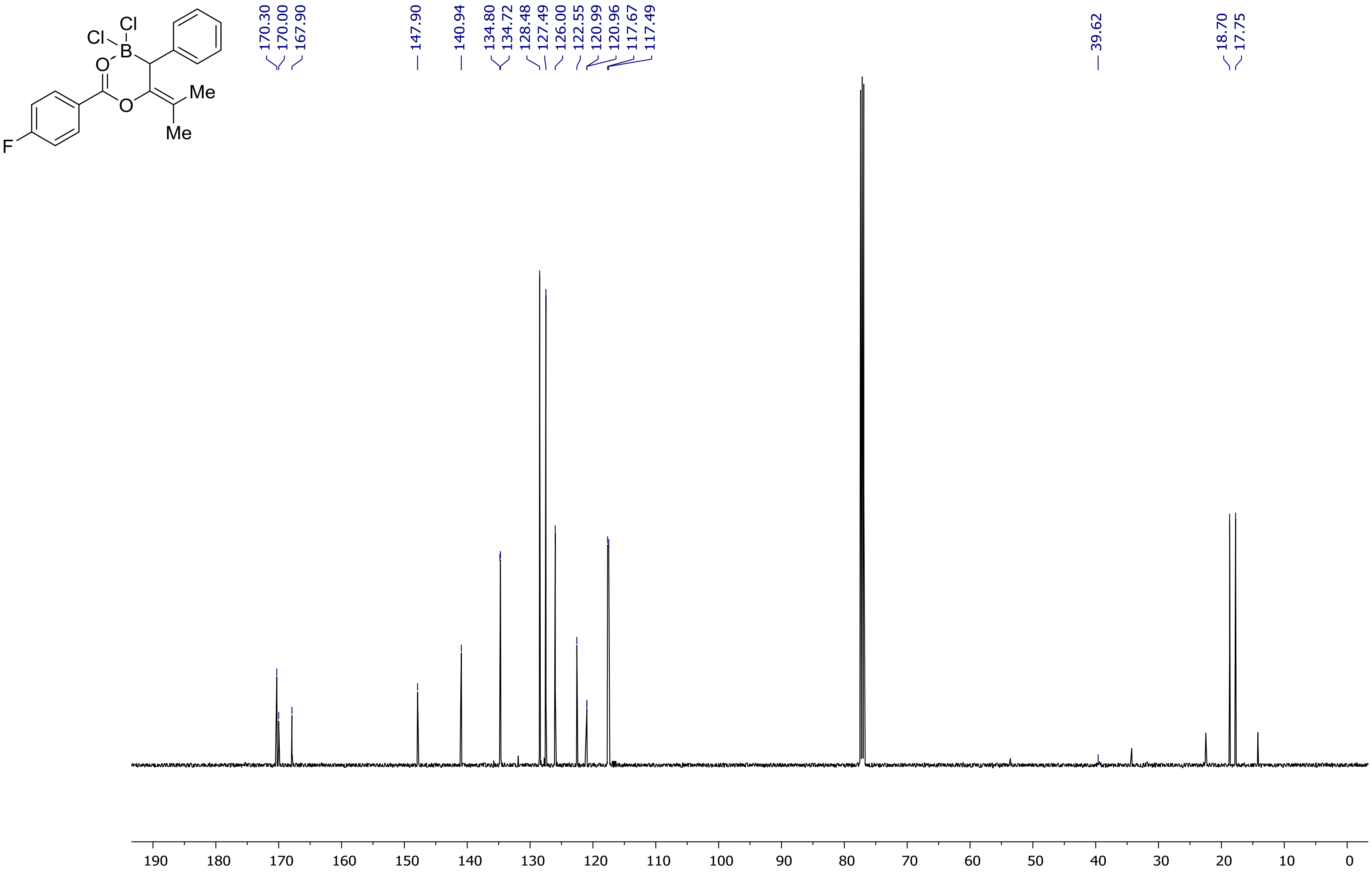

Figure S58 ^{11}B NMR (160 MHz, $CDCl_3$, 298 K) spectrum of 2,2-dichloro-6-(4-fluorophenyl)-3-phenyl-4-(propan-2-ylidene)-3,4-dihydro-2H-1,5,2-dioxaborinin-1-ium-2-uide (**4c**).

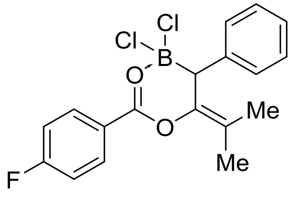

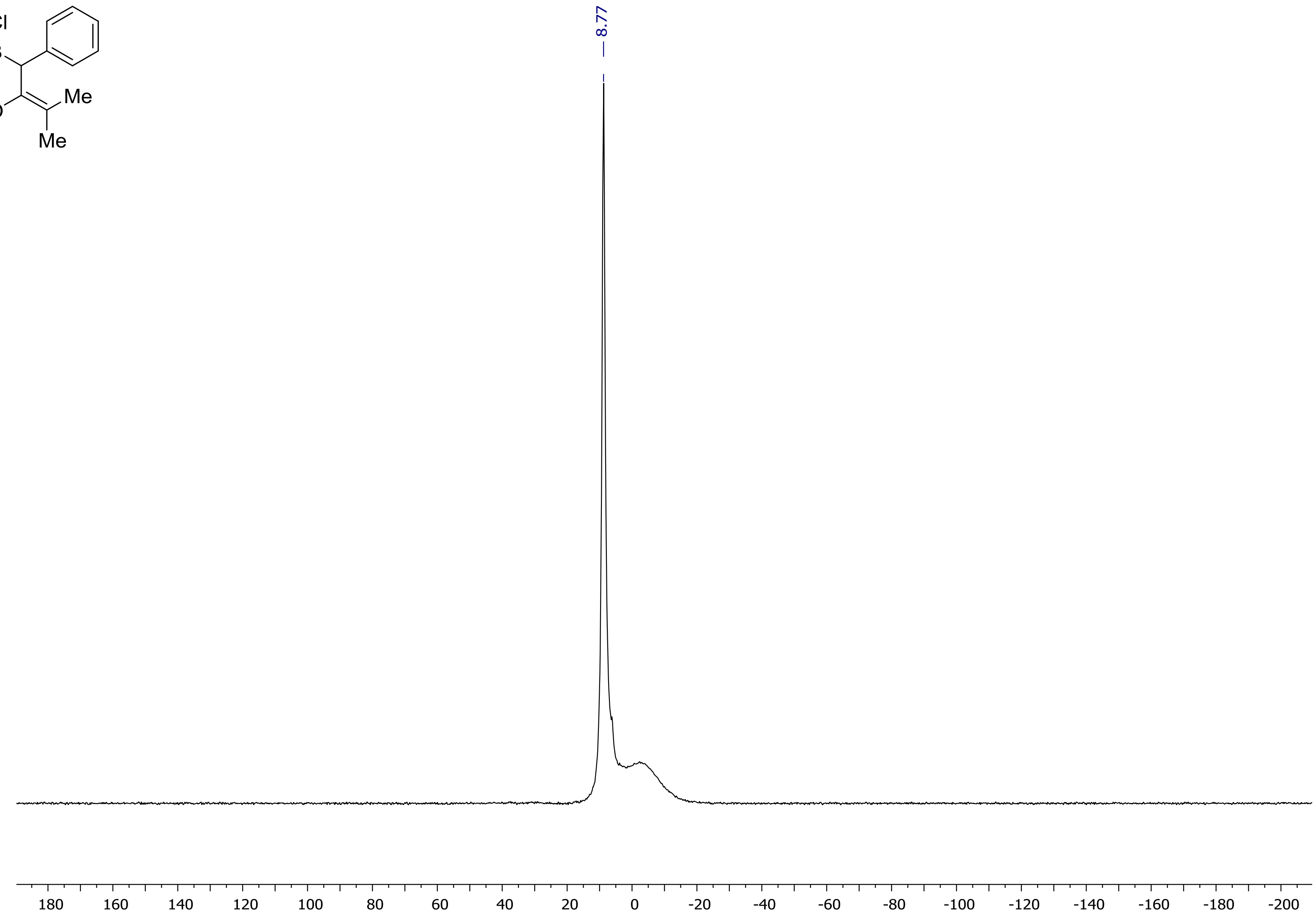

Figure S59 ^{19}F NMR (471 MHz, $CDCl_3$, 298 K) spectrum of 2,2-dichloro-6-(4-fluorophenyl)-3-phenyl-4-(propan-2-ylidene)-3,4-dihydro-2H-1,5,2-dioxaborinin-1-ium-2-uide (**4c**).

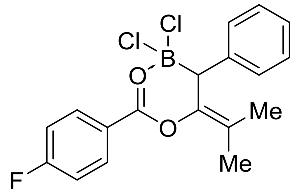

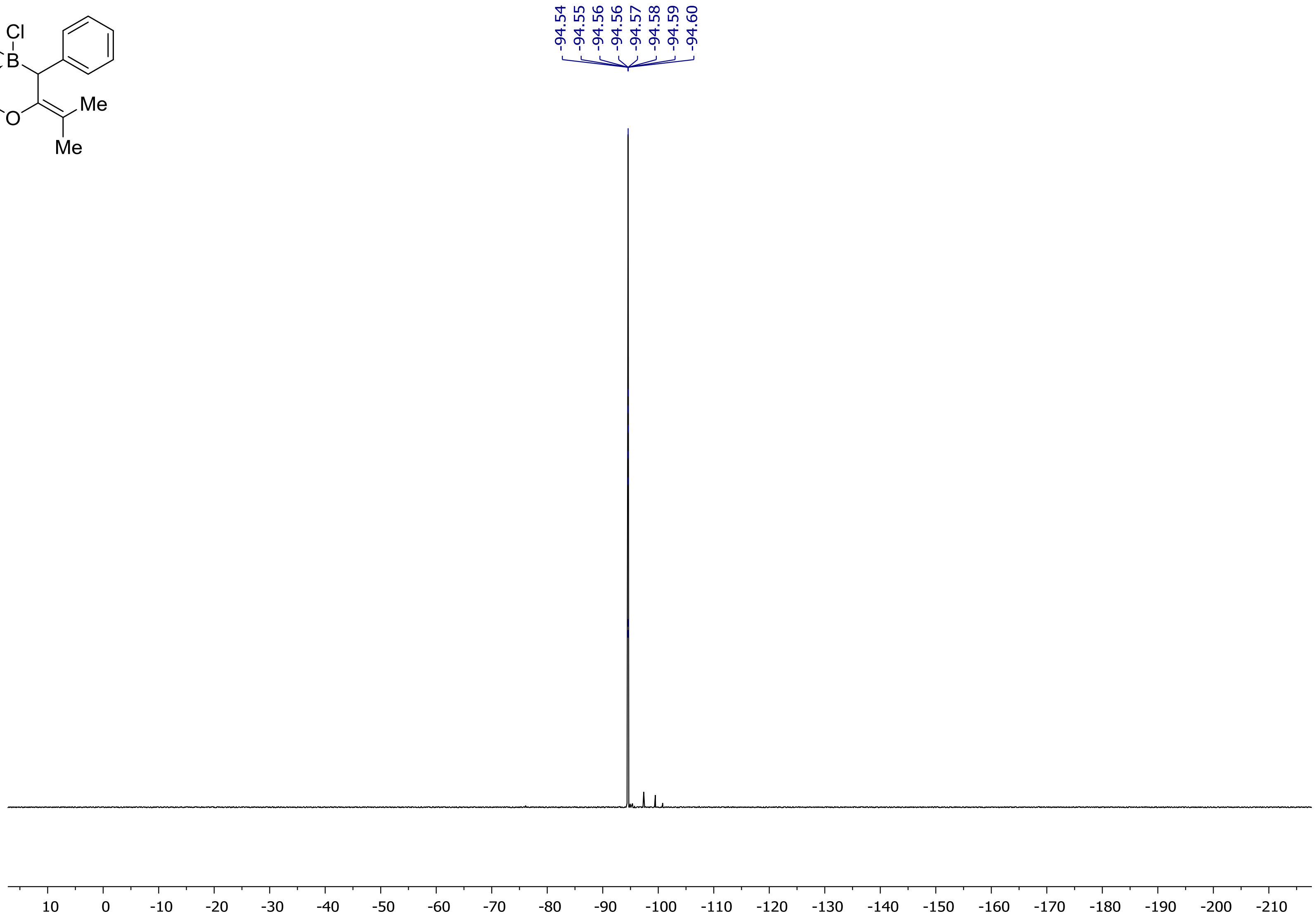

Figure S60 HSQC (^{1}H-^{13}C) spectrum of 2,2-dichloro-6-(4-fluorophenyl)-3-phenyl-4-(propan-2-ylidene)-3,4-dihydro-2H-1,5,2-dioxaborinin-1-ium-2-uide (**4c**).

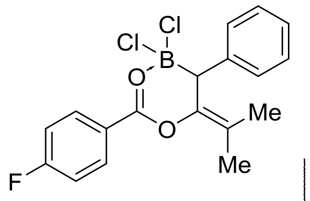

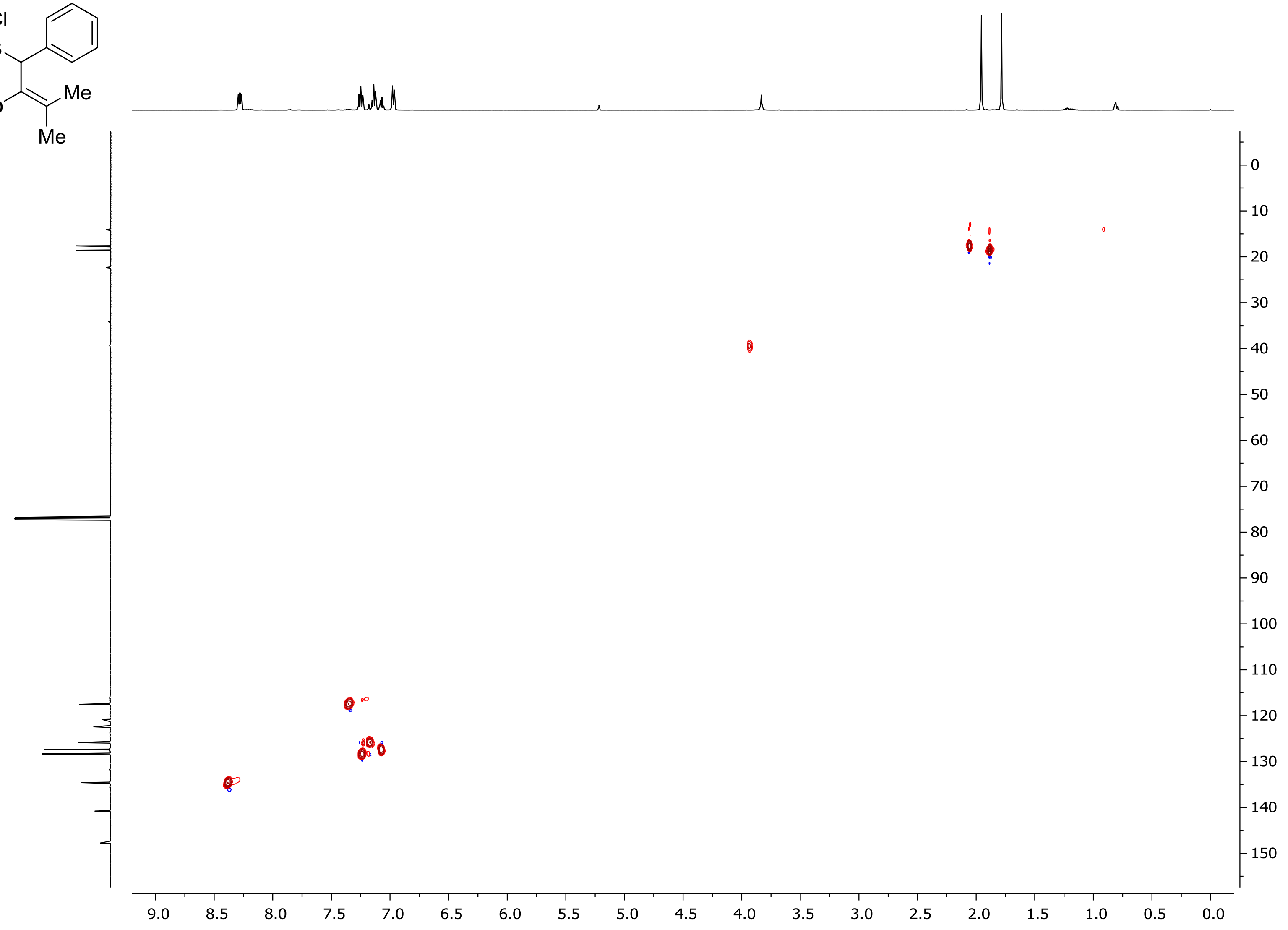

Figure S61 HMBC (^1H-^{13}C) spectrum of 2,2-dichloro-6-(4-fluorophenyl)-3-phenyl-4-(propan-2-ylidene)-3,4-dihydro-2H-1,5,2-dioxaborinin-1-ium-2-uide (**4c**).

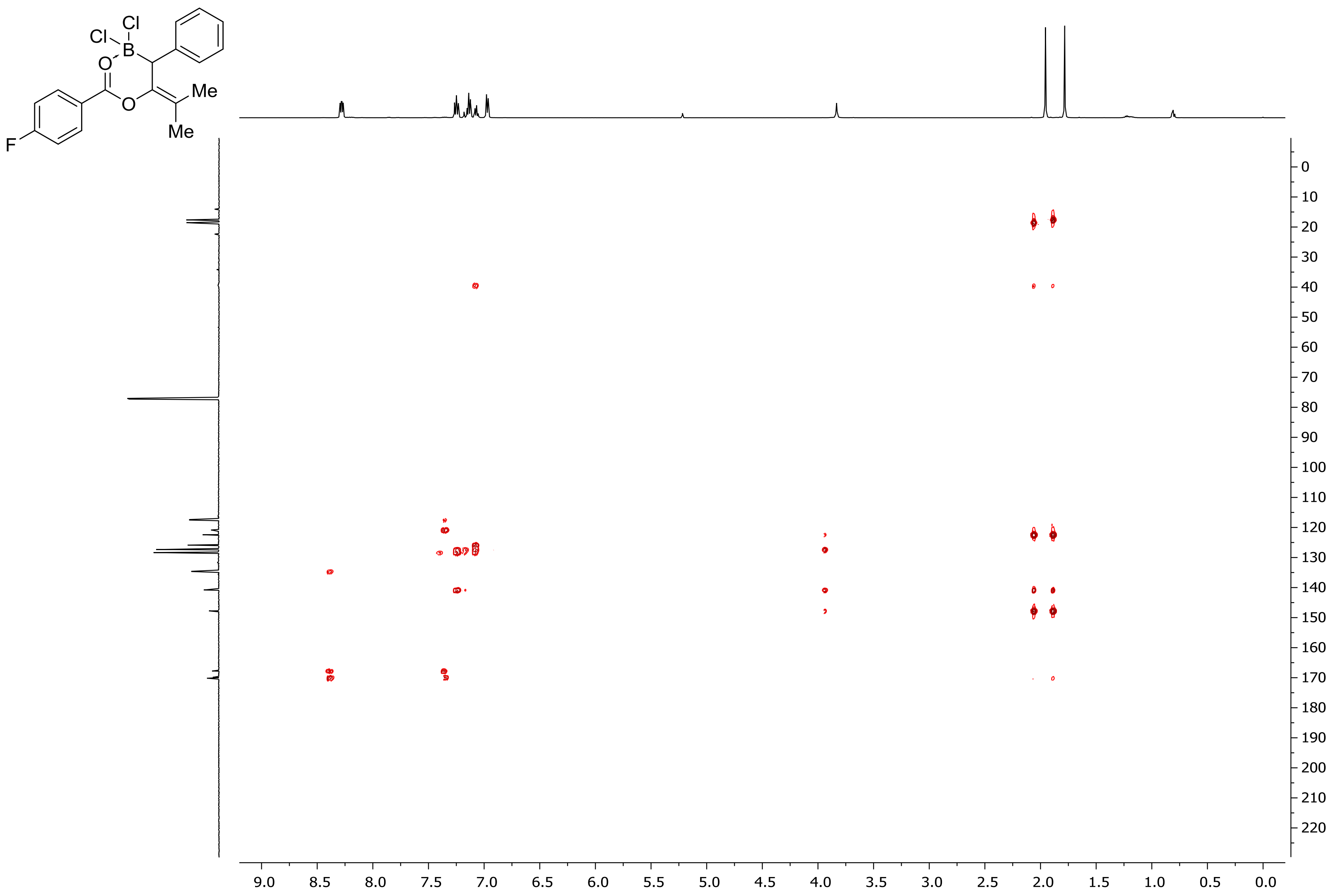

Figure S62 Stacked ^{1}H NMR spectra of the *in situ* reaction between **5** and $PhBCl_2$ over time.

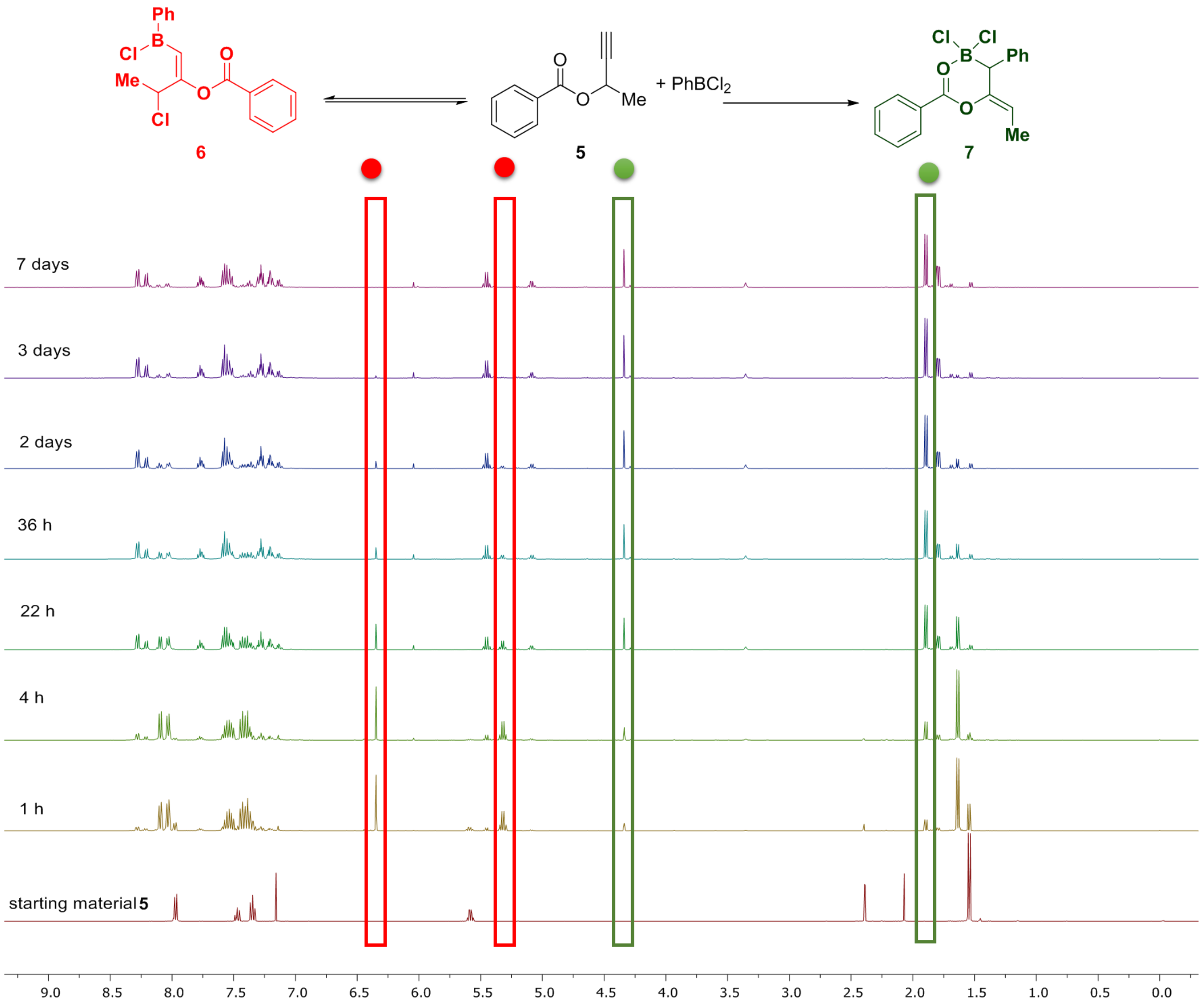

Figure S63 Stacked ^{11}B NMR spectra of the *in situ* reaction between **5** and $PhBCl_2$ over time.

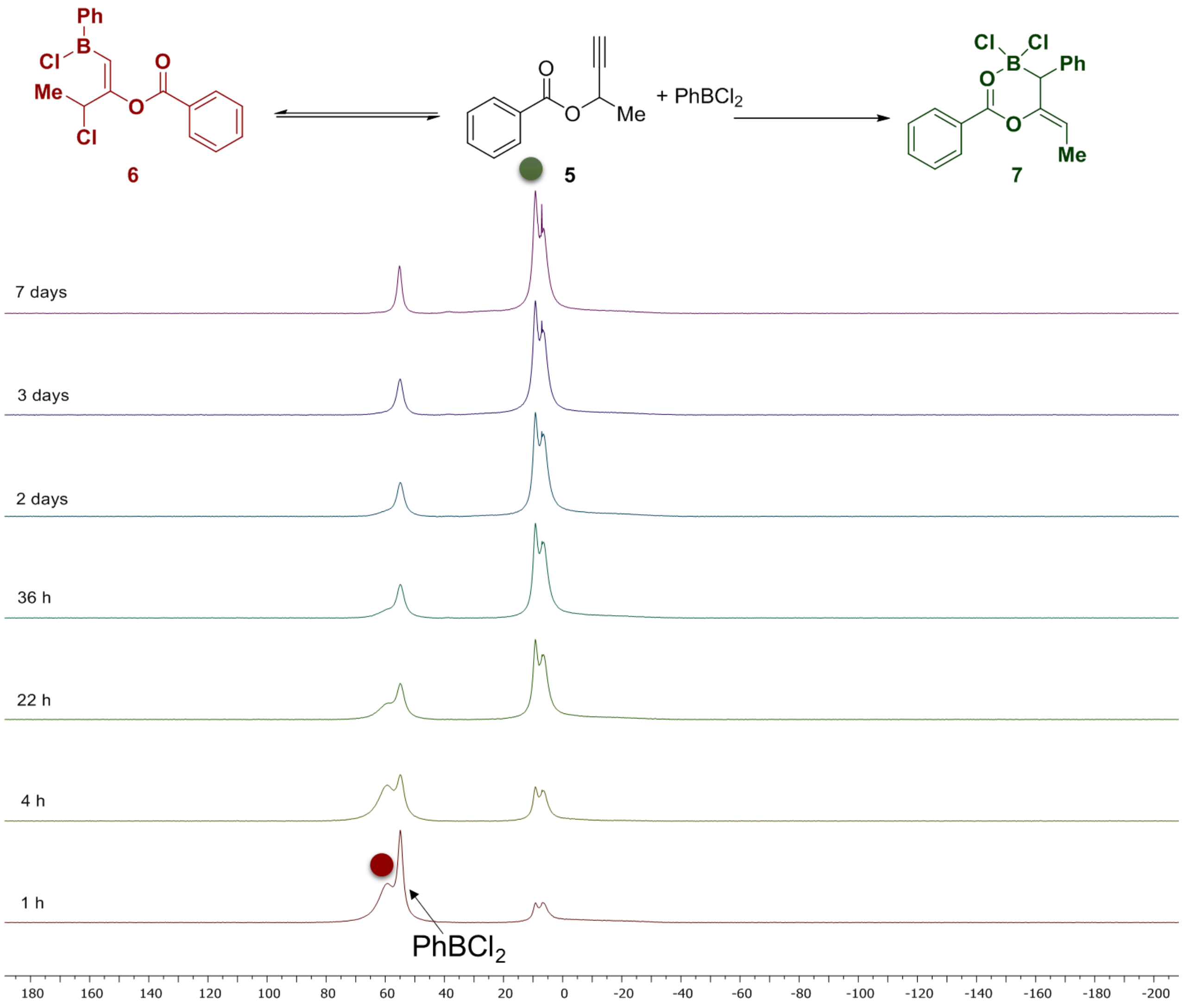

3 Computational

3.1 Computational settings

All calculations were peformed using NWChem 6.6[8] (www.nwchem.org) and the Def2-tzvp basis set on all atoms. To probe the reaction thermochemistry, we have used two hybrid meta-GGA functionals, M06-2X[9] and PW6B95[10], which have been shown to perform well in comprehensive benchmarking studies including main group reaction chemistry and non-covalent interactions such as London dispersion dominated reference cases[11,12]. For each functional, Grimme's "D3"[13] dispersion correction has been used: M06-2X-D3 and PW6B95-D3.

Initial reactants were fully (geometry) optimized in a variety of trial geometries to establish the lowest energy configurations. Product geometries were also trialed in different geometries based on chemical intuition and also using the molecular geometries isolated from the crystal structures. Hypothetical 1,3-haloboration (R^2 = Me) and 1,1-carboboration (R^2 = H) products were alchemically generated from the 1,3-haloboration (R^2 = H) and 1,1-carboboration (R^2 = Me) configurations respectively and optimized from a variety of initial configurations. All energetics discussed in the main paper refer to the M062X-D3 data.

For the energy level diagram, the geometries identified in the gas phase were used as starting points for (distance) constrained optimization calculations to scope transition states using the python extensions of NWChem. The free energy of solvation (using COSMO, standard dielectric settings for chloroform) was determined for each intermediate and transition state (fully optimized geometries) and the vibrational frequencies determined to compute the free energies at 298K and also to verify minima have no negative frequencies and that transition states had one negative frequency. As another check on the robustness of the data, single point energies were carried out using the def2-tzvp geometries at the def2-qzvp basis level for selected barriers and it was found that the def2-qzvp free energy differences were within 0.25 kcal mol^{-1} of the def2-tzvp energies. Again, all data refers to that computed with M062X-D3.

3.2 Reaction thermochemistry

3.2.1 Gas Phase

Initial calculations focused on the gas phase thermochemistry of the reactants and products. Table S1 shows the reaction enthalpies of the 1,3-haloboration and 1,1-carboboration reactions for R^2 = Me and R^2 = H under gas phase conditions.

	Reaction energetics	
	M062X-D3/kcal mol^{-1}	PW6B95-D3/kJ mol^{-1}
1,3-haloboration		
R^2=H	-9.87	-9.27
R^2=Me	-8.83	-9.57
1,1-carboboration		
R^2=H	-43.02	-41.48
R^2=Me	-44.35	-42.20

Table S1: Calculated reaction energies reported in kcal mol^{-1} for the 1,1-carboboration and 1,3-haloboration reactions using two density functional approaches (gas phase).

Table S1 emphasizes the large discrepancy between the 1,1-carboboration and 1,3-haloboration reactions, where the haloboration product is greatly favored over carboboration, due to the formation, presumably, of a condensed heterocycle in the carboboration product rather than a more linear conformer for the haloboration case. Also notable is the quantitative similarity in the reaction energies for R^2=H and R^2=Me, for both reactions that suggests, in the absence of any kinetic considerations, 1,1-carboboration and 1,3-haloboration products are viable for *both* R^2=H and R^2=Me but the thermodynamically most favorable reaction would be expected to the 1,1-carboboration.

3.2.2 Free energies of reaction in solution

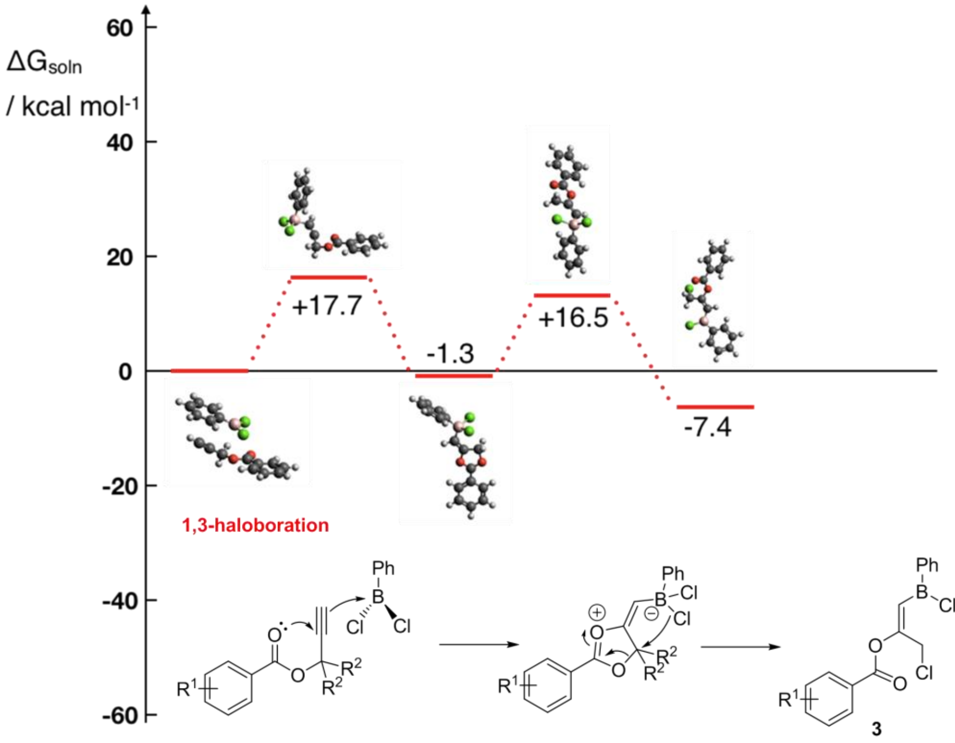

Figure S64: Calculated reaction free energy differences reported in kcal mol^{-1} for the 1,3-haloboration reaction R^2=H. Free energies are ZPE corrected at 298K in the presence of implicit solvent.

1,3, haloboration reaction	ΔG_{soln} / kcal mol^{-1}	ΔH_{soln} / kcal mol^{-1}
adduct TS*	+17.74	+18.69
dioxolium adduct	-19.00	-20.53
Chloride TS*	+17.70	+18.11
Product **3**	-23.87	-24.09

Table S2: Calculated reaction free energy and enthalpy differences reported in kcal mol^{-1} for the 1,3-haloboration reaction R^2=H. Free energies are ZPE corrected at 298 K in the presence of implicit solvent.

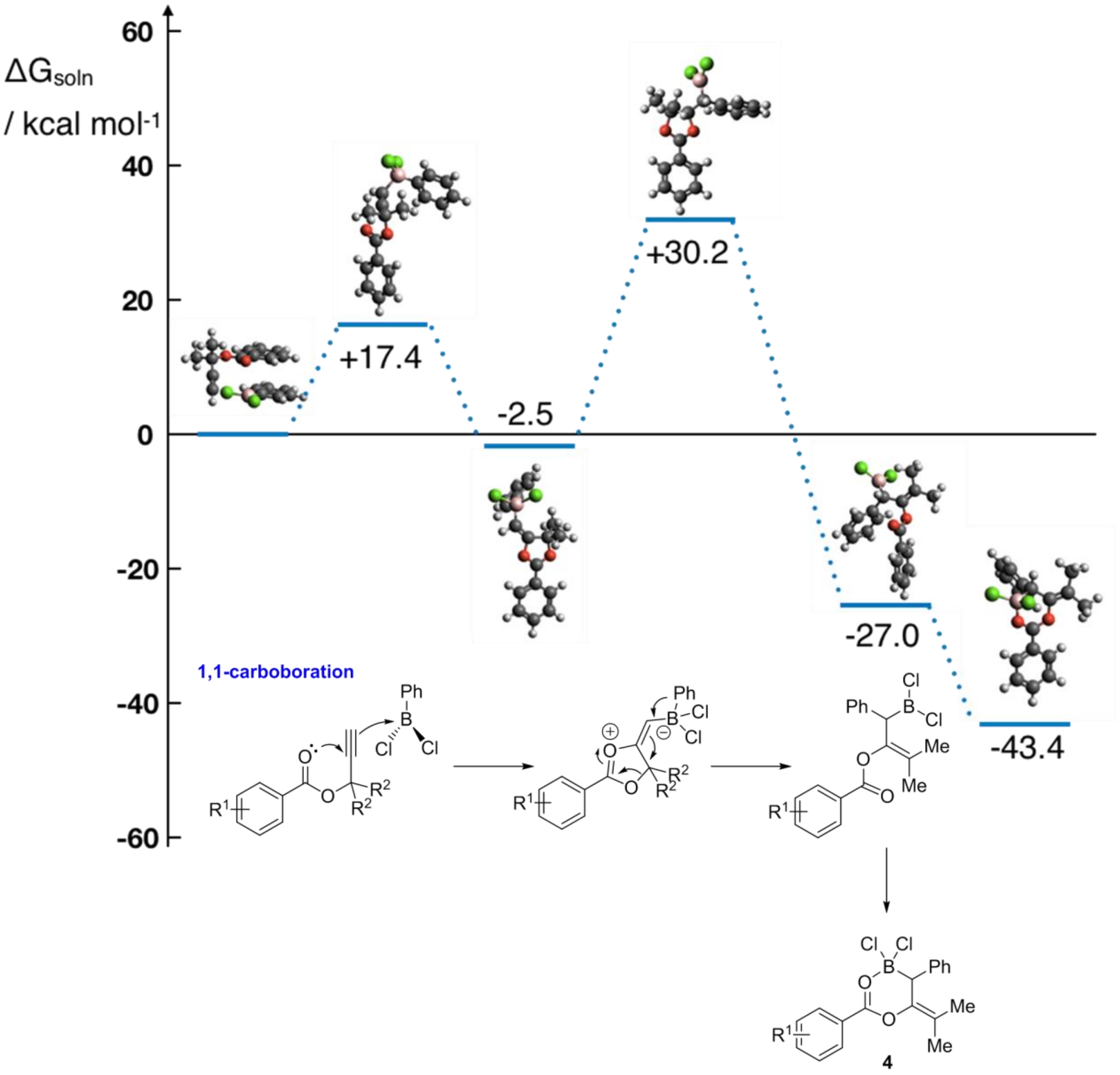

Figure S65: Calculated reaction free energy differences reported in kcal mol^{-1} for the 1,1-carboboration reaction R^2=Me. Free energies are ZPE corrected at 298K in the presence of implicit solvent.

1,1-carboboration reaction	ΔG_{soln} / kcal mol^{-1}	ΔH_{soln} / kcal mol^{-1}
Adduct TS*	+17.38	+18.01
dioxolium adduct	-19.88	-21.80
Ph migration TS*	+32.68	+34.11
Pincer intermediate	-57.22	-57.11
Product **4**	-16.31	-16.15

Table S3: Calculated reaction free energy and enthalpy differences reported in kcal mol^{-1} for the 1,1-carboboration reaction R^2=Me. Free energies are ZPE corrected at 298K in the presence of implicit solvent.

3.2.3 Comparing the 1,3-haloboration of energies of 3 (R^2=H), 3 (R^2=Me) and 6

The above data shows striking agreement between free energies and enthalpies and additionally, solvation energies were found to be similar for all species. Hence, the formation energies of **3** (R^2=H), **3** (R^2=Me) and **6** were computed in the gas phase along with the intermediates to assess forward and backward reaction for the 1,3-haloboration reaction. The free energy differences are reported, ZPE corrected at 298 K.

1,3-haloboration reaction R^2=H	ΔG_{gas} / kcal mol^{-1}
Reverse reaction (**3** to **TS**)	+28.88
Forward reaction (adduct to **TS**)	+12.75
Overall reaction energy	-16.12

Table S4: Calculated reaction free energy differences reported in kcal mol^{-1} for the 1,3-haloboration reaction R^2=H (product **3**), gas phase at 298 K.

1,3-haloboration reaction R^2=Me	ΔG_{gas} / kcal mol^{-1}
Reverse reaction (**3** to **TS**)	+23.29
Forward reaction (adduct to **TS**)	+13.36
Overall reaction energy	-9.81

Table S5: Calculated reaction free energy differences reported in kcal mol^{-1} for the 1,3-haloboration reaction R^2=Me (Me analogue of **3**), gas phase at 298 K.

1,3-haloboration reaction R^2=Me,H	ΔG_{gas} / kcal mol^{-1}
Reverse reaction (**6** to **TS**)	+25.78
Forward reaction (adduct to **TS**)	+15.05
Overall reaction energy	-10.72

Table S6: Calculated reaction free energy differences reported in kcal mol^{-1} for the 1,3-haloboration reaction R^2=Me, H (mono-methylated product **6**), gas phase at 298 K.

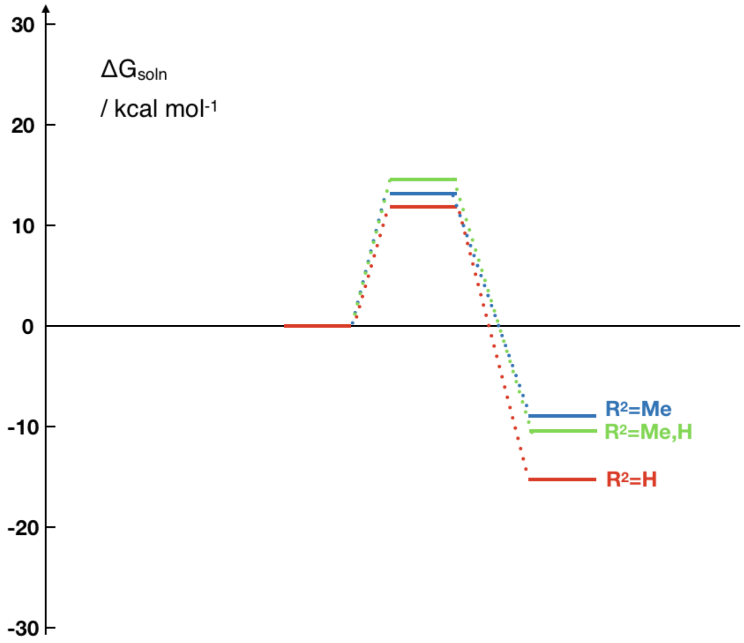

Figure S66: Calculated reaction free energy differences reported in kcal mol^{-1} for the 1,3-haloboration reaction for R^2=H, R^2=Me and R^2=Me,H. Free energies are ZPE corrected at 298K.

3.2.4 Comparing the 1,1-carboboration transition state energies of R^2=H, R^2=Me and R^2=Me,H

Additionally, the transition state barrier for the 1,1-carboboration associated with the phenyl migration step has been calculated for **4** (R^2=Me), hypothetical **4** (R^2=H), and **7** (R^2=Me, H) species. In the table below, the numbers refer to barriers calculated in the gas phase at 0 K.

1,1-carboboration reaction	ΔE_{gas} / kcal mol^{-1}
Phenyl migration barrier for R^2= H	+37.20
Phenyl migration barrier for R^2= Me	+28.23
Phenyl migration barrier for R^2= Me, H	+29.19

Table S7: Calculated reaction barrier heights for phenyl migration reported in kcal mol^{-1} for the 1,1-carboboration reaction, gas phase.

The barriers clearly illustrate that phenyl migration is most facile for R^2=Me, the barrier is larger for R^2=Me, H but comparable with the barrier for R^2=Me but the barrier for migration of the phenyl for R^2=H is notably higher by more than 7 kcal mol^{-1} and is the least plausible process. Phenyl migration clearly occurs in the formation of products **4** and **7** but does not occur for R^2=H, where any intermediate species **I** preferentially form product **3**.

3.3 Reactant and product geometries

All structures are given in XYZ format in Angstroms for the optimized M062X-D3 configurations.

3.3.1 Reactant R^2=H (gas)

C	1.15765	1.71680	0.08008
C	1.18812	2.45754	-1.10839
C	0.87192	3.80623	-1.11840
C	0.51901	4.44286	0.06571
C	0.48610	3.72882	1.25634
C	0.80314	2.37928	1.26100
B	1.49882	0.20871	0.08801
H	1.45792	1.96422	-2.03430
H	0.90115	4.36324	-2.04638
H	0.27296	5.49783	0.06017
H	0.21267	4.22413	2.17941
H	0.76882	1.82461	2.19092
Cl	1.94242	-0.63942	-1.38616
Cl	1.49201	-0.72696	1.57177

H	-2.50452	3.15435	-0.66725
C	-2.29464	2.10634	-0.49261
C	-1.93560	1.28588	-1.55382
H	-1.87023	1.69242	-2.55504
C	-1.64064	-0.05107	-1.33138
H	-1.34481	-0.69457	-2.14860
C	-1.71634	-0.56718	-0.04009
C	-1.41460	-1.99394	0.26112
O	-1.52107	-2.49858	1.34431
O	-1.01142	-2.67130	-0.83138
C	-0.63460	-4.03081	-0.60946
C	-2.08052	0.25426	1.02335
H	-2.12336	-0.17211	2.01759
C	-2.36879	1.58951	0.79554
H	-2.63901	2.23353	1.62270
H	-0.67454	-4.50368	-1.58930
H	-1.35360	-4.50770	0.05746
C	0.71181	-4.13272	-0.05142
C	1.81992	-4.20345	0.39242
H	2.80275	-4.25557	0.79581

3.3.2 Reactant R^2=Me (gas)

C	1.09182	2.21367	-0.08365
C	0.89451	2.72569	-1.37203
C	0.48402	4.03454	-1.56590
C	0.26206	4.85954	-0.47004
C	0.45115	4.37262	0.81684
C	0.86125	3.06223	1.00579
B	1.53586	0.74818	0.13120
H	1.06162	2.08492	-2.22922
H	0.33789	4.41371	-2.56934
H	-0.05706	5.88380	-0.61978
H	0.27792	5.01452	1.67128

H	1.00023	2.68454	2.01147
Cl	1.83830	-0.32578	-1.22842
Cl	1.79193	0.09029	1.73600
H	-2.81435	3.36721	-0.29509
C	-2.48167	2.35480	-0.10292
C	-2.23299	1.49436	-1.16390
H	-2.37183	1.83545	-2.18173
C	-1.79077	0.20199	-0.92298
H	-1.58033	-0.47178	-1.74221
C	-1.60627	-0.23110	0.38729
C	-1.13037	-1.60936	0.70916
O	-1.02494	-2.03858	1.82462
O	-0.83172	-2.30236	-0.40148
C	-0.24019	-3.61867	-0.29127
C	-1.85698	0.63129	1.45112
H	-1.69625	0.27086	2.45917
C	-2.29280	1.92281	1.20456
H	-2.47774	2.59802	2.03031
C	0.10854	-3.97722	-1.73235
C	-1.24159	-4.61017	0.29378
C	0.99375	-3.55982	0.50664
C	2.03006	-3.54653	1.10314
H	2.94187	-3.51541	1.64928
H	0.57840	-4.95923	-1.76358
H	0.79798	-3.23953	-2.14079
H	-0.80252	-3.99234	-2.33133
H	-0.80079	-5.60692	0.28582
H	-2.13965	-4.61612	-0.32572
H	-1.50020	-4.34369	1.31435

3.3.3 1,3-haloboration: product R^2=H (gas)

H	1.14533	-0.77356	0.52589
O	-1.36363	2.30943	1.35115
B	-0.32009	-2.41860	0.09263
C	0.71410	-3.57237	-0.02145
C	0.36432	-4.91168	0.19219
C	1.30175	-5.92497	0.08224
C	2.61339	-5.62165	-0.25967
C	2.98384	-4.30353	-0.48765
C	2.04418	-3.29288	-0.36282
H	-0.65766	-5.15771	0.45192
H	1.01173	-6.95266	0.25920
H	3.34676	-6.41325	-0.34971
H	4.00386	-4.06543	-0.76036
H	2.34811	-2.27004	-0.54932
Cl	-2.03912	-2.85339	0.20319
H	2.24287	7.00531	-0.02311
C	1.71689	6.06868	0.11154
C	2.08333	4.95711	-0.63523
H	2.89003	5.02782	-1.35272
C	1.41465	3.75517	-0.46471
H	1.68827	2.88451	-1.04405
C	0.37461	3.67270	0.45554
C	-0.38507	2.41795	0.67052
O	0.18121	1.36232	0.02276
C	-0.52698	0.18390	-0.09910
C	0.00273	4.78800	1.20005
H	-0.81392	4.69660	1.90392
C	0.67682	5.98478	1.02902
H	0.39221	6.85307	1.60843
C	-1.92486	0.31441	-0.62687
C	0.13065	-0.94521	0.17680
H	-2.26148	-0.62528	-1.04357

Cl	-2.00912	1.53421	-1.93115
H	-2.60198	0.64281	0.15694

3.3.4 1,3-haloboration: product R^2=Me (gas)

H	1.74695	-2.89856	0.60136
O	-1.30034	-0.16078	1.73464
B	2.29468	-0.76665	0.03313
C	2.07554	0.73481	0.34035
C	2.42789	1.72753	-0.58259
C	2.15587	3.06233	-0.32873
C	1.56918	3.43426	0.87575
C	1.25789	2.46928	1.82394
C	1.49547	1.12970	1.55081
H	2.90109	1.44239	-1.51549
H	2.40367	3.81588	-1.06574
H	1.35740	4.47757	1.07520
H	0.80842	2.75690	2.76617
H	1.21134	0.37856	2.27857
Cl	3.76680	-1.24388	-0.82988
H	-2.21321	4.96060	-1.40144
C	-2.00115	3.97207	-1.01327
C	-1.31026	3.05590	-1.79430
H	-0.97967	3.32926	-2.78809
C	-1.03562	1.78862	-1.30034
H	-0.48901	1.06997	-1.89562
C	-1.45484	1.44535	-0.01963
C	-1.12473	0.13043	0.58555
O	-0.57179	-0.71695	-0.31868
C	0.01097	-1.89676	0.13432
C	-2.14741	2.36420	0.76514
H	-2.45175	2.07424	1.76270
C	-2.42317	3.62553	0.26609
H	-2.96327	4.34114	0.87236

C	-0.92384	-3.08707	0.12554
C	1.32380	-1.93058	0.36301
C	-0.28439	-4.34397	0.68844
Cl	-1.27215	-3.39555	-1.63999
C	-2.25936	-2.81202	0.80189
H	-2.89733	-3.68749	0.68710
H	-2.76279	-1.95679	0.35656
H	-2.09913	-2.61015	1.86092
H	-1.00365	-5.16024	0.64280
H	-0.00706	-4.17775	1.73141
H	0.59748	-4.63554	0.12148

3.3.5 1,1-carboboration: product R^2=H (gas)

Cl	-3.20282	-0.66847	-0.30753
C	-0.04783	2.90580	1.37313
H	-0.53017	2.28147	2.11330
C	0.09853	2.43448	0.06898
C	0.71727	3.21515	-0.90671
H	0.82392	2.83492	-1.91321
C	1.19030	4.47175	-0.57201
H	1.67253	5.08378	-1.32234
C	0.42788	4.16394	1.69766
H	0.31807	4.53754	2.70672
Cl	-1.52130	-1.92200	1.90543
C	1.04534	4.94416	0.72718
H	1.41640	5.92807	0.98508
O	-0.89033	0.38105	0.64476
O	-0.29874	0.73462	-1.49619
B	-1.49865	-1.01363	0.32992
C	1.95330	-1.19604	-0.56375
H	1.87202	-0.26277	-1.10891
C	3.20238	-1.57778	-0.08712
H	4.05532	-0.93023	-0.24658

C	1.00945	-3.22249	0.28821
H	0.15203	-3.86570	0.44203
C	3.35789	-2.78330	0.57687
H	4.33018	-3.08322	0.94516
C	0.83639	-2.00723	-0.37559
C	2.25410	-3.60817	0.75678
H	2.36291	-4.55706	1.26659
C	-0.70962	-0.73477	-3.22758
C	-0.54091	-0.60158	-1.92721
C	-0.40054	1.09576	-0.25454
C	-0.55339	-1.62463	-0.84637
H	-1.05180	-2.50787	-1.24740
H	-0.87426	-1.71874	-3.63910
H	-0.69579	0.12042	-3.88792

3.3.6 1,1-carboboration: product R^2=Me (gas)

Cl	-2.56532	-0.48202	1.82708
C	0.94394	3.09269	1.19613
H	0.95453	2.54235	2.12758
C	0.35257	2.52081	0.07066
C	0.32463	3.20376	-1.14457
H	-0.13365	2.74426	-2.01003
C	0.89098	4.46380	-1.22972
H	0.87546	4.99999	-2.16929
C	1.50741	4.35356	1.10129
H	1.96778	4.80435	1.97041
Cl	0.07617	-1.64398	2.82261
C	1.48071	5.03672	-0.10870
H	1.92320	6.02241	-0.17954
O	-0.11861	0.54531	1.25732
O	-0.83656	0.73254	-0.86527
B	-0.78477	-0.83918	1.43454
C	1.55577	-1.25972	-1.23587

H	1.18024	-0.35881	-1.70806
C	2.85133	-1.67226	-1.52602
H	3.46478	-1.07931	-2.19311
C	1.25712	-3.17802	0.16286
H	0.63735	-3.76525	0.82964
C	3.35201	-2.84019	-0.97385
H	4.35925	-3.16495	-1.20124
C	0.74174	-2.00011	-0.38050
C	2.54476	-3.59550	-0.13138
H	2.92093	-4.51458	0.30055
C	-2.11226	-0.85981	-1.98927
C	-1.26097	-0.62324	-0.99951
C	-0.22965	1.17968	0.18420
C	-0.66472	-1.56872	-0.01350
H	-1.30618	-2.44933	0.03171
C	-2.57705	-2.26969	-2.24232
H	-3.05915	-2.33757	-3.21696
H	-3.30529	-2.57776	-1.48766
H	-1.74936	-2.97892	-2.22052
C	-2.68265	0.20577	-2.87438
H	-3.77376	0.18618	-2.81134
H	-2.42085	0.00413	-3.91605
H	-2.34213	1.20362	-2.61165

3.3.7 Solvated configurations relating to the free energy diagrams 1,3-haloboration

Reactants

6	1.173152	1.738513	0.056587
6	1.180475	2.482980	-1.131045
6	0.859875	3.830788	-1.130852
6	0.527880	4.462790	0.062633
6	0.519122	3.745316	1.252487
6	0.838121	2.395984	1.247137

5	1.516482	0.234565	0.052497
1	1.434240	1.996330	-2.065087
1	0.868417	4.390725	-2.057529
1	0.276863	5.516727	0.064723
1	0.260673	4.236811	2.181940
1	0.820955	1.840383	2.177118
17	1.969227	-0.604088	-1.428264
17	1.509221	-0.721436	1.527297
1	-2.509709	3.137979	-0.631240
6	-2.292734	2.089722	-0.464717
6	-1.944675	1.276072	-1.535526
1	-1.894089	1.686902	-2.535930
6	-1.644627	-0.061871	-1.324371
1	-1.362780	-0.697367	-2.152874
6	-1.706525	-0.586910	-0.035117
6	-1.409200	-2.017368	0.247588
8	-1.553245	-2.545842	1.318855
8	-0.962547	-2.673131	-0.835943
6	-0.639805	-4.053879	-0.642044
6	-2.060170	0.228084	1.037710
1	-2.095114	-0.197655	2.032507
6	-2.351058	1.565143	0.821582
1	-2.613123	2.202159	1.656848
1	-0.538030	-4.463358	-1.645511
1	-1.464517	-4.551033	-0.138123
6	0.624377	-4.203737	0.079368
6	1.703784	-4.248917	0.598211
1	2.737984	-4.149903	0.838801

Adduct*

6	-0.708391	-1.047605	0.992392
5	-0.770433	-2.557144	0.103768
6	0.761298	-2.983585	-0.036980
6	1.535321	-2.527752	-1.108135
6	2.876400	-2.864125	-1.226114
6	3.479229	-3.667122	-0.266170
6	2.731171	-4.122836	0.810355
6	1.389743	-3.779674	0.923205
1	1.077416	-1.908800	-1.871328
1	3.450600	-2.504072	-2.070815
1	4.523979	-3.936733	-0.358039
1	3.191448	-4.750880	1.563323
1	0.818767	-4.149835	1.766135
17	-1.629325	-2.391134	-1.537703
17	-1.787527	-3.616461	1.234281
1	1.595446	7.237685	-0.132426
6	1.120582	6.268552	-0.046679
6	0.494105	5.707343	-1.152871
1	0.480350	6.236874	-2.096050
6	-0.114314	4.467346	-1.049213
1	-0.604434	4.022677	-1.904361
6	-0.091016	3.794768	0.170714
6	-0.723261	2.475165	0.324178
8	-0.788547	1.841722	1.359493
8	-1.241694	1.962613	-0.797282
6	-1.945635	0.749523	-0.542554
6	0.534728	4.356071	1.281509
1	0.540536	3.814692	2.218280
6	1.141416	5.594952	1.169292
1	1.631199	6.036166	2.027113
1	-2.000636	0.187117	-1.476302

1	-2.948722	0.980833	-0.181325
6	-1.226288	-0.067621	0.424446
1	-0.218336	-1.164629	1.946170

Dioxolium adduct

6	0.241785	0.752784	-0.213379
5	-0.573769	2.141999	-0.254267
6	0.367498	3.413994	0.017323
6	0.676293	3.808689	1.323405
6	1.523241	4.878159	1.582512
6	2.093429	5.585708	0.532323
6	1.814227	5.204402	-0.772439
6	0.966284	4.131101	-1.021228
1	0.230280	3.278807	2.157643
1	1.733883	5.163935	2.606224
1	2.747321	6.426344	0.729293
1	2.250317	5.748683	-1.601691
1	0.750865	3.858518	-2.047455
17	-1.999696	2.084008	1.017265
17	-1.372357	2.205194	-1.981389
1	1.520661	-7.588983	-0.243988
6	1.160558	-6.571874	-0.156061
6	-0.155863	-6.340730	0.229600
1	-0.814473	-7.171995	0.440932
6	-0.622690	-5.044681	0.341526
1	-1.643447	-4.845302	0.639232
6	0.245376	-3.985760	0.066691
6	-0.236915	-2.630659	0.172373
8	0.525139	-1.619685	-0.002148
8	-1.461524	-2.360396	0.439336
6	-1.622397	-0.899729	0.523310
6	1.568589	-4.212339	-0.318710

1	2.222166	-3.376551	-0.529923
6	2.020621	-5.513452	-0.430319
1	3.041066	-5.706118	-0.732247
1	-1.888232	-0.666856	1.550837
1	-2.421825	-0.614765	-0.155109
6	-0.263654	-0.414435	0.102983
1	1.281836	0.755035	-0.530094

Chloride TS*

6	-1.339859	0.368253	-1.112624
17	-1.827183	-1.856855	-1.055644
6	-0.172139	-0.734765	0.856801
5	-0.697470	-2.199176	0.547585
6	0.418292	-3.265342	0.140727
6	1.423308	-2.887242	-0.756930
6	2.424364	-3.766392	-1.141738
6	2.447076	-5.057756	-0.627533
6	1.460550	-5.455314	0.263132
6	0.460497	-4.566195	0.641684
1	1.418459	-1.881391	-1.166303
1	3.189680	-3.446461	-1.838318
1	3.228736	-5.747578	-0.920845
1	1.469167	-6.460185	0.667437
1	-0.300348	-4.891107	1.340717
17	-1.840672	-2.764009	1.905375
1	1.478866	7.424640	0.708867
6	1.116725	6.425958	0.500062
6	0.303337	6.206658	-0.606170
1	0.032319	7.030282	-1.252918
6	-0.161661	4.932150	-0.877869
1	-0.797603	4.740444	-1.731977
6	0.193047	3.880592	-0.034906

6	-0.309694	2.539274	-0.342417
8	0.016485	1.579698	0.491904
8	-1.005519	2.259076	-1.316141
6	1.007487	4.096337	1.075586
1	1.274395	3.269948	1.720085
6	1.468387	5.374903	1.338787
1	2.102862	5.552489	2.196724
1	-0.918721	0.201570	-2.090252
1	-2.395009	0.577213	-1.039780
6	-0.480155	0.314632	0.122534
1	0.466493	-0.558582	1.717887

Product

1	-0.318283	0.329562	2.367143
8	1.880042	-1.615069	-0.430274
5	-0.391396	2.369981	1.378559
6	0.437049	3.329848	0.486736
6	0.018849	4.637919	0.212669
6	0.778565	5.478077	-0.584097
6	1.988412	5.033808	-1.103070
6	2.433719	3.747398	-0.829343
6	1.660534	2.903042	-0.048719
1	-0.916734	4.992768	0.627545
1	0.434032	6.481756	-0.796910
1	2.587558	5.693582	-1.718061
1	3.381647	3.404906	-1.223798
1	2.014595	1.899974	0.163721
17	-1.556185	3.056625	2.523059
1	0.054797	-7.347883	0.439934
6	0.217216	-6.279860	0.369143
6	-0.792331	-5.401585	0.739067
1	-1.740390	-5.783173	1.094309

6	-0.588618	-4.033495	0.651458
1	-1.368717	-3.339777	0.932204
6	0.631042	-3.549279	0.189276
6	0.898655	-2.096487	0.063013
8	-0.094958	-1.345061	0.601985
6	-0.100944	0.018264	0.359850
6	1.641792	-4.428893	-0.186329
1	2.578227	-4.024691	-0.547595
6	1.434125	-5.794302	-0.093621
1	2.218177	-6.481573	-0.382841
6	-0.072539	0.411786	-1.087384
6	-0.246671	0.830494	1.405365
1	-0.361676	1.449142	-1.206326
17	-1.226786	-0.579721	-2.028829
1	0.907421	0.242867	-1.524234

3.3.8 Solvated configurations relating to the free energy diagrams 1,1-carboboration

Reactants

6	1.076028	2.245798	-0.077507
6	0.910987	2.774983	-1.364686
6	0.487622	4.081120	-1.551372
6	0.219443	4.885520	-0.449285
6	0.375340	4.381070	0.836128
6	0.799198	3.073690	1.018102
5	1.548012	0.790631	0.126556
1	1.114439	2.152245	-2.227600
1	0.367204	4.473523	-2.553216
1	-0.110134	5.907514	-0.593028
1	0.166041	5.006858	1.694580
1	0.912542	2.685413	2.023119
17	1.895105	-0.266699	-1.236519
17	1.800959	0.116414	1.729552

1	-2.850188	3.321070	-0.218846
6	-2.495996	2.307722	-0.073913
6	-2.224380	1.504800	-1.174304
1	-2.364548	1.890796	-2.175975
6	-1.758354	0.210523	-0.993186
1	-1.535969	-0.417064	-1.845476
6	-1.572780	-0.282012	0.296365
6	-1.082423	-1.667844	0.551887
8	-0.958304	-2.142242	1.650567
8	-0.796632	-2.315451	-0.584115
6	-0.242951	-3.655027	-0.541028
6	-1.846386	0.523652	1.399707
1	-1.687688	0.123740	2.393187
6	-2.306628	1.816698	1.213193
1	-2.512017	2.445600	2.070250
6	0.090987	-3.954020	-1.998643
6	-1.274465	-4.641573	-0.002969
6	0.994552	-3.673566	0.256328
6	2.030330	-3.733910	0.854066
1	2.946090	-3.771990	1.397076
1	0.533928	-4.945902	-2.076491
1	0.796141	-3.215527	-2.378628
1	-0.824079	-3.920367	-2.590603
1	-0.876849	-5.652314	-0.091423
1	-2.181886	-4.565649	-0.603513
1	-1.505681	-4.439954	1.039061

Adduct*

5	-0.721048	-2.736939	0.526881
6	-1.421348	-1.111567	0.765889
6	-0.714399	0.386675	-1.344363
17	-1.698615	-3.464062	-0.868883

6	0.851347	-2.667296	0.290242
6	1.492221	-3.418267	-0.693922
6	2.867091	-3.335490	-0.884605
6	3.632365	-2.496261	-0.087532
6	3.015398	-1.753492	0.912882
6	1.643818	-1.844828	1.097624
1	0.906728	-4.067812	-1.333940
1	3.339851	-3.926229	-1.659914
1	4.702413	-2.425262	-0.238496
1	3.604373	-1.103124	1.548009
1	1.178459	-1.258355	1.883448
17	-1.127443	-3.598339	2.125725
1	1.059200	7.244864	0.896781
6	0.691017	6.247073	0.693295
6	1.269373	5.490844	-0.318765
1	2.085203	5.897539	-0.901750
6	0.802028	4.213597	-0.582731
1	1.246924	3.618249	-1.368291
6	-0.248057	3.698029	0.173494
6	-0.772477	2.340568	-0.072175
8	-1.695132	1.829388	0.528027
8	-0.129853	1.666465	-1.030065
6	-0.829590	4.454851	1.187837
1	-1.644233	4.034649	1.763157
6	-0.357413	5.730605	1.446031
1	-0.804449	6.322170	2.234171
6	0.387184	-0.437413	-2.007561
6	-1.907695	0.581580	-2.274312
6	-1.132267	-0.264383	-0.097086
1	-2.051556	-1.056846	1.639950
1	-1.563715	1.088550	-3.175306
1	-2.685806	1.171626	-1.793984

1	-2.313306	-0.393851	-2.541207
1	0.651129	0.071140	-2.934350
1	0.019044	-1.436708	-2.234864
1	1.260443	-0.511167	-1.362487

Dioxolium adduct

8	0.522500	1.833597	-1.264642
6	-0.007615	0.443890	-1.516007
6	0.408967	-2.961042	0.398881
6	-1.195389	-0.819271	0.455584
17	-1.627327	-2.730901	-1.671302
5	-1.070164	-2.396325	0.116451
6	1.075064	-3.855539	-0.438562
6	2.354855	-4.314125	-0.143666
6	3.003564	-3.886651	1.005994
6	2.356752	-3.002411	1.861824
6	1.080430	-2.551528	1.556846
1	0.587877	-4.198632	-1.343684
1	2.845637	-5.008534	-0.815321
1	4.000444	-4.241291	1.236788
1	2.847566	-2.667281	2.767774
1	0.589053	-1.868055	2.242533
17	-2.301695	-3.290882	1.259227
1	0.577583	7.348665	1.249705
6	0.438162	6.321313	0.937823
6	1.079174	5.857535	-0.206195
1	1.712475	6.519669	-0.780966
6	0.905399	4.546659	-0.608775
1	1.394734	4.167915	-1.496152
6	0.083851	3.708293	0.147614
6	-0.095066	2.335759	-0.265493
8	-0.878224	1.537153	0.357051

6	-0.562393	4.169357	1.296482
1	-1.192365	3.500726	1.867955
6	-0.380164	5.482483	1.687296
1	-0.872801	5.855220	2.574844
6	1.178878	-0.454460	-1.755548
6	-0.927859	0.580945	-2.713226
6	-0.739717	0.214644	-0.214489
1	-1.675944	-0.557434	1.395985
1	-0.359356	0.932354	-3.574229
1	-1.738526	1.278879	-2.500224
1	-1.353975	-0.396315	-2.930205
1	1.743093	-0.088467	-2.613330
1	0.811663	-1.456832	-1.973055
1	1.819384	-0.504842	-0.875981

Phenyl migration TS*

6	1.306661	1.686761	-0.194197
6	-0.162351	1.366459	-0.484424
6	-2.073495	-0.311765	-0.084101
17	-1.244833	3.203470	1.505503
5	-0.876620	2.721127	-0.138706
6	1.837957	1.583560	1.090434
6	3.167257	1.900489	1.328458
6	3.985207	2.328147	0.289229
6	3.461737	2.440827	-0.991269
6	2.130659	2.124649	-1.228740
1	1.198037	1.241990	1.893960
1	3.567334	1.811068	2.330760
1	5.023226	2.571611	0.477409
1	4.089148	2.771574	-1.809350
1	1.724689	2.210765	-2.230557
17	-1.260585	3.866971	-1.417331

1	1.357881	-6.929966	-0.191426
6	0.957445	-5.925101	-0.150798
6	-0.417686	-5.730937	-0.101557
1	-1.086722	-6.581126	-0.104068
6	-0.935238	-4.447408	-0.049591
1	-2.003496	-4.280689	-0.011606
6	-0.064997	-3.357051	-0.046793
6	-0.599129	-2.011251	-0.000637
8	0.124521	-0.980000	-0.045306
8	-1.867214	-1.776266	0.170768
6	1.317489	-3.549052	-0.094289
1	1.978374	-2.692261	-0.087330
6	1.822536	-4.835190	-0.147018
1	2.892429	-4.992399	-0.183571
6	-3.195393	0.152109	0.812619
6	-2.457483	-0.210530	-1.558318
6	-0.687949	0.201398	0.310811
1	-0.234737	1.224742	-1.573391
1	-3.364036	-0.784669	-1.754105
1	-1.657592	-0.583855	-2.201309
1	-2.646478	0.833016	-1.817644
1	-4.123093	-0.369887	0.572662
1	-3.357166	1.220065	0.654518
1	-2.934739	-0.006661	1.857054

Pincer intermediate

6	-1.277317	0.430942	1.935320
17	-0.972633	3.846000	-1.023375
6	-0.260434	1.115650	-0.308918
5	-1.369911	2.176463	-0.671412
6	1.143969	1.680727	-0.176514
6	1.668531	2.046548	1.059557

6	2.938434	2.603074	1.151278
6	3.702209	2.798068	0.008390
6	3.186776	2.433440	-1.229256
6	1.918106	1.878252	-1.317756
1	1.084121	1.891329	1.959194
1	3.332266	2.881328	2.121262
1	4.693516	3.227527	0.081328
1	3.775276	2.576193	-2.127237
1	1.519038	1.595852	-2.286422
17	-3.040380	1.688267	-0.868805
1	1.890201	-6.628517	-0.709827
6	1.388314	-5.673976	-0.608890
6	0.208998	-5.434234	-1.305482
1	-0.208186	-6.199987	-1.947164
6	-0.433113	-4.213006	-1.177561
1	-1.350554	-4.004889	-1.713178
6	0.103614	-3.231562	-0.348067
6	-0.621343	-1.937594	-0.242972
8	-0.054774	-1.103243	0.648209
8	-1.606626	-1.653518	-0.871960
6	1.283948	-3.470962	0.351457
1	1.695706	-2.702271	0.991457
6	1.924644	-4.693927	0.217639
1	2.843089	-4.882574	0.758867
6	-1.766818	1.803510	2.295128
6	-1.563777	-0.625619	2.962353
6	-0.623079	0.172345	0.808190
1	-0.276756	0.504570	-1.225922
1	-1.035647	-0.397734	3.892292
1	-1.277142	-1.621123	2.633563
1	-2.631860	-0.626208	3.195170
1	-1.497553	2.020217	3.332089

1	-2.856502	1.858255	2.223973
1	-1.338609	2.599757	1.684102

Product

17	-2.547835	-0.535854	1.813600
6	0.810480	3.165757	1.301043
1	0.817310	2.607852	2.228004
6	0.286158	2.582729	0.147017
6	0.263227	3.282075	-1.060537
1	-0.142967	2.815550	-1.948024
6	0.768195	4.569753	-1.108503
1	0.756059	5.119510	-2.040364
6	1.312839	4.454347	1.242329
1	1.719706	4.914851	2.132887
17	0.111697	-1.632018	2.830529
6	1.291459	5.153646	0.040602
1	1.685305	6.161605	-0.001191
8	-0.122890	0.564294	1.288299
8	-0.791919	0.756565	-0.847872
5	-0.742545	-0.825880	1.424649
6	1.684022	-1.211160	-1.120218
1	1.345233	-0.273143	-1.546531
6	2.982231	-1.632801	-1.386751
1	3.631839	-1.012967	-1.992710
6	1.296518	-3.205618	0.146383
1	0.637725	-3.823535	0.745411
6	3.439954	-2.843550	-0.889307
1	4.448693	-3.175570	-1.100135
6	0.824076	-1.987496	-0.344835
6	2.588158	-3.631426	-0.122995
1	2.930917	-4.582958	0.264546
6	-1.941339	-0.831414	-2.075212

6	-1.161572	-0.612072	-1.022125
6	-0.234349	1.218300	0.219230
6	-0.590941	-1.554741	-0.014775
1	-1.229808	-2.436854	0.021005
6	-2.382333	-2.222061	-2.426374
1	-2.391876	-2.342091	-3.510292
1	-3.402262	-2.397469	-2.072059
1	-1.735606	-2.987725	-2.003412
6	-2.457373	0.295893	-2.929092
1	-2.829571	-0.087209	-3.877690
1	-1.684980	1.036851	-3.132484
1	-3.280949	0.810635	-2.428032

4 Crystallographic studies.

Crystallographic studies were undertaken on single crystal mounted in paratone and studied on an Agilent SuperNova Dual three-circle diffractometer using Cu-Kα or Mo-Kα radiation and a CCD detector. Measurements were typically made at 150(2) K with temperatures maintained using an Oxford cryostream. Data were collected and integrated and data corrected for absorption using a numerical absorption correction based on gaussian integration over a multifaceted crystal model within CrysAlisPro.[14] The structures were solved by direct methods and refined against F^2 within SHELXL-2013.[15] The structures have been deposited with the Cambridge Structural Database (CCDC deposition numbers 1582714-1582717).

Table S8. Refinement metrics for **3c** and **4a**–**c**.

Compound	**3c**	**4a**	**4b**	**4c**
Empirical Formula	$C_{16}H_{12}BCl_2NO_4$	$C_{18}H_{17}BCl_2O_2$	$C_{19}H_{19}BCl_2O_2$	$C_{18}H_{16}BCl_2FO_2$
Crystal System	Triclinic	Monoclinic	Monoclinic	Monoclinic
Space Group	*P*-1	$P2_1/n$	$P2_1/n$	$P2_1/n$
a/Å	7.1918(4)	13.1814(5)	10.8458(4)	10.4734(3)
b/Å	10.3441(6)	8.4219(4)	13.8708(5)	14.0452(4)
c/Å	13.0804(7)	15.3446(8)	12.3961(5)	12.0480(4)
α/°	96.791(4)	90	90	90
β/°	100.323(5)	97.278(4)	103.821(4)	100.970(3)
γ/°	92.295(4)	90	90	90
V/Å^3	948.75(9)	1689.71(13)	1810.89(12)	1739.88(9)
Z	2	4	4	4
T /K	150(2)	150(2)	150(2)	150(2)
D_c/g.cm^{-3}	1.274	1.364	1.324	1.393
Crystal size/mm	0.413 x 0.324 x 0.274	0.232 x 0.203 x 0.055	0.257 x 0.182 x 0.154	0.237 x 0.217 x 0.096
Total data	6804	5940	6210	6325
Unique data	4012	3173	3409	3073
R_{int}	0.0401	0.0460	0.0253	0.0233
$R_1[F^2>2\sigma(F^2)]$	0.0381	0.0457	0.0336	0.0296
wR2 (all data)	0.0930	0.1304	0.0954	0.0787
GoF	1.022	1.043	1.033	1.045
ρ_{min}/ρ_{max}/eÅ^{-3}	-0.291/0.266	-0.408/0.446	-0.349/0.349	-0.253/0.291
CCDC code	1582714	1582715	1582716	1582717

S67 Thermal ellipsoid plot (50% probability) of solid-state structure of **3c**.

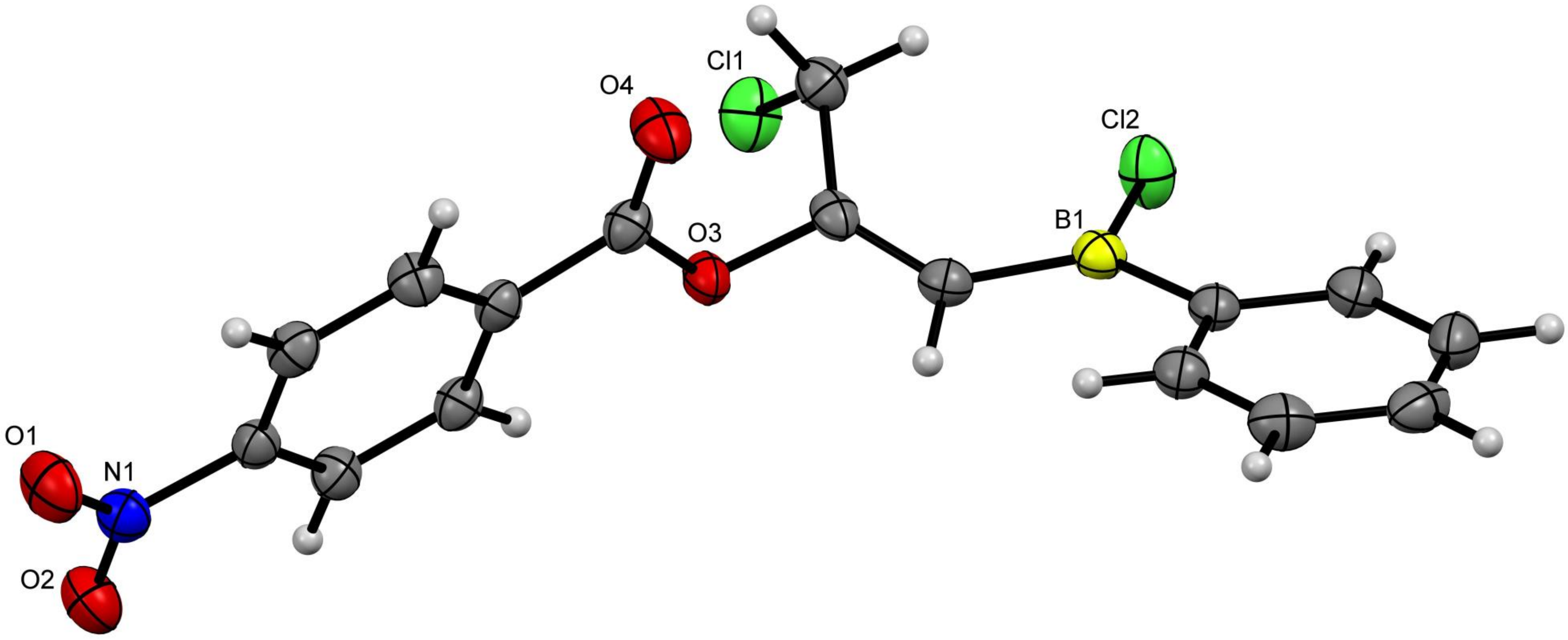

S68 Thermal ellipsoid plot (50% probability) of solid-state structure of **4a**.

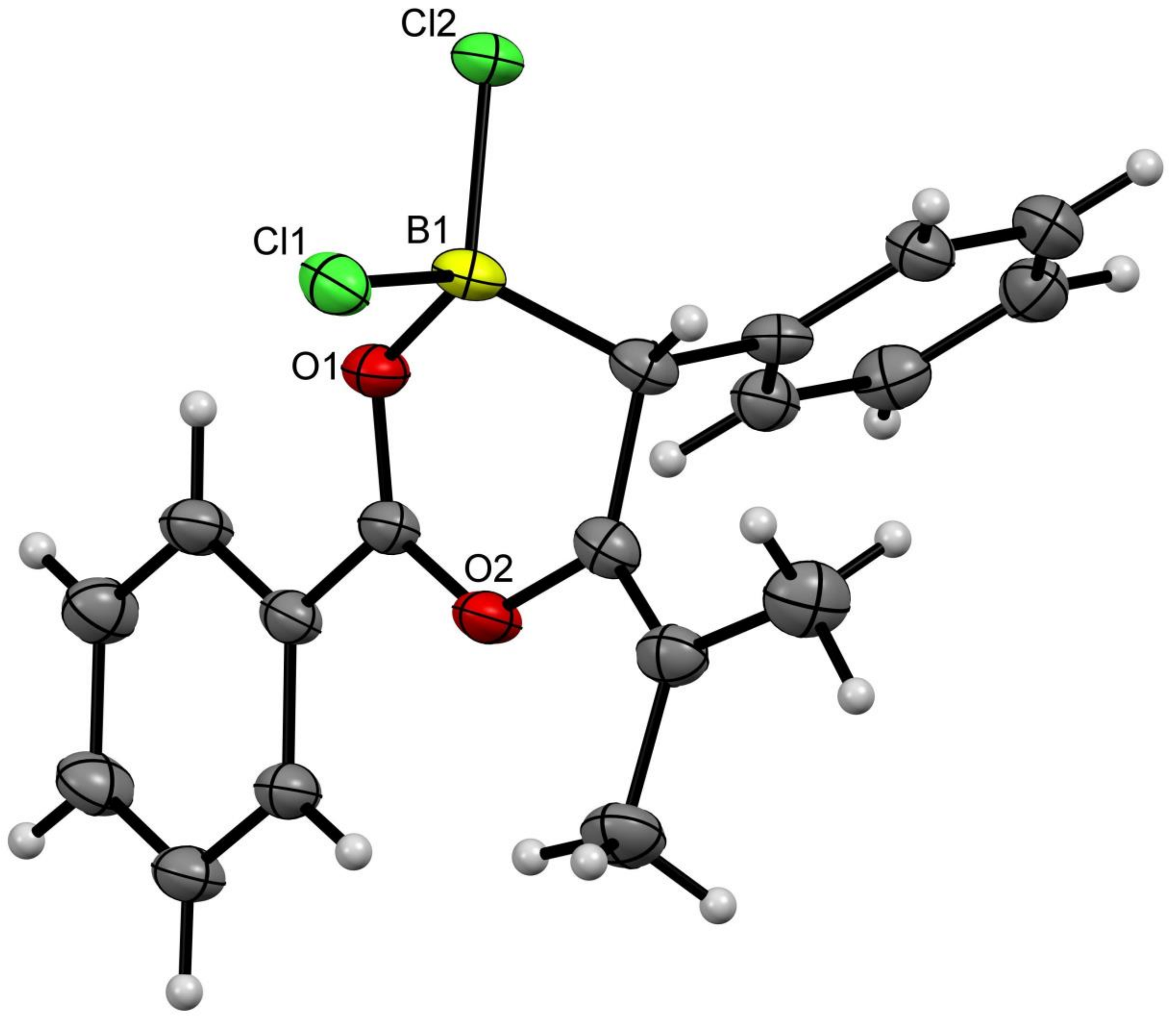

S69 Thermal ellipsoid plot (50% probability) of solid-state structure of **4b**.

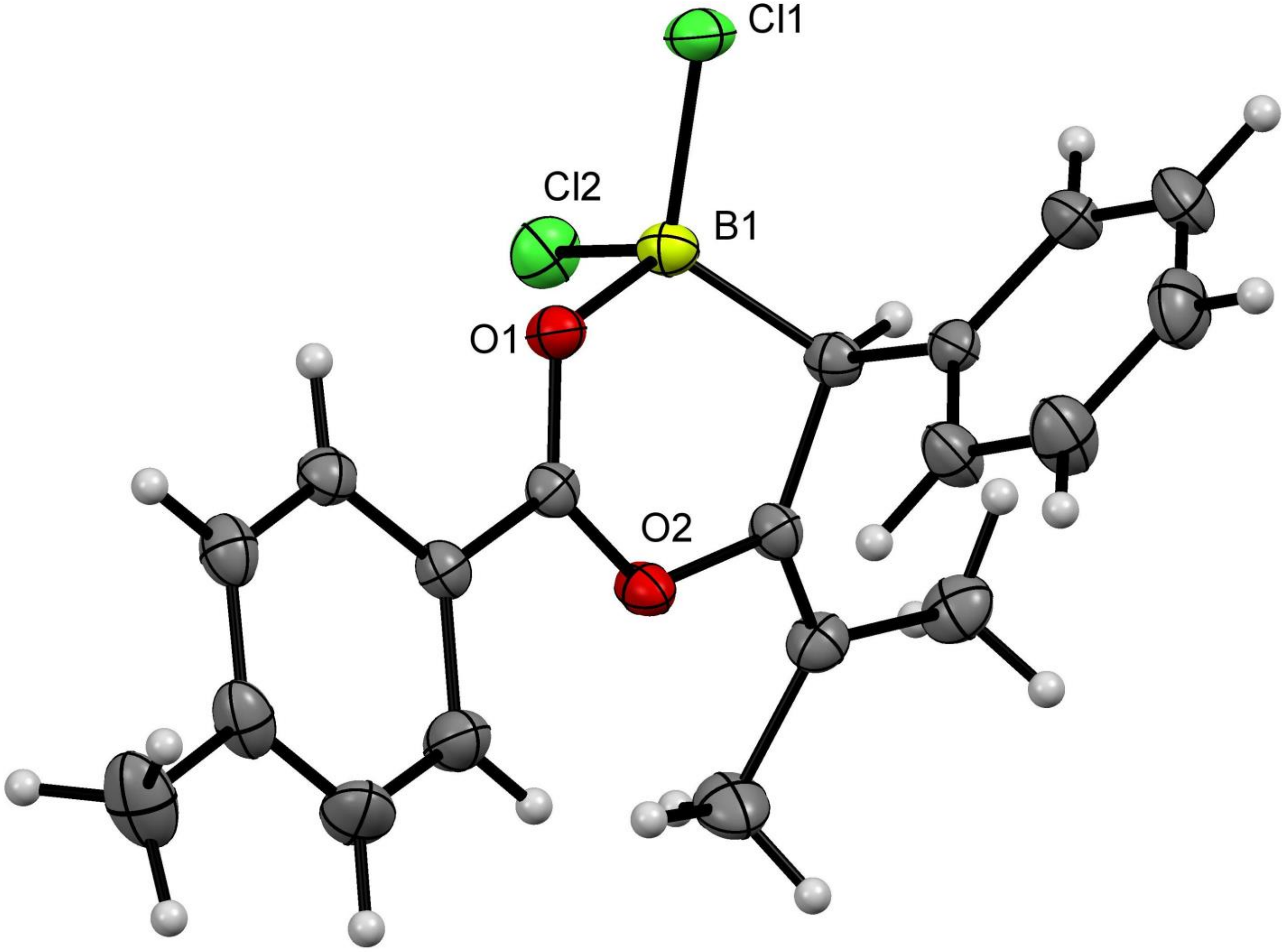

S70 Thermal ellipsoid plot (50% probability) of solid-state structure of **4c**.

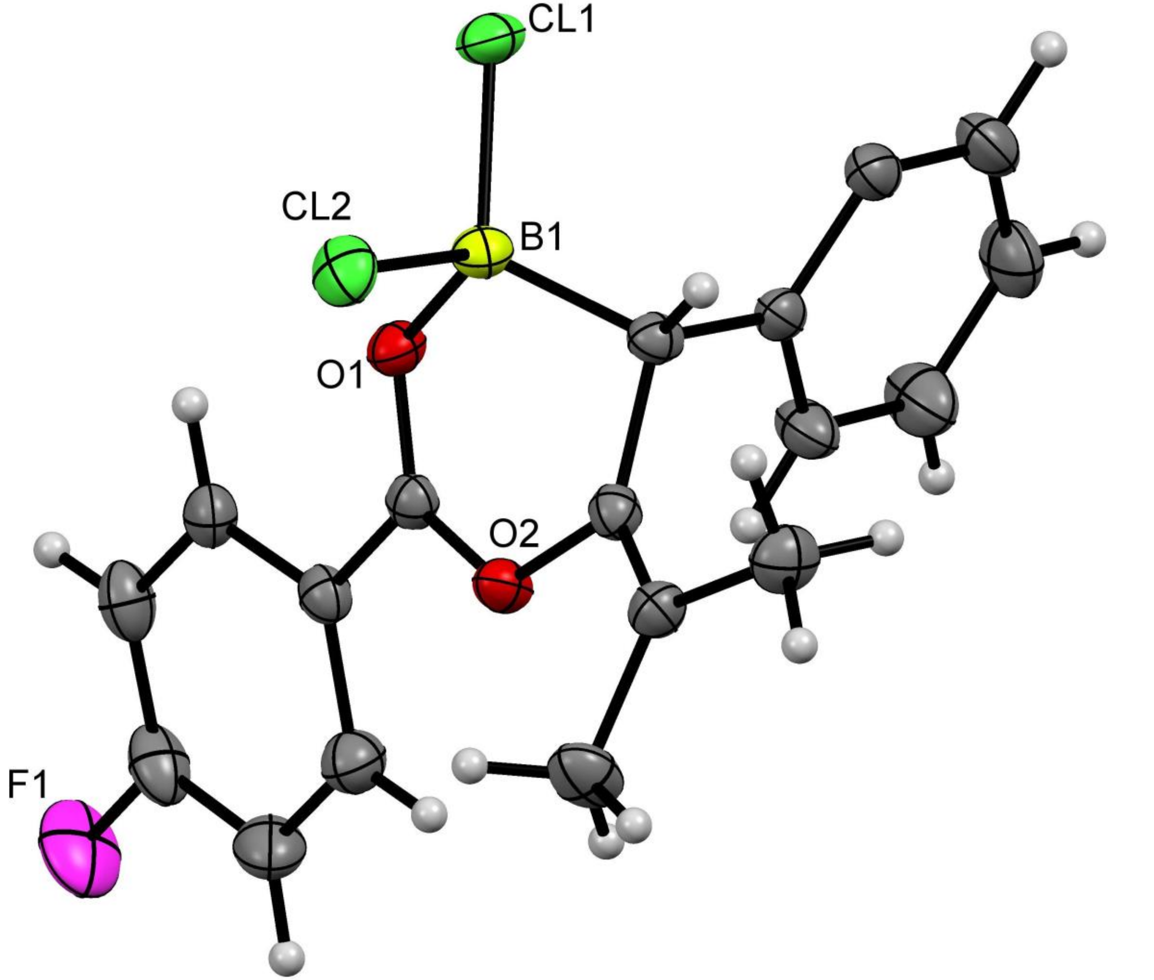

References.

[1] M. M. Hansmann, R. L. Melen, F. Rominger, A. S. K. Hashmi, D. W. Stephan, *J. Am. Chem. Soc.* **2014**, *136*, 777-782.

[2] L. C. Wilkins, J. R. Lawson, P. Wieneke, F. Rominger, A. S. K. Hashmi, M. M. Hansmann, R. L. Melen, *Chem. Eur. J.* **2016**, *22*, 14618-14624.

[3] C. P. Kaushik, K. Kumar, D. Singh, S. K. Singh, D. K. Jindal, R. Luxmi, *Synth. Commun.* **2015**, *45*, 1977-1985.

[4] B. L. Harris, J. M. White, *Aust J Chem* **2014**, *67*, 1866-1870.

[5] T. Yamada, K. Park, Y. Monguchi, Y. Sawama, H. Sajiki, *RSC Adv.* **2015**, *5*, 92954-92957.

[6] V. V. Pagar, A. M. Jadhav, R.-S. Liu, *J. Am. Chem. Soc.* **2011**, *133*, 20728-20731.

[7] Z. Chen, Y. Wen, Y. Fu, H. Chen, M. Ye, G. Luo, *Synlett*, **2017**, *28*, 981-985.

[8] M. Valiev, E. J. Bylaska, N. Govind, K. Kowalski, T. P. Straatsma, H. J. J. Van Dam, D. Wang, J. Nieplocha, E. Apra, T. L. Windus, et al., *Comput. Phys. Commun.* **2010**, *181*, 1477–1489.

[9] Y. Zhao, D. G. Truhlar, *Theor. Chem. Acc.* **2007**, *120*, 215–241.

[10] Y. Zhao, D. G. Truhlar, *J. Phys. Chem. A* **2005**, *109*, 5656–5667.

[11] L. Goerigk, S. Grimme, *Phys. Chem. Chem. Phys.* **2011**, *13*, 6670.

[12] N. Mardirossian, M. Head-Gordon, *Mol. Phys.* **2017**, *115*, 2315–2372.

[13] S. Grimme, J. Antony, S. Ehrlich, H. Krieg, *J. Chem. Phys.* **2010**, *132*, 154104.

[14] CrysAlisPro, Agilent Technologies, Version 1.171.37.33 (release 27-03-2014 CrysAlis171 .NET).

[15] SHELXL-2013, G. M. Sheldrick, University of Göttingen, Germany (2013).
